# Targeting the ERO1A–PDIA1 redox axis in triple-negative breast cancer with AI-designed minibinders

**DOI:** 10.1038/s41420-026-03301-w

**Published:** 2026-08-10

**Authors:** Alice Marrazza, Sara Baroni, Ersilia Varone, Alessandro Cherubini, Serena Germani, Giorgia Maria Renna, Daniele Generali, Manuela Milani, Mirko Marabese, Monica Favagrossa, Alessia Porta, Giorgia Giordano, Alfredo Cagnotto, Luca Guarrera, Marco Gobbi, Bo-Ram Jin, Jaehyung Cho, Marten Beeg, Ester Zito

**Affiliations:** 1 Istituto di Ricerche Farmacologiche Mario Negri IRCCS, Milan, Italy; 2 Department of Biomolecular Sciences, University of Urbino Carlo Bo, Urbino, Italy; 3 U.O. Patologia Mammaria e Tumori Cerebrali, Azienda Socio-Sanitaria Territoriale, Cremona, Italia; 4 Division of Hematology, Department of Medicine and Department of Pathology and Immunology, Washington University School of Medicine, St. Louis, USA

**Keywords:** Breast cancer, Breast cancer

## Abstract

Endoplasmic reticulum oxidoreductin 1 alpha (ERO1A), together with its partner protein disulfide isomerase A1 (PDIA1), promotes oxidative protein folding within the endoplasmic reticulum (ER), thereby supporting ER redox homeostasis, proteostasis, and cancer progression. Importantly, ERO1A enhances the aggressiveness and therapy resistance of triple-negative breast cancer (TNBC), while PDIA1 is also upregulated and positively correlates with ERO1A expression in TNBC. These observations suggest that targeting the ERO1A–PDIA1 interaction may simultaneously impair the activity of both proteins, thereby providing a dual-hit anti-cancer strategy. Guided by AI-based structural modeling of the ERO1A–PDIA1 interface, we used the BindCraft pipeline to design and rank one hundred de novo protein minibinders targeting the b′ domain of PDIA1, from which eight top-scoring, non-redundant candidates were selected for experimental characterization. In vitro biochemical assays using recombinant proteins demonstrated direct binding of three minibinders (mb) to PDIA1 and inhibition of both the ERO1A–PDIA1 electron transfer relay and the intrinsic reductase activity of PDIA1. Furthermore, liposome-mediated intracellular delivery of mb7 impaired TNBC cell viability, providing proof-of-concept evidence that the minibinder can restrain tumor cell survival. Overall, our work provides a rationale for the development of highly specific and stable protein-based modulators of the ERO1A–PDIA1 interaction, which, by simultaneously inhibiting the activities of both ERO1A and PDIA1, may represent an effective strategy to restrain TNBC progression and counteract therapy resistance.

**Figure:**
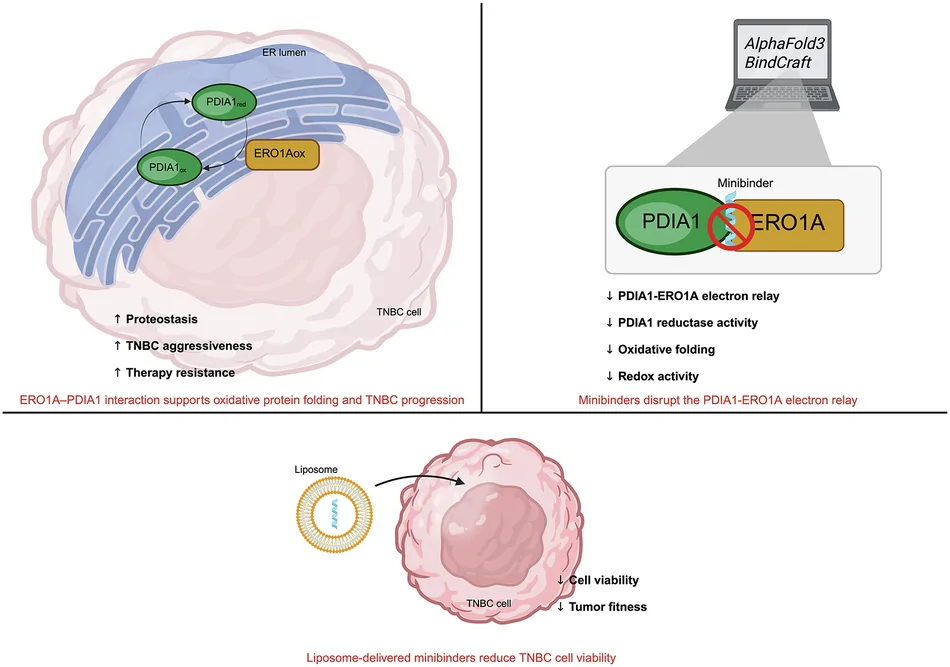

## Introduction

Endoplasmic reticulum oxidoreductin 1 alpha (ERO1A, hereafter referred to as ERO1) acts by reoxidizing its partner protein disulfide isomerase A1 (PDIA1, hereafter referred to as PDI), thereby promoting the formation of disulfide bonds in newly synthesized proteins within the endoplasmic reticulum (ER) through the reduction of molecular oxygen [1–3]. PDI contains two catalytic thioredoxin-like a and a’ domains, and two non-catalytic b and b’ domains. [4]. ERO1 binds to the b’a’ domain of PDI through multiple contact points and preferentially oxidizes the a’ domain [5]. Val101 of ERO1 contributes to interaction with the catalytic domain of PDI and is required for efficient electron transfer. Additionally, the β-hairpin region of ERO1 containing Trp272, interacts via non-covalent interactions with Phe240, Phe249, and Phe304 in the b’ domain of PDI, residues that form the canonical hydrophobic substrate-binding pocket of the b′ domain [6, 7]. These interactions position Cys397 and Cys400 in the a’ domain active site of PDI proximal to the disulfide-bonded cysteines in the shuttle loop of ERO1 and thereby allow ERO1-mediated electron relay. The electron relay between ERO1 and PDI promotes oxidative protein folding in nascent proteins and thus maintains redox homeostasis and proteostasis within the ER [8]. ERO1 activity can be compensated by PRDX4, GPX7 and GPX8 in healthy cells [9–12], and its genetic inhibition does not trigger an overt pathological phenotype [10]. However, ERO1 activity is essential in tumor cells under hypoxic conditions, promoting angiogenesis, glycolysis and contributing to increased invasiveness and resistance to therapy [7, 13–21]. Accordingly, ERO1 genetic deletion or inhibition has been shown to impair TNBC growth by targeting vascular endothelial growth factor A (VEGFA) signaling and its N-glycosylation [15, 22] or PD-1/PD-L1 immune checkpoints in the tumor microenvironment [23, 24]. On the other hand, PDI also helps cancer cells to cope with increased protein demand, aiding invasion and migration by contributing to tumor angiogenesis and remodeling of the tumor microenvironment [25]. Together, the concerted roles of ERO1 and PDI in cancer progression, along with their upregulation in TNBC, have spurred our interest in developing specific disruptors of the ERO1–PDI interaction that, by simultaneously impairing the activity of both proteins, may represent a more effective therapeutic strategy in cancer in general and TNBC in particular. In recent years, advances in computational protein design have enabled the generation of minibinders, small hyperstable proteins typically composed of 50–60 amino acids, which exhibit highly specific binding to target proteins [26], thereby limiting off-target effects and showing promise as therapeutic agents [27]. Therefore, we used computational design to generate de novo protein minibinders that, by binding to PDI, disrupt the ERO1–PDI interaction. We found that disruption of the ERO1–PDI interaction in a highly specific, tailored manner by a minibinder is effective in impairing both ERO1 and PDI redox activities and restraining TNBC.

## Results

### High PDI levels in tumors

**Fig. 1:**
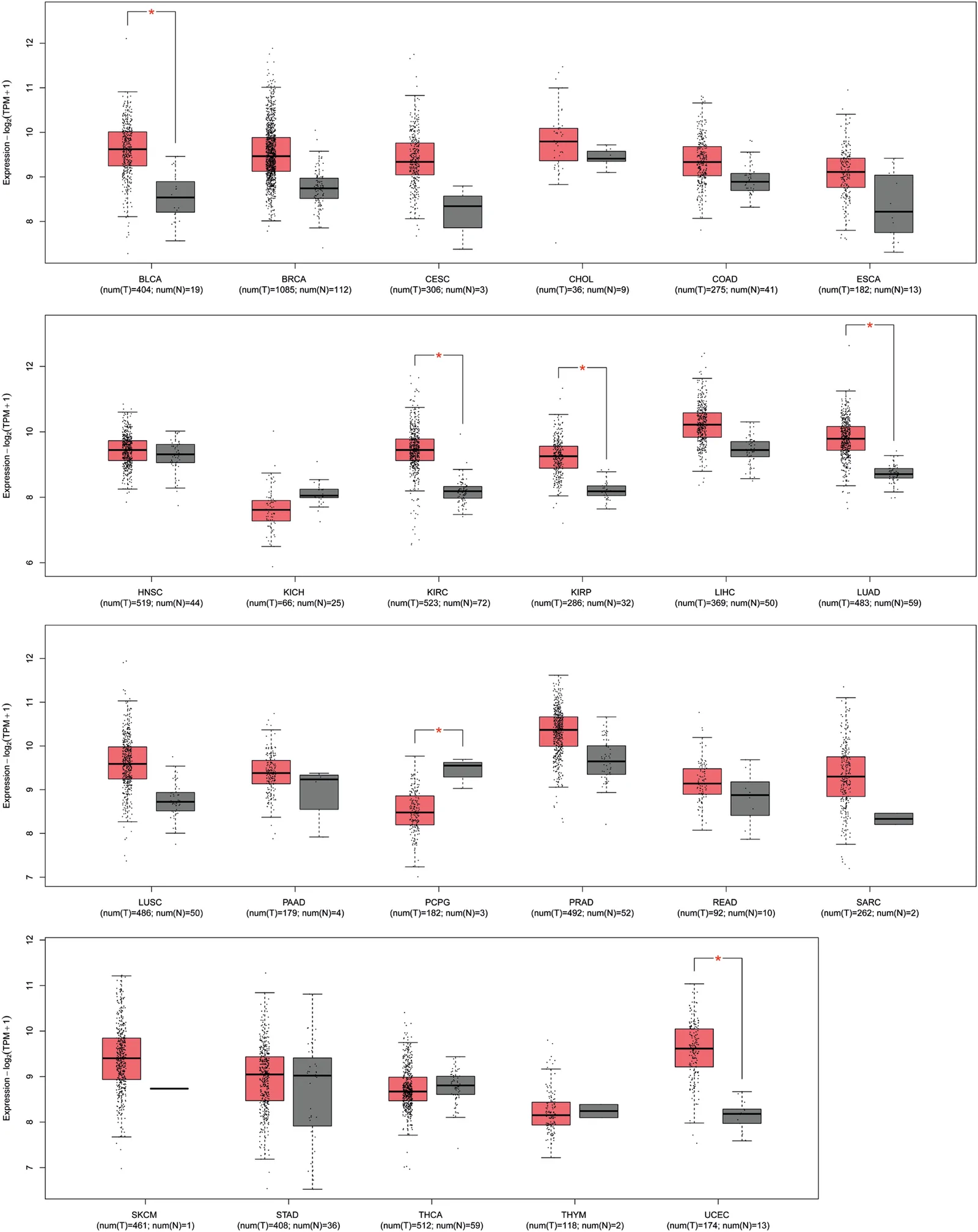
Comparative expression of PDI (P4HB) across multiple cancers. Box-plot representation illustrating the differential expression of the P4HB gene across various tumor types in the TCGA dataset. Tumor samples are shown in red, while adjacent normal tissues are depicted in gray. Each box on the x-axis represents a specific cancer type, and the y-axis indicates gene expression values normalized as log2(TPM + 1). TPM (Transcripts Per Million) is a normalization method that accounts for RNA composition, providing a standardized measure of gene expression in each sample. Red: tumor, Gray: adjacent normal tissue; in parentheses is highlighted the number of Tumors (T) and normal tissue (N) analyzed. BLCA Bladder Urothelial Carcinoma, BRCA Breast Invasive Carcinoma, CESC Cervical squamous cell carcinoma and endocervical adenocarcinoma, CHOL Cholangiocarcinoma, COAD Colon adenocarcinoma, ESCA Esophageal carcinoma, HNSC Head and Neck squamous cell carcinoma, KICH Kidney Chromophobe, KIRC Kidney renal clear cell carcinoma, KIRP Kidney renal papillary cell carcinoma, LIHC Liver hepatocellular carcinoma, LUAD Lung adenocarcinoma, LUSC Lung squamous cell carcinoma, PAAD Pancreatic adenocarcinoma, PCPG Pheochromocytoma and Paraganglioma, PRAD Prostate adenocarcinoma, READ Rectum adenocarcinoma, SARC Sarcoma, SKCM Skin Cutaneous Melanoma, STAD Stomach adenocarcinoma, THCA Thyroid carcinoma, THYM Thymoma, UCEC Uterine Corpus Endometrial Carcinoma.

To investigate whether PDI (P4HB) expression is broadly altered in cancer, we analyzed RNA expression data from tumor and matched normal tissues available in The Cancer Genome Atlas (TCGA). This analysis revealed that PDI mRNA levels are elevated in most tumor types compared with their corresponding normal tissues, indicating that PDI upregulation is a common feature of malignant transformation (Fig.1).

### PDI expression is elevated in TNBC and correlates with ERO1

**Fig. 2:**
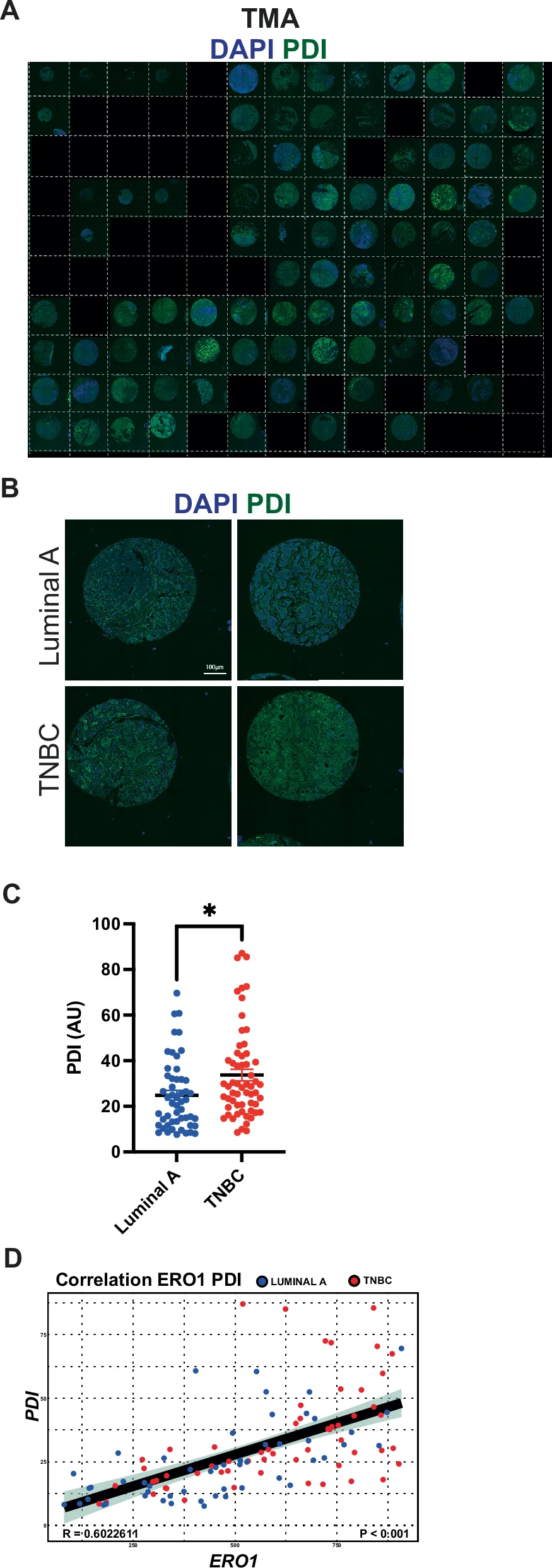
Higher expression of PDI in TNBC and positive correlation between ERO1 and PDI in human breast cancers. **A** A tissue microarray (TMA) containing breast cancer samples, of which 48 were classified as Luminal A and 52 as TNBC, was analyzed by immunofluorescence for PDI, with nuclei stained using DAPI. **B** Representative zoomed images showing PDI and DAPI staining in the indicated Luminal A and TNBC samples. **C** Dot plots representing the quantification of PDI staining in Luminal A and TNBC TMA samples (mean ± SEM; unpaired t-test; PDI: *P* = 0.0084). Each point represents an individual sample. **D** Scatter plot showing the relationship between ERO1 (x-axis) and PDI (y-axis) expression across TMA samples, stratified by subtype: Luminal A (blue) and TNBC (red). A fitted linear regression line with confidence interval is overlaid. ERO1 and PDI levels are positively correlated (R = 0.602, *P* < 0.001).

To experimentally assess PDI expression levels in breast tumors, we analyzed a tissue microarray (TMA) comprising one hundred human breast cancer samples previously classified as Luminal A or TNBC. PDI expression was evaluated by immunofluorescence staining (Fig. 2A, B and Supplementary Fig. 1). PDI levels were significantly higher in the more aggressive TNBC subtype, similarly to what was observed for ERO1 [23] (Fig. 2C). Notably, PDI expression positively correlated with ERO1 levels in both Luminal A and TNBC tumors (Fig. 2D), indicating that ERO1 and PDI are upregulated in parallel in these breast cancers [23].

### Mapping the ERO1–PDI interacting region

**Fig. 3:**
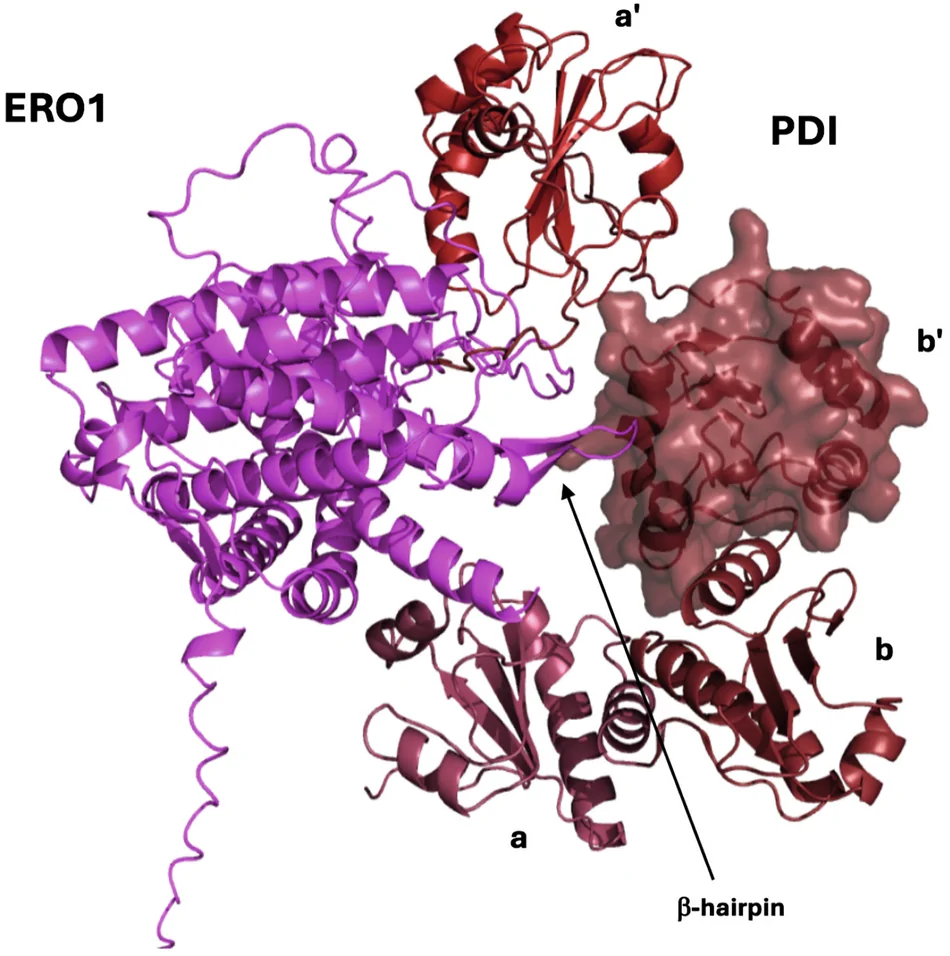
AlphaFold 3-predicted structural model of the ERO1–PDI complex. The predicted complex shows ERO1 (magenta) interacting with PDI (dark red), with individual PDI domains labeled as a, b, b′, and a′. The β-hairpin region of ERO1 engages the b′ domain of PDI, which is depicted as a semi-transparent surface to highlight the hydrophobic substrate-binding pocket.

To enable the rational design of a disruptor targeting the ERO1–PDI interface, we first sought to define the molecular interaction between ERO1 and PDI. Because an experimental co-crystal structure of the ERO1–PDI complex is not currently available, we employed AlphaFold3, a recently developed AI-based structural prediction framework that enables high-confidence modeling of protein–protein complexes and provides improved resolution of interacting regions compared with earlier approaches. The AlphaFold3-predicted model delineated the ERO1–PDI interaction interface and was consistent with previous reports describing the insertion of the ERO1 β-hairpin into the hydrophobic substrate-binding pocket of the PDI b′ domain (Fig. 3) [6]. This approach allowed us to identify and map putative contact surfaces and key interacting regions between the two proteins, providing structural insights into the ERO1–PDI binding interface. The resulting model offered a reliable framework to guide subsequent computational design and experimental strategies aimed at disrupting the ERO1–PDI interaction.

### Generation of minibinders targeting the PDI b′ domain interacting with ERO1

**Fig. 4:**
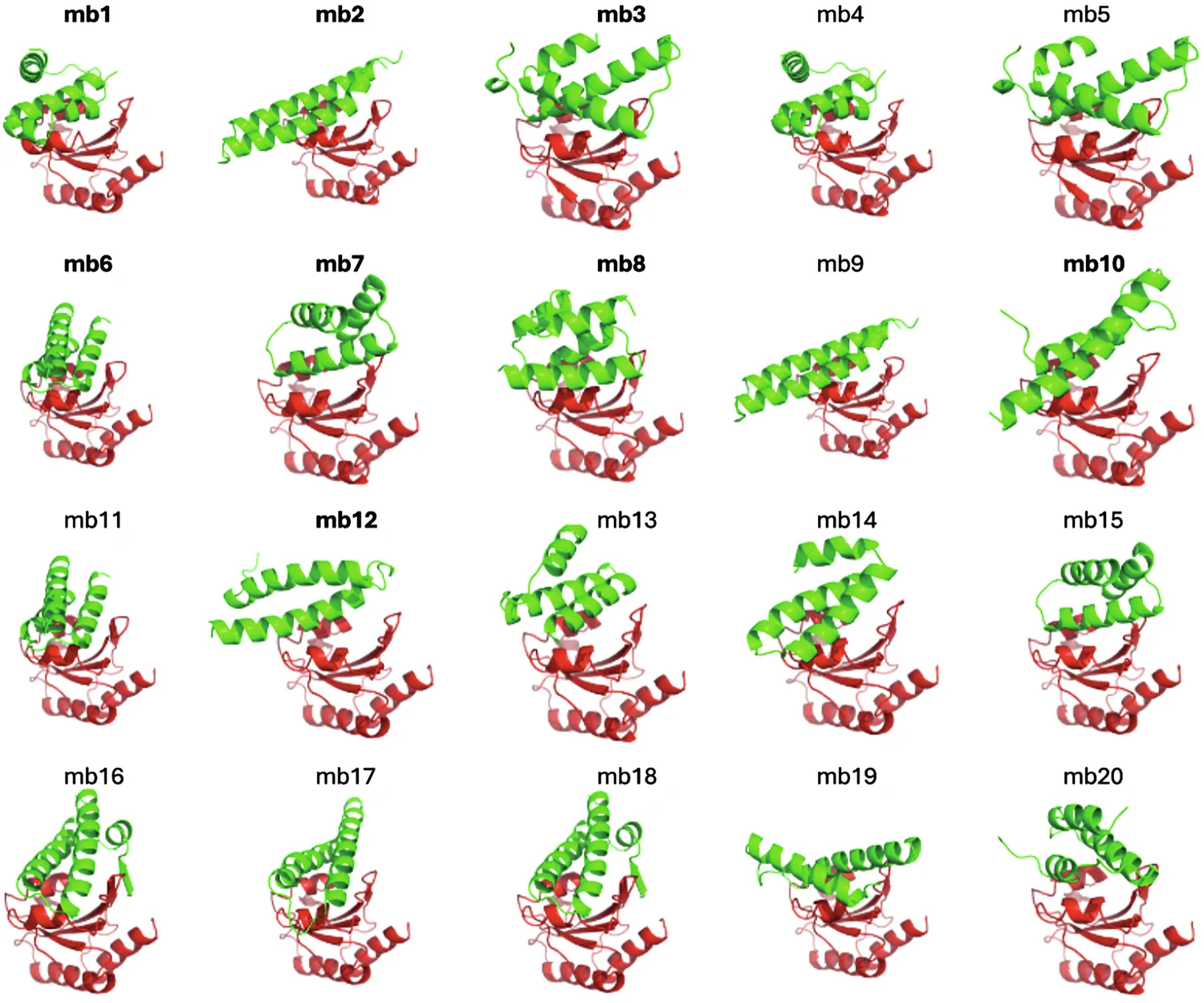
Structural models of designed miniproteins bound to the PDI b′ domain. Structural models of 20 designed minibinders (green) in complex with the PDI b′ domain (red), arranged from left to right and top to bottom according to predicted template modeling (pTM) score. The IDs of designs selected for recombinant expression are highlighted in bold. For each distinct structural scaffold, up to two sequence variants were generated using solubility-optimized Protein MPNN weights, and only the variant with the highest ipTM score was retained. Some designs (e.g., 1 and 4) share an identical backbone but differ in sequence. Constructs selected for experimental characterization correspond to mb1, mb2, mb3, mb6, mb7, mb8, mb10, and mb12 and are indicated in bold.

To identify protein binders capable of engaging the b′ domain of PDI that interacts with the β-hairpin of ERO1, we applied the BindCraft design pipeline to generate de novo minibinder candidates. Using BindCraft, 100 minibinders targeting the b′ domain of PDI successfully passed computational filtering. Designs were ranked by predicted template modeling (pTM) score, and redundancy was removed by retaining only the highest-ranking sequence per structural scaffold. Structural models of the selected designs depict their predicted binding to the b′ domain of PDI (Fig. 4). From this non-redundant ranked set, the top eight minibinders (mb1, 2, 3, 6, 7, 8, 10, 12) were selected for recombinant expression and experimental characterization.

### Expression of minibinders as recombinant proteins and their biophysical characterization

**Fig. 5:**
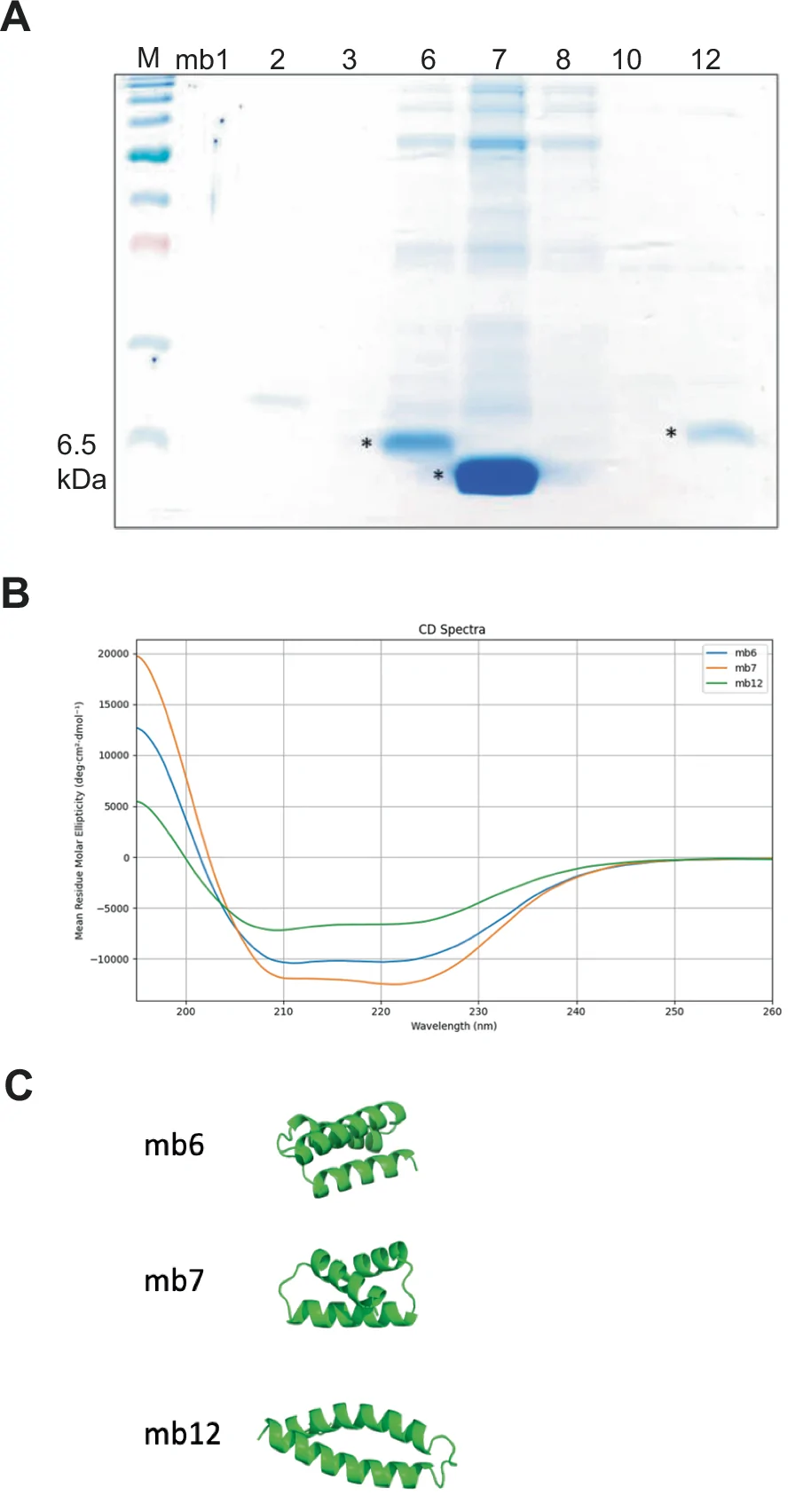
Biochemical and biophysical characterization of minibinders expressed as recombinant proteins. **A** Coomassie-stained SDS-PAGE showing the minibinders (mb) expressed in bacteria as recombinant proteins. M indicates a protein ladder. **B** Far-UV circular dichroism (CD) spectra of mb6, mb7, and mb12, expressed as mean residue molar ellipticity. The spectra display characteristics typical of α-helical secondary structure, consistent with the predicted structural models. **C** Structural models of mb6, mb7, and mb12, illustrating their α-helical fold as predicted by AlphaFold2.

The cDNAs corresponding to the eight selected minibinders were synthesized and sub-cloned into a bacterial expression vector, which was then used to transform BL21 bacterial cells for recombinant expression. Coomassie-stained SDS-PAGE analysis showed that minibinders mb6, mb7, and mb12 were recovered at high yield and migrated at the expected molecular weight of approximately 6.5 kDa (Fig. 5A). Based on their high yield, mb6, mb7, and mb12 were selected for further biophysical characterization. All other minibinders (mb1, mb2, mb3, mb8, and mb10) exhibited low yield under the tested conditions and were therefore not pursued further.

To assess the folding and secondary structure of minibinders mb6, mb7, and mb12, circular dichroism (CD) spectroscopy was performed. All three miniproteins exhibited far-UV CD spectra characteristic of proteins with α-helical secondary structure, showing well-defined minima in the 208–222 nm range (Fig. 5B).

While minor differences in spectral intensity were observed among the minibinders, the overall spectral profiles were consistent with the intended design of compact, properly folded proteins (Fig. 5C). These results indicate that mb6, mb7, and mb12 adopt stable secondary structures in solution, supporting their suitability for subsequent functional analyses.

### Biochemical characterization of minibinders targeting the ERO1–PDI redox axis

**Fig. 6:**
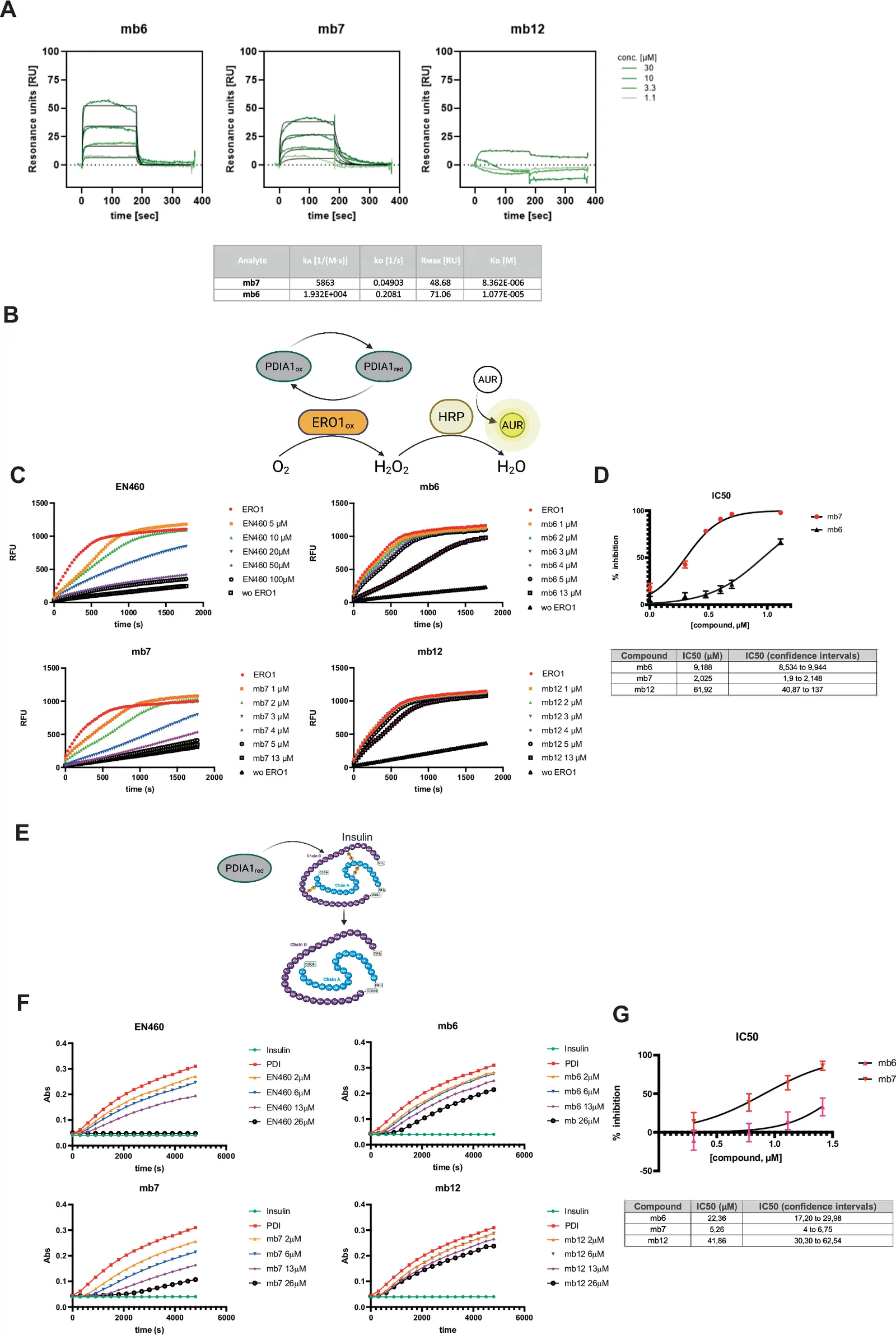
Physical association of minibinders with PDI and minibinder-mediated inhibition of the ERO1–PDI electron relay and PDI reductase activity. **A** Surface plasmon resonance (SPR) analysis of minibinder binding to PDI. SPR sensorgrams showing the binding of minibinders mb6, mb7, and mb12 to immobilized PDI. Recombinant PDI was immobilized on a sensor chip, and minibinders were injected as serial dilutions ranging from 1.1 to 30 μM under oxidizing conditions. Binding responses are shown as resonance units (RU) over time. Below, a table reports the biophysical parameters of the interaction between PDI and the minibinders (K_A_, R_max_ and K_D_). **B** Schematic of the Amplex UltraRed (AUR) assay: the electron transfer between recombinant reduced PDI and ERO1 is measured using a fluorescence-based assay that detects H₂O₂ production. Horseradish peroxidase (HRP) utilizes the H₂O₂ generated by ERO1 to oxidize Amplex UltraRed (AUR) into a fluorescent product. **C** Time course of AUR fluorescence (RFU) in reactions containing ERO1, PDI, and increasing concentrations of the indicated minibinders. “ERO1” indicates AUR reactions containing all components except minibinders, whereas “wo ERO1” indicates reactions containing all components except both minibinders and ERO1. **D** Inhibition plots of the indicated minibinders: The percentage of inhibition of PDI-ERO1 electron relay is plotted against the logarithm of minibinder concentration. Below, a table summarizing the IC₅₀ values of the minibinders derived from the linear phase of the reaction. **E** Schematic of the PDI-mediated insulin turbidimetric assay. The reductase activity of PDI is assessed based on the reduction of insulin disulfide bonds, leading to insulin unfolding and precipitation, which can be measured spectrophotometrically. **F** Time course of insulin turbidity in reactions containing insulin, PDI, and the minibinders. **G** Inhibition plots of the indicated minibinders: The percentage of PDI inhibition is plotted against the logarithm of minibinder concentration. Below, a table summarizing the IC₅₀ values of the minibinders calculated from the inhibition of PDI reductase activity.

To characterize the interaction of the minibinders with PDI, we first assessed binding using surface plasmon resonance (SPR) (Fig. 6A). Minibinders mb6 and mb7 exhibited clear, concentration-dependent binding to immobilized PDI, enabling kinetic analysis using a 1:1 binding model. Global fitting yielded low-micromolar apparent affinities, with mb7 displaying a KD of 8.36 × 10⁻⁶ M (ka = 5.86 × 10³ M⁻¹ s⁻¹; kd= 4.90 × 10⁻² s⁻¹) and mb6 a KD of 10.8 × 10⁻⁶ M (ka = 1.93 × 10⁴ M⁻¹ s⁻¹; kd = 2.08 × 10⁻¹ s⁻¹). Minibinder mb12 generated a detectable SPR signal only at the highest analyte concentration tested, preventing reliable kinetic fitting under these experimental conditions.

To assess the functional impact of the minibinders on PDI activity and on the PDI–ERO1 redox axis, we next employed enzymatic assays. Minibinders mb6, mb7, and mb12 were tested at concentrations ranging from 10 nM to 13 μM for their ability to inhibit the ERO1–PDI electron relay using an AUR fluorescence–based kinetic assay with recombinant ERO1 and PDI proteins together with EN460, a known ERO1 inhibitor [28] (Fig. 6B). The linear phase of the reaction, measured in the absence or presence of different minibinder concentrations, was used to determine the IC₅₀, i.e., the concentration of minibinder that inhibits 50% of the reaction rate (Fig. 6C, D). The IC₅₀ of EN460 was confirmed to be approximately 6 μM; mb6 showed an IC₅₀ of approximately 9 μM, whereas mb7 was more potent with an IC₅₀ of approximately 2 μM. In contrast, mb12 exhibited only weak activity, with an IC₅₀ of approximately 62 μM.

To evaluate whether the minibinders also affected the reductase activity of PDI, they were tested at concentrations ranging from 2 to 26 μM using the classical PDI-mediated insulin turbidimetric assay, performed in kinetic mode and measuring PDI-dependent insulin reduction over time (Fig. 6E, F). The calculated IC₅₀ values were approximately 9 μM for EN460, 22 μM for mb6, 5 μM for mb7, and 41 μM for mb12 (Fig. 6G).

Taken together, these results demonstrate that the tested minibinders physically associate with PDI, impair both the ERO1–PDI electron relay and the reductase activity of PDI, and identify mb7 as the most potent inhibitor. Tables 1, 2.

**Table 1:** Amino acid sequence of selected minibinders.

ID	Sequence
mb1	METVREMVNRFSDEEWAKMSPREGYDWVMYALYKQAGEEPDEPTLAVVKKLEAERAALEHHHHHH
mb2	MSEENRKEVKELIKIVEDLHESYVKKVKESKDGKSEEEKEKEIKEHEKEMEKFKKVLEPLTKEKPLEHHHHHH
mb3	MPGDRSKYTWAEIFVAAITLWDAEMRKKKEEGIEVSKEEQHELKKKMWEIAYEDPEKALELTKLEHHHHHH
mb6	MEKKKKLKEEMEKIVEEMKEVVDPNNPEEVALVWIAEVMVRHNEEPTERSLETLKIFLNYLKRLEHHHHHH
mb7	MSIEEKIREVHEKLGLTPEQKVEFVREFMILYFTTKKEVDEVLEEVVKKVLEKLEHHHHHH
mb8	MSKETYEYIKKSIENMKKRKMSYESVVELTQFFPIPEKEKENIKNNKPPKELEPIFEEMKKMLEHHHHHH
mb10	MEPSPYDMVMEALYLLENYPLKGESKVEPGMPEEERRKKYEEGQKMLVEAAKRQDELDAALEHHHHHH
mb12	MKKLTPIEQRVEEKLKEREKEYEEKYGDMPEENKKEYIELERIVDEMSIRHEIKNELELEHHHHHH

**Table 2:** Nucleotide sequence of the minibinders.

mb1	ATGGAGACAGTTAGAGAAATGGTAAATAGGTTCAGCGACGAGGAGTGGGCGAAAATGTCCCCGCGTGAAGGCTACGACTGGGTTATGTATGCCCTGTACAAGCAGGCGGGTGAAGAGCCGGATGAGCCGACCTTGGCTGTGGTCAAAAAGCTGGAAGCGGAACGCGCAGCGCTCGAGCACCACCACCACCACCACTGA
mb2	ATGTCAGAAGAAAATAGGAAGGAGGTAAAAGAACTGATCAAAATTGTTGAAGACCTGCATGAAAGCTACGTTAAAAAGGTGAAGGAGTCCAAAGATGGTAAAAGCGAAGAAGAGAAGGAGAAGGAGATCAAGGAGCACGAAAAGGAGATGGAAAAGTTCAAAAAAGTGCTGGAACCGTTGACCAAAGAGAAGCCGCTCGAGCACCACCACCACCACCACTGA
mb3	ATGCCCGGAGATAGGTCAAAATATACATGGGCTGAGATCTTCGTGGCGGCAATTACGCTGTGGGACGCTGAAATGCGTAAAAAGAAGGAGGAGGGTATTGAAGTTAGCAAAGAGGAGCAGCACGAGCTGAAAAAGAAGATGTGGGAAATCGCCTACGAAGATCCGGAAAAGGCGCTGGAATTGACCAAACTCGAGCACCACCACCACCACCACTGA
mb6	ATGGAAAAAAAAAAGAAACTAAAGGAGGAAATGGAAAAAATCGTGGAAGAGATGAAAGAGGTTGTCGACCCGAACAACCCGGAGGAGGTGGCGTTGGTTTGGATTGCTGAAGTGATGGTTCGTCACAACGAGGAGCCGACGGAACGTAGCCTGGAAACCCTGAAGATCTTCTTAAATTACCTGAAGCGCCTCGAGCACCACCACCACCACCACTGA
mb7	ATGTCAATTGAAGAGAAAATAAGGGAAGTACACGAGAAGCTGGGTCTGACCCCGGAACAGAAAGTGGAATTTGTTCGTGAATTCATGATCCTGTACTTCACCACGAAAAAGGAGGTGGACGAGGTCTTAGAGGAAGTTGTTAAAAAGGTGTTGGAGAAGCTCGAGCACCACCACCACCACCACTGA
mb8	ATGTCAAAAGAGACTTATGAATACATAAAGAAATCCATTGAAAATATGAAGAAACGTAAAATGAGCTACGAGAGCGTTGTGGAATTGACCCAGTTTTTCCCGATCCCGGAAAAGGAGAAGGAGAACATTAAAAACAACAAACCGCCAAAAGAGCTGGAACCGATCTTCGAGGAAATGAAGAAGATGCTCGAGCACCACCACCACCACCACTGA
mb10	ATGGAACCATCACCCTATGATATGGTAATGGAGGCGCTGTATTTGCTGGAAAACTACCCGCTGAAGGGTGAAAGCAAAGTTGAGCCGGGTATGCCGGAAGAGGAGCGCCGTAAAAAGTACGAGGAGGGCCAAAAGATGTTGGTGGAAGCTGCCAAACGTCAGGACGAACTGGATGCAGCGCTCGAGCACCACCACCACCACCACTGA
mb12	ATGAAAAAGCTAACACCCATAGAACAAAGGGTTGAAGAGAAGCTGAAAGAGCGCGAAAAGGAGTACGAAGAGAAGTATGGTGATATGCCGGAAGAGAACAAAAAGGAGTACATTGAATTGGAGCGTATCGTGGACGAAATGAGCATTCGTCACGAGATCAAAAATGAACTGGAACTCGAGCACCACCACCACCACCACTGA

### ERO1–PDI minibinders reduce breast cancer cell viability

**Fig. 7:**
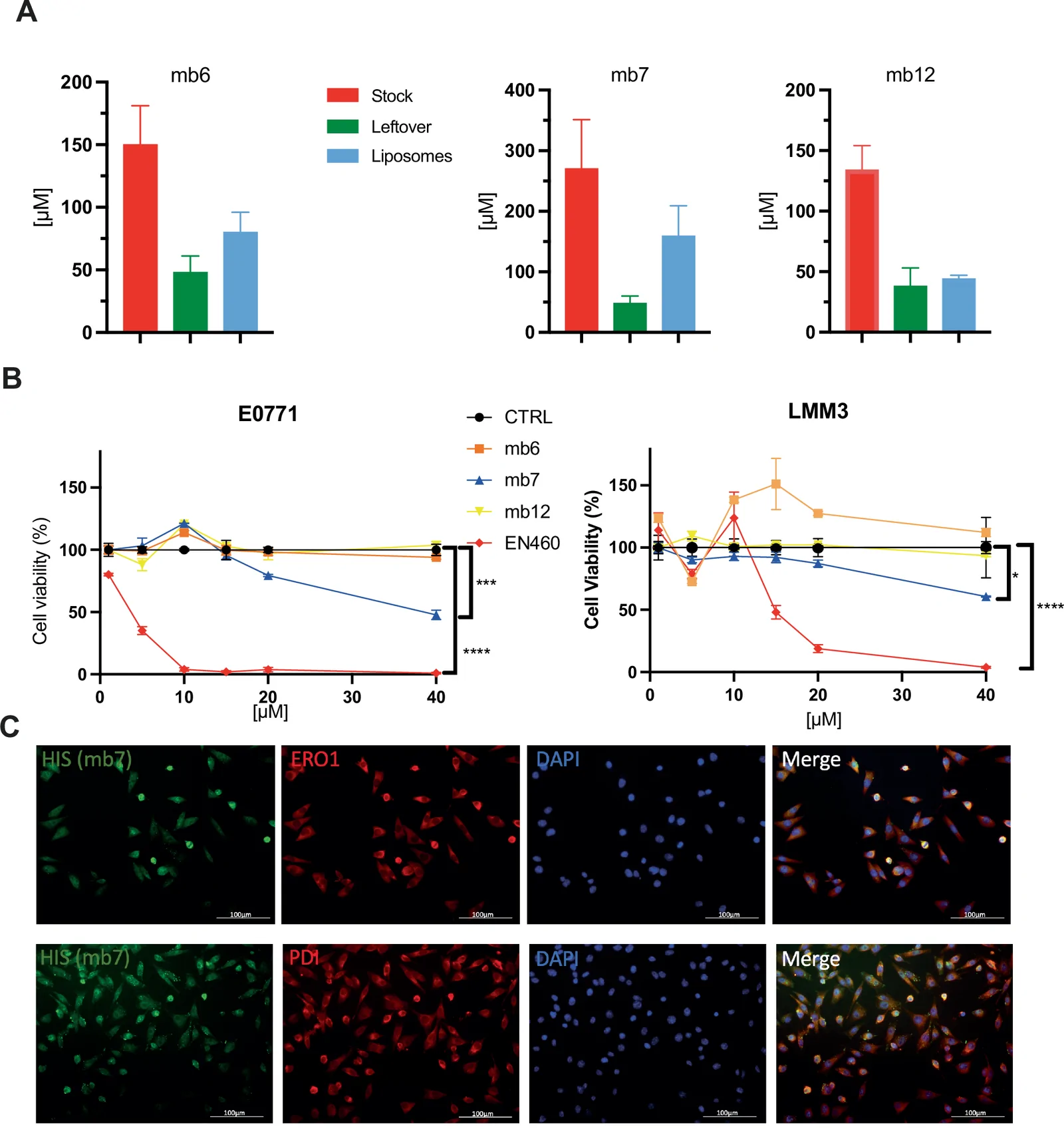
Cytotoxic activity of liposome-encapsulated minibinders in breast cancer cells. **A** Bar graphs showing minibinder concentrations quantified before liposome encapsulation, the amount remaining in the supernatant after encapsulation (unencapsulated fraction), and the fraction successfully encapsulated within liposomes, as determined by MALDI–TOF mass spectrometry (*N* = 6; mean ± SD). **B** MTS-based cell viability assay of E0771 and LMM3 cells treated with the ERO1 inhibitor EN460, liposome-encapsulated minibinders, or empty liposomes (CTRL). Cell viability in untreated conditions was set to 100%, and values at increasing concentrations were expressed as relative percentages (*N* = 3; mean ± SEM; two-way ANOVA versus CTRL). **C** Immunofluorescence analysis of His-tagged mb7 together with ERO1 and PDI in E0771 cells. Nuclei were counterstained with DAPI.

To evaluate the activity of the minibinders in breast cancer cells, we focused on the breast cancer cell line E0771 and LMM3. To improve cellular uptake, minibinders were encapsulated into liposomes composed of a mixture of cholesterol and phosphatidylcholine. After encapsulation, the amount of minibinder effectively incorporated into the liposomes was quantified by MALDI-TOF mass spectrometry and was estimated to be approximately 50% of the initially loaded dose (Fig. 7A). The liposome-encapsulated minibinders were then administered to cells over a range of concentrations (1–40 μM). In parallel, cells were treated with EN460, which served as a positive control for the detection of reduced cell viability, as well as with empty liposomes to control for potential liposome-associated toxicity. Cell viability was assessed 48 hours after treatment using the MTS assay (Fig. 7B).

Cell viability analyses revealed that, starting from a theoretical mb7 concentration of 20 μM (corresponding to the amount encapsulated in liposomes), mb7 reduced the viability of E0771 cells by up to ~50%. Importantly, empty liposomes did not exert detectable cytotoxic effects, indicating that the observed reduction in cell viability was attributable to the encapsulated minibinder rather than to the delivery system itself. As expected, treatment with the small-molecule ERO1 inhibitor EN460 at 20 μM resulted in a near-complete loss of cell viability, further validating the sensitivity of the assay (Fig. 7B).

After 16 hours of incubation with liposome-encapsulated His-tagged mb7, immunofluorescence analysis of the His tag showed that only a few cells contained mb7 (Fig. 7C).

Consistently, semi-quantitative MALDI–TOF mass spectrometry analysis of intracellular mb7 pointed out that, even at the highest concentration of liposome-encapsulated mb7 tested (theoretically 40 μM), the intracellular concentration was lower than 8 μM (Supplementary Fig. 2), thus providing a plausible explanation of the limited effect on cell viability.

Together, these results provide proof-of-concept evidence that disruption of the ERO1–PDI interaction by a specifically designed minibinder can impair breast cancer cell viability, thereby supporting the therapeutic potential of targeting the ERO1–PDI interaction.

## Discussion

ERO1 is the main disulfide oxidase of the ER, which, through its partner PDI, introduces disulfide bonds in newly synthesized proteins. ERO1 is a component of the unfolded protein response (UPR), a homeostatic signaling pathway that can be hijacked by cancer cells to survive under stressful conditions [29, 30]. Indeed, although ERO1 is dispensable in healthy cells, its activity is essential in cancer cells, where it promotes tumor aggressiveness and therapy resistance [13]. Over the years, we have focused our studies on the role of ERO1 in TNBC, an aggressive subtype of breast cancer that lacks targeted therapies and frequently develops resistance to chemotherapy [31]. We have shown that genetic deletion of ERO1 in tumor models restrains TNBC growth [14–16, 23, 24]. Furthermore, novel pyrazolone-based small-molecule inhibitors of ERO1 repress TNBC progression by reducing the expression of the angiogenesis master regulator VEGFA and lowering immune checkpoint levels in the tumor microenvironment [23, 24]. Notably, both genetic ablation of ERO1 and its pharmacological inhibition using pyrazolone- and non-pyrazolone-based compounds have also been shown to be beneficial in non-cancer pathologies, such as SEPN1-related myopathy (SEPN1-RM), cardiac arrhythmia, arterial thrombosis, and ischemic stroke, highlighting the broader therapeutic relevance of targeting ERO1 [24, 32–34].

Here, we analyzed PDI expression levels in breast cancer patient samples by immunofluorescence staining of tumor tissue microarrays and identified elevated PDI expression in TNBC. Moreover, PDI expression positively correlates with ERO1 levels and increases in TNBC and in higher-grade breast tumors [23]. These findings, together with previous studies, led us and others to hypothesize that disruption of the ERO1–PDI complex may represent an effective therapeutic strategy for TNBC [35]. In addition, simultaneous inhibition of two mediators within the same pathway is a well-established approach to overcome drug resistance [36], a hallmark of TNBC. Because minibinders, unlike small molecules, offer high target specificity, they may reduce potential off-target effects, which have been associated with the cysteine-targeting mechanisms of ERO1 inhibitors [24, 33, 37]. We therefore focused on the development of protein-based minibinder disruptors of the ERO1–PDI interaction, using the BindCraft computational design pipeline. We designed one hundred minibinders and selected eight candidates for experimental validation. Our results demonstrate that computational protein design can generate stable minibinders capable of disrupting the ERO1–PDI interaction. Among the recombinant minibinders tested, mb7 exhibited the strongest binding to PDI and most effectively blunted both the ERO1–PDI electron relay and the reductase activity of PDI. Furthermore, liposome-mediated delivery of mb7 into cells significantly reduced breast cancer cell viability. Although we acknowledge that the clinical translation of protein-based minibinders remains challenging, our findings provide a proof-of-concept strategy for TNBC based on targeting the ERO1–PDI interface with a highly specific minibinder that, by impairing the redox relay between PDI and ERO1, simultaneously inhibits both cancer-promoting proteins. Given that ERO1 and PDI are upregulated in multiple cancer types, this approach may be broadly applicable beyond TNBC.

### Limitations

Although liposome-mediated delivery enabled intracellular administration of the minibinders, the effect of mb7 on cancer cell viability remained limited. This is likely attributable, at least in part, to the delivery system, which was not optimized to maximize cellular uptake or efficient endoplasmic reticulum targeting. Improvements in liposome formulation, loading efficiency, stability, and cellular uptake will be important for future translational development and may significantly enhance the therapeutic potential of these protein-based inhibitors. The optimization of the delivery platform was beyond the scope of the present work, whose primary objective was to establish the feasibility of disrupting the ERO1–PDI interaction as a strategy to simultaneously inhibit both proteins and restrain tumor cell growth.

## Materials and methods

### PDI expression in cancer

PDI (P4HB) data were sourced from the GEPIA2 platform [38], utilizing the TCGA dataset [39]. Gene expression levels were reported as transcripts per million (TPM), a normalization method that accounts for sequencing depth and RNA composition, allowing for reliable comparison of gene expression across samples [40].

### Breast cancer patient and tumor collection

Methods were performed in accordance with the relevant guidelines and regulations.

The tissue specimens (breast carcinoma samples) were obtained at the U.O. “Patologia Mammaria e Tumori Cerebrali, Azienda Socio-Sanitaria Territoriale of Cremona, Italy” as part of routine procedures between 2005 and 2008 and after informed consent was obtained. Human studies were conducted in accordance with approval from the local ethical committee of “Azienda Socio-Sanitaria Territoriale of Cremona” (RaLCTrVs1Ott_2000). Breast cancers were classified as Luminal A or triple-negative breast cancer (TNBC) by a pathologist using H&E staining combined with molecular profiling. Residual tissue material was used to generate a human tissue microarray (TMA), which was anonymized prior to analysis to ensure investigator blinding.

### Breast cancer tissue microarray

A TMA comprising 52 TNBC cases and 48 Luminal A cases was generated using a computer-assisted tissue microarrayer (Model TMA Galileo CK4500, Integrated Systems Engineering srl, Milano, Italy). Tissue cores of either 1.0 mm or 0.6 mm in diameter were extracted from FFPE blocks from areas on consecutive H&E-stained sections.

### PDI expression analysis in breast cancer tissues

Five-μm-thick tumor sections were incubated at 70 °C for 40 minutes, then deparaffinized in xylene and rehydrated through graded ethanol solutions (100%, 95%, 85%, and 75%). Antigen retrieval was performed by heating the sections in citrate buffer (pH 6; Biocare Medical, CB910M) using a microwave oven for 15 minutes.

Slides were treated with 1% H₂O₂ for 10 minutes at room temperature (RT), followed by three washes in 0.01 M PBS for 5 minutes each. Non-specific binding was blocked using 10% normal goat serum (NGS) and 0.3% Triton X-100 in PBS for 1 hour. Sections were incubated overnight at 4°C with a monoclonal anti-PDIA1 antibody (MA3-019, Invitrogen) diluted 1:100. After washing, sections were incubated with a biotinylated secondary antibody (1:200 in PBS containing 1% NGS) for 1 hour at RT. Following additional washes in PBS and TNT buffer (0.04% Tween-20 in 0.1 M TBS), slides were incubated with a blocking reagent (AKOYA Biosciences, NEL701A001KT) for 1 hour and 30 minutes at RT. Sections were then incubated with streptavidin-HRP (1:100) in blocking reagent for 30 minutes at RT. Signal amplification was achieved by incubation with fluorescein (1:300) in amplification diluent (AKOYA Biosciences, NEL701A001KT) for 8 minutes at RT. Nuclear counterstaining was performed using Hoechst (1 μg/ml) for 10 minutes. After final washes in PBS and sterile water, sections were mounted using ProLong Gold antifade reagent. Images were acquired using a Nikon A1 confocal microscope with a 20× objective, employing large-area scanning and avoiding signal saturation. PDIA1 fluorescence intensity was quantified across the entire tissue section and expressed as mean gray pixel intensity. Digital image analysis was performed using custom-developed ImageJ algorithms. Correlation analysis between ERO1A (whose values were in ref. [23]) and PDIA1 expression was performed using the ERRE software package.

### AlphaFold3 modeling of the ERO1–PDI complex

Structural modeling of the ERO1A–PDIA1 complex was performed using the AlphaFold3 server (https://alphafoldserver.com/). As input, the amino acid sequences of ERO1α (UniProt Q96HE7, residues 19–466) and PDI (UniProt P07237, residues 18–508) were provided to the server as separate protein chains. Model prediction was carried out using the default AlphaFold3 server settings, which generate a predicted complex structure based solely on the input sequences.

### Minibinder design of the PDI b′ domain

De novo protein minibinders targeting the b′ domain of PDIA1 were designed using the BindCraft computational pipeline. BindCraft is a deep learning–based framework that leverages backpropagation through AlphaFold2 (AF2) to jointly design binder backbone, sequence, and interface [41]. The crystal structure of human PDIA1 (PDB ID: 6I7S) was used as the design input. Binder design was restricted to residues 236–350, corresponding to the b′ domain of PDIA1. All remaining regions of the protein were masked during the design process to confine binder generation to the b′-domain surface. Conditional design was applied by specifying PDI residues Phe249, Phe297, Phe304, and Met324 as ERO1 interface hotspots to bias binder generation toward the b′-domain. Binder lengths were constrained to a range of 55–65 amino acids. Designed binders were further processed by sequence optimization using Soluble Protein MPNN, followed by confidence-based filtering using AF2 monomer predictions. Filtering was performed using the default BindCraft parameters [41]. Designs were ranked based on predicted template modeling (pTM) scores. From the ranked list, the top eight designs were selected for experimental characterization. When multiple sequence variants corresponded to the same binder backbone, the variant with the highest pTM score was selected.

### Minibinder expression as recombinant protein and purification

CDNA coding sequences of the eight selected minibinders with a C-terminal hexahistidine (6×His) tag were synthesized and cloned by GenScript into the pET21b (+) expression vector.

### Minibinder expression and purification

Plasmids were transformed into BL21(DE3) (NEB), and 50 mL of single-colony culture was grown in Luria-Bertani and induced with 0.5 mM IPTG at 20 °C overnight. Cells were collected by spinning for 30 min at 4000 *g*. Pellets were resuspended in lysis buffer (50 mM Na2HPO4 pH 7.5, 200 mM NaCl, 0.2% Triton) containing complete Mini EDTA-free protease inhibitor (Sigma-Aldrich). After cell disruption by sonication, the lysed cells were clarified via centrifugation at 14,000 *g* for 30 min. The supernatant was run over Nickel–NTA resin (Cytiva) pre-equilibrated in Binding buffer (50 mM Na2HPO4 pH 8.0, 300 mM NaCl, 20 mM Imidazole). Extensive washing was performed with washing buffer (50 mM Na2HPO4 pH 8.0, 300 mM NaCl, 40 mM Imidazole) and then the miniproteins were eluted with elution buffer (50 mM Na2HPO4 pH 8.0, 300 mM NaCl, 250 mM Imidazole). The best fractions were pooled together, concentrated, and purified by gel filtration on PD-10 columns (Cytiva).

### Circular dichroism

Circular dichroism (CD) measurements were performed to assess the secondary structure content and folding state of the designed minibinders. Spectra were recorded on a Jasco J-810 spectropolarimeter equipped with a Peltier temperature control unit, using quartz cuvettes with a 1 mm path length. Measurements were carried out at 20 °C. Protein samples were prepared in phosphate buffer (PB), pH 7.4, at a concentration of 20 µM. Far-UV CD spectra were collected over the wavelength range 195–260 nm, with a scan speed of 50 nm min⁻¹, a bandwidth of 1 nm, and a response time of 1 s. For each sample, three consecutive scans were acquired and averaged. Buffer spectra recorded under identical conditions were subtracted from all protein spectra. Raw CD data were smoothed using Tikhonov regularization implemented in in-house code. Spectra were subsequently normalized to mean residue molar ellipticity.

### Surface Plasmon resonance

SPR experiments were performed on a Sierra SPR-32 Pro instrument (Bruker Daltonics GmbH, Bremen, Germany) using CMD50L carboxymethyl-dextran sensor chips (XanTec bioanalytics GmbH, Düsseldorf, Germany). Each flow channel contained four addressable spots, including one blank in-channel reference. Recombinant PDI was immobilized at 20 µg/ml in acetate buffer pH 4.0 on CMD50L surfaces by standard EDC/NHS amine coupling to a typical level of ~5000 RU, followed by deactivation with ethanolamine. Binding measurements were conducted under oxidizing conditions using PBST (PBS + 0.05% Tween-20) supplemented with 2 mM EDTA and 2 mM oxidized glutathione (GSSG) as running buffer. Miniprotein binders (mb6, mb7, and mb12) were prepared in running buffer and injected over immobilized PDI as serial dilutions from 1 µM to 30 µM. Sensorgrams were processed by double referencing, consisting of in-channel reference subtraction and blank buffer injection correction, followed by baseline alignment prior to comparison of binding responses.

### ERO1 and PDI recombinant proteins

Recombinant mouse ERO1A1 (residues 43–464) fused to a GST-SMT3 tag and His-tagged PDIA1 were produced in Rosetta DE3 *Escherichia coli* (Novagen) and purified following previously established protocols [23, 42]. Tags were removed enzymatically, with GST-SMT3 cleavage performed using Ulp protease (Z03691, GenScript) and His-tag removal achieved using thrombin (T6884, Merck). Efficient cleavage was verified by the expected reduction in molecular weight of ERO1 on SDS–PAGE and by loss of anti-His signal in immunoblot analysis of PDI.

### PDI activity assay

PDIA1 enzymatic activity was measured using a turbidimetric insulin reduction assay. Bovine insulin (1 mg/ml) was prepared in sodium phosphate buffer (pH 7.0) supplemented with 2 mM EDTA and incubated with 1.4 μM recombinant PDIA1 in the presence of vehicle control (1% DMSO) or increasing concentrations of minibinders for 15 minutes. Insulin disulfide bond reduction was catalyzed by PDI previously reduced with dithiothreitol (DTT) and purified by the DTT excess with PD10 columns. Changes in solution turbidity were recorded at 650 nm over 80 minutes.

### Reagents

Bovine insulin, EDTA, and dithiothreitol were purchased from Sigma-Aldrich. EN460 was synthesized in house accordingly to refs. [23, 24].

### AUR-based kinetic assay of ERO1 PDI activity

The kinetic assay of ERO1A activity is based on a reaction involving recombinant ERO1A and PDIA1 proteins, horseradish peroxidase (Worthington), and 5 μM Amplex Ultra Red, as detailed in [23]. The effect of the minibinder was evaluated by measuring the inhibition of the linear reaction rate, and the corresponding IC₅₀ values were determined using Prism 10 (GraphPad).

### Liposome-containing minibinder preparation

Minibinders were encapsulated into liposomes using the freeze–thaw method [43]. Briefly, cholesterol (Sigma-Aldrich, C8667) and L-α-Phosphatidylcholine (PC) (Sigma-Aldrich, cod.61755) were dissolved in chloroform at a 1:1 molar ratio and deposited as a thin lipid film in a round-bottom glass tube by evaporation of the organic solvent under a gentle nitrogen stream. Residual solvent was removed by placing the lipid film in a vacuum desiccator for 30 min. The dried lipid film was then rehydrated by exposure to water vapor for 30 min, after which 500 μL of minibinder solution was added directly onto the lipid film. The lipid suspension was rapidly frozen by immersion in liquid nitrogen, followed by thawing in a 40 °C water bath. This freeze–thaw cycle was repeated several times to promote efficient encapsulation of the minibinders. Following freeze–thawing, liposomes were pelleted by ultracentrifugation at 4 °C for 35 min at 40,000 rpm. The supernatant containing non-encapsulated minibinders was discarded, and the liposome pellet was resuspended in phosphate-buffered saline (PBS, pH 7.4) to obtain a final minibinder concentration of 150 μM.

### Analysis of minibinders encapsulated in liposomes

After liposome preparation, the concentration of minibinders encapsulated in the liposomal solution was determined using a MALDI–TOF mass spectrometer. Liposomes were lysed by sonication for five minutes, and an aliquot was directly analyzed by mass spectrometry together with the corresponding stock solution used for liposome preparation and the supernatant obtained after liposome centrifugation. MALDI–TOF mass spectrometry (4800 MALDI–TOF, Applied Biosystems) was performed in linear positive ion mode. Samples were mixed with the matrix (sinapinic acid, Sigma-Aldrich), spotted onto the MALDI target plate, and analyzed over a molecular weight range of 4000–12,000 Da.

### Cancer cell line

E0771 and LMM3 breast cancer cells were kept in culture for no more than two weeks and routinely tested for mycoplasma infection.

### MTS

E0771 and LMM3 cells were plated at a density of 7500 cells per well in 96-well plates and maintained in culture medium supplemented with 10% fetal bovine serum (FBS). Cells were exposed to increasing concentrations of liposome-containing minibinder or empty liposome, resuspended in serum-free culture medium for 24 hours. Then, culture medium supplemented with 10% FBS was added. After other 48 hours cell viability was assessed using the MTS/PMS-based colorimetric assay according to the manufacturer’s protocol (CellTiter 96® Aqueous Non-Radioactive Cell Proliferation Assay, Promega). Absorbance was recorded at 490 nm using a TECAN Infinite M200 microplate reader.

### Immunofluorescence analysis of mb7 in E0771 cells

Immunofluorescence analyses of His-tagged mb7 in E0771 cells were performed using an Olympus IX71 fluorescence microscope. Briefly, E0771 cells were fixed, permeabilized, and incubated with a mouse anti-His tag primary antibody (clone AD1.1.10, 1:100; R&D Systems) to detect intracellular mb7, together with either a rabbit polyclonal anti-ERO1A antibody (1:50; homemade antibody raised against recombinant ERO1A protein) or a mouse monoclonal anti-PDI antibody (clone 1D3, ADI-SPA-891, 1:100; Enzo Life Sciences). After incubation with the appropriate fluorescent secondary antibodies, nuclei were counterstained with DAPI, and randomly selected fields were imaged. His-tagged mb7 was visualized in the green channel, whereas ERO1A or PDI staining was detected in the red channel, while nuclei were visualized by DAPI staining.

### Statistics

Data were analyzed using GraphPad Prism 10 (GraphPad Software). The statistical tests used, as well as sample sizes (N), are indicated in the figure legends. Comparisons between two normally distributed groups were performed using an unpaired, two-tailed Student’s *t*-test. For grouped analyses, two-way ANOVA with Sidak’s correction for multiple comparisons was applied. Results are presented as mean ± SEM or mean ± SD, as specified in the figure legends. A *P* value < 0.05 was considered statistically significant.

## Supplementary information

Supplementary

## Data Availability

The datasets generated during the current study are available from the corresponding author on reasonable request.
