# Supplementary material for: Targeting the ERO1A–PDIA1 redox axis in triple-negative breast cancer with AI-designed minibinders
Source: Cell Death Discov. 2026 Aug 10;12:444. doi: 10.1038/s41420-026-03301-w (PMC13740268; doi:10.1038/s41420-026-03301-w)

Supplementary Table of Contents

1. Supplementary Figure Legends and Figures

Figure Supplementary 1 linked to Figure 2

A tissue microarray (TMA) containing breast cancer samples, including 48 classified as Luminal A and 52 as TNBC, was analyzed by immunofluorescence for PDI, with nuclei counterstained with DAPI. Immunofluorescence staining for all tumor samples is shown.

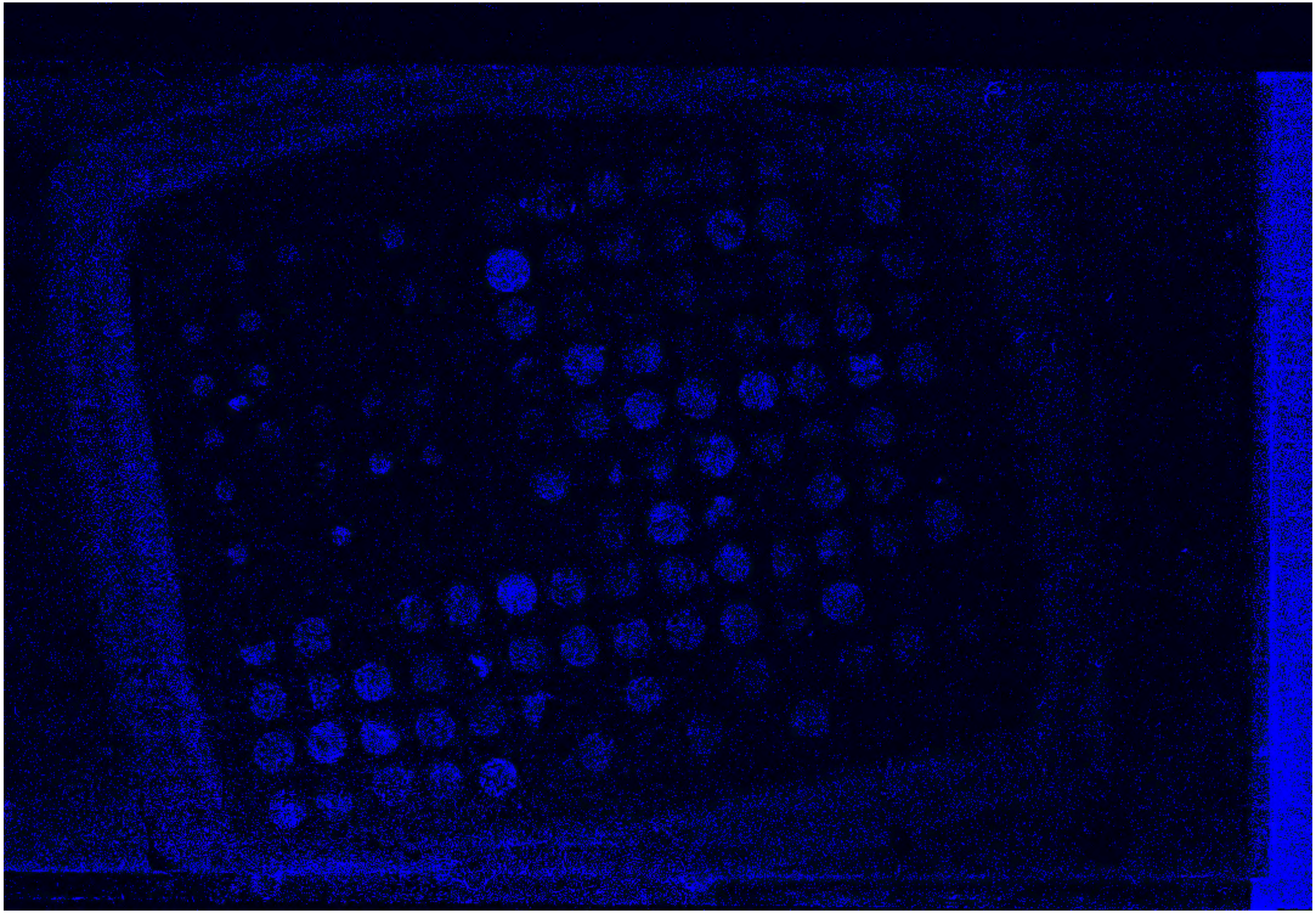

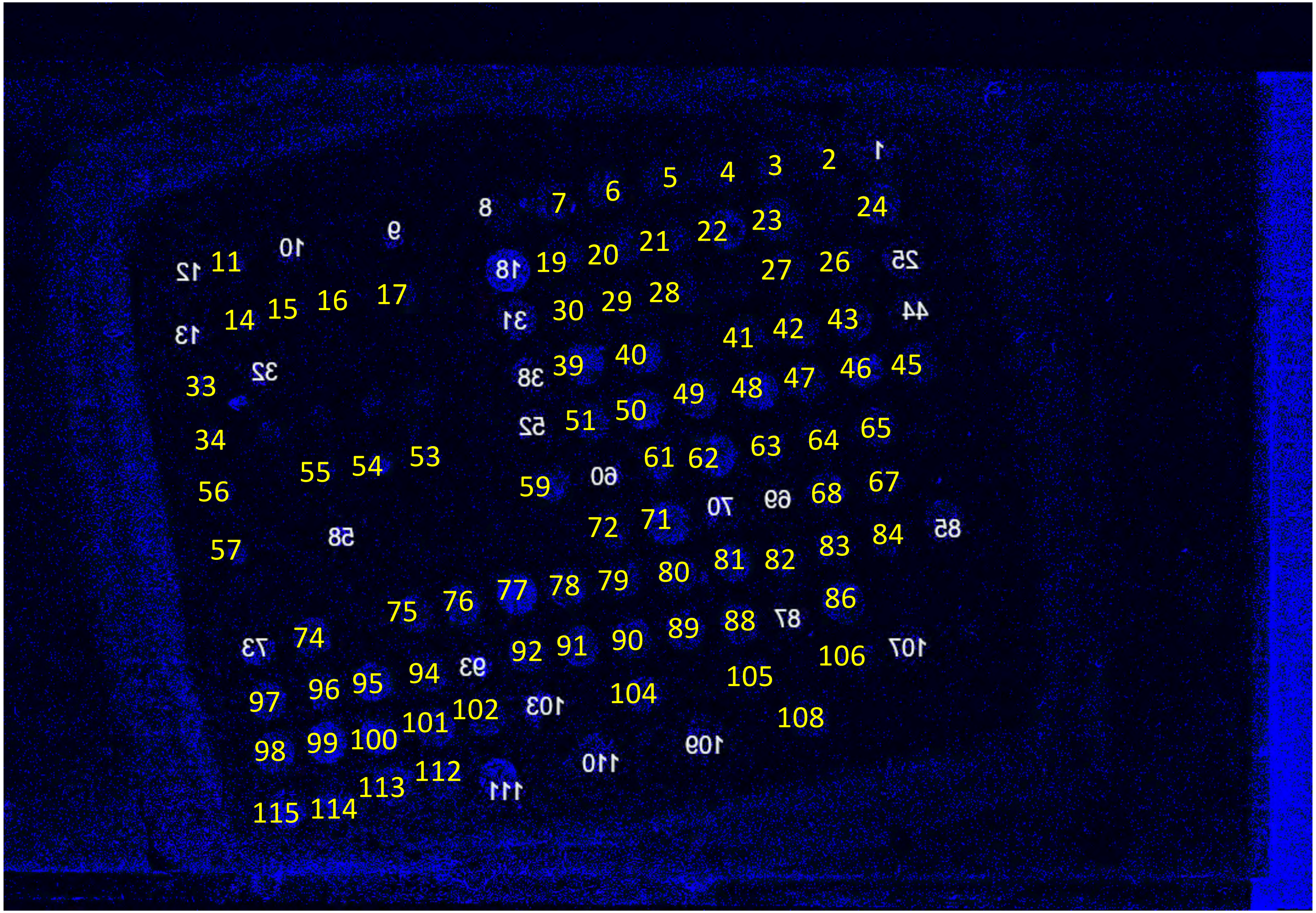

1
2
3
4
5
6
7
8
9
10
11
12
13
14
15
16
17
18
19
20
21
22
23
24
25
26
27
28
29
30
31
32
33
34
38
39
40
41
42
43
44
45
46
47
48
49
50
51
52
53
54
55
56
57
58
59
60
61
62
63
64
65
67
68
69
70
71
72
73
74
75
76
77
78
79
80
81
82
83
84
85
86
87
88
89
90
91
92
93
94
95
96
97
98
99
100
101
102
103
104
105
106
107
108
109
110
111
112
113
114
115

1

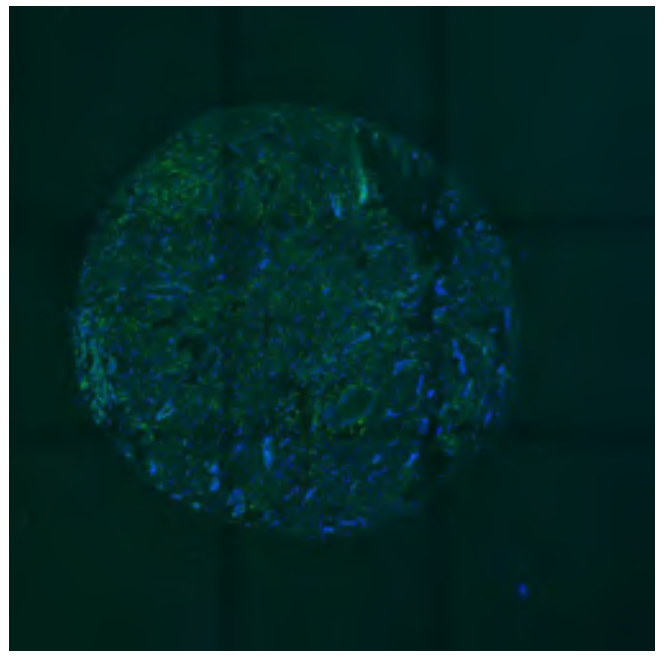

2

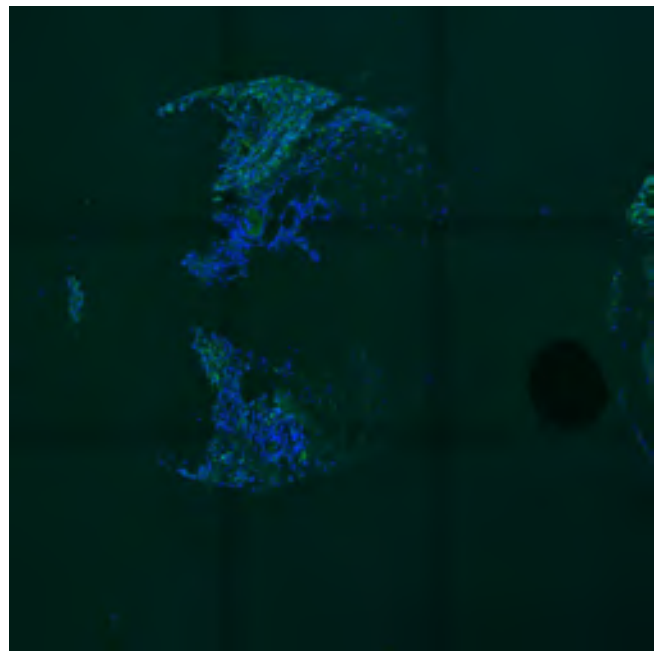

3

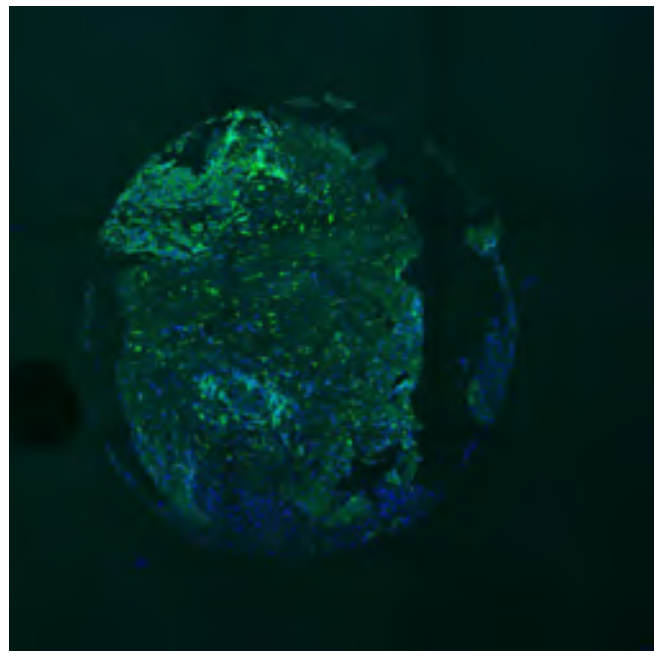

4

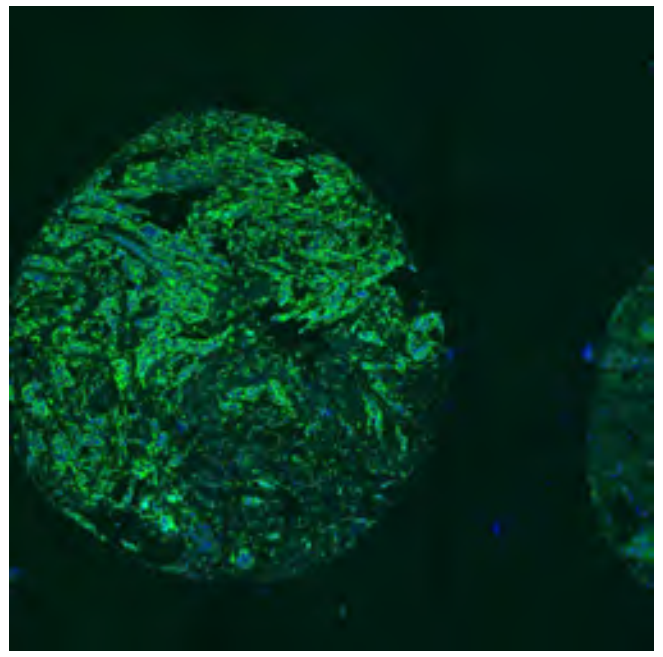

5

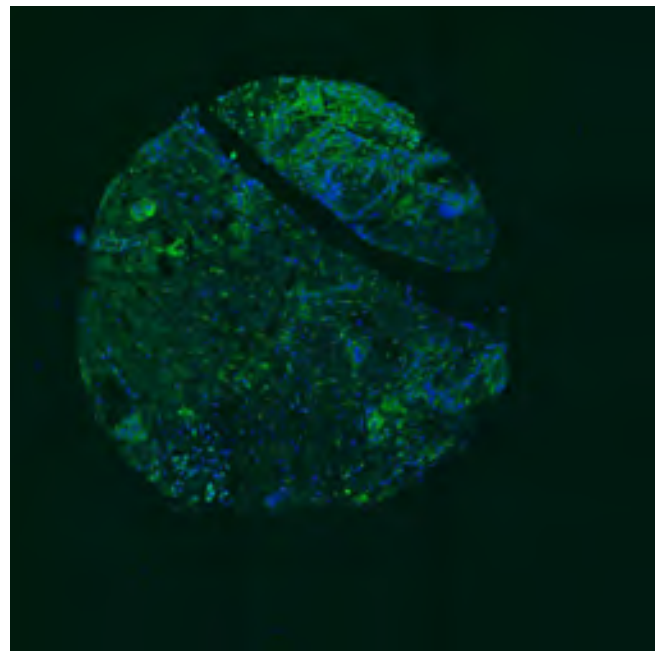

6

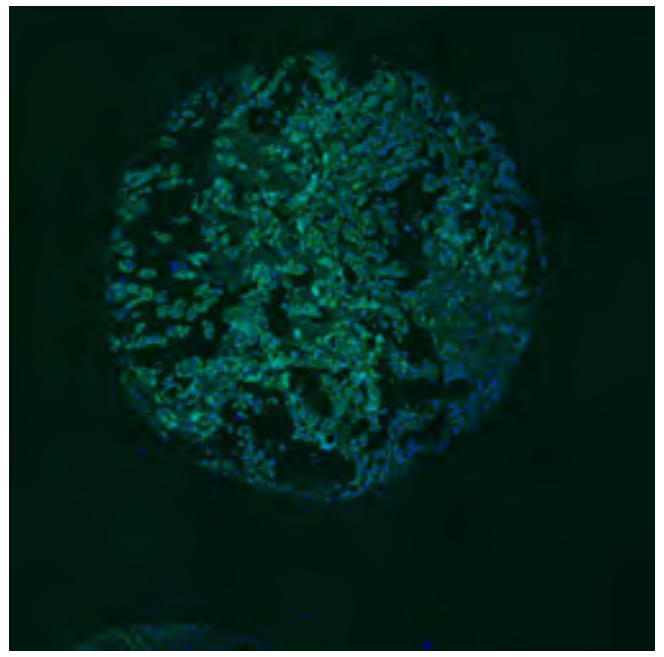

7

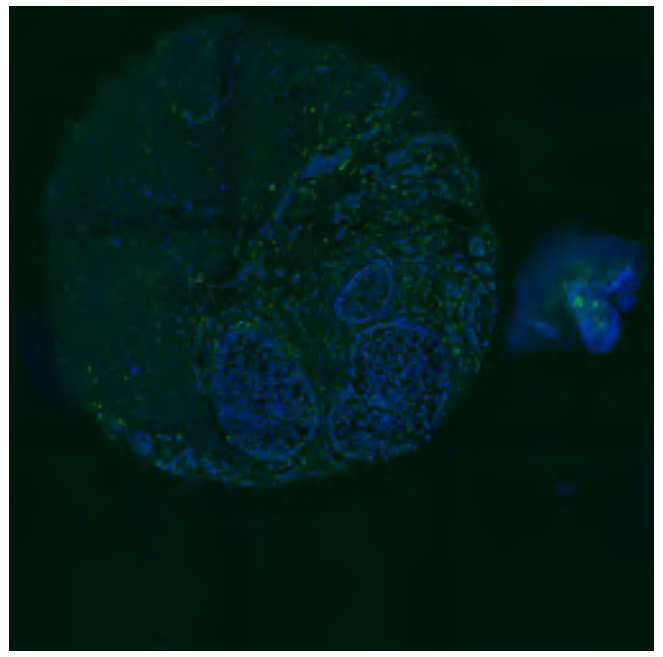

8

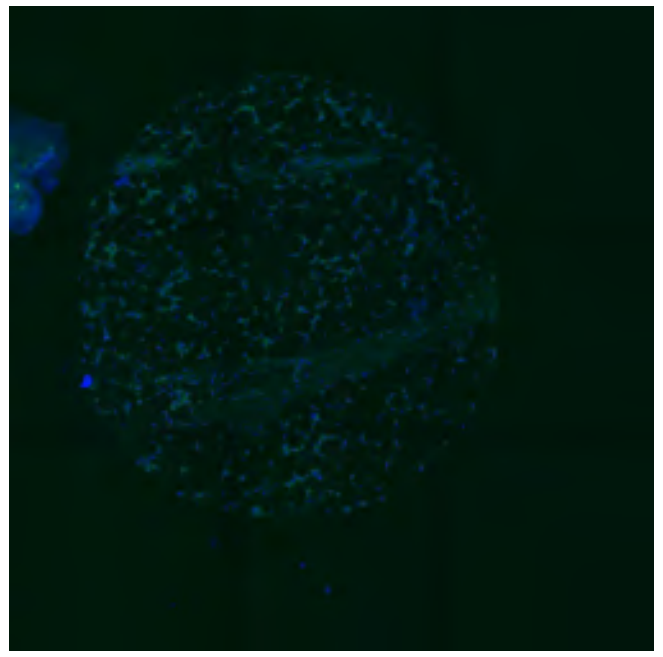

9

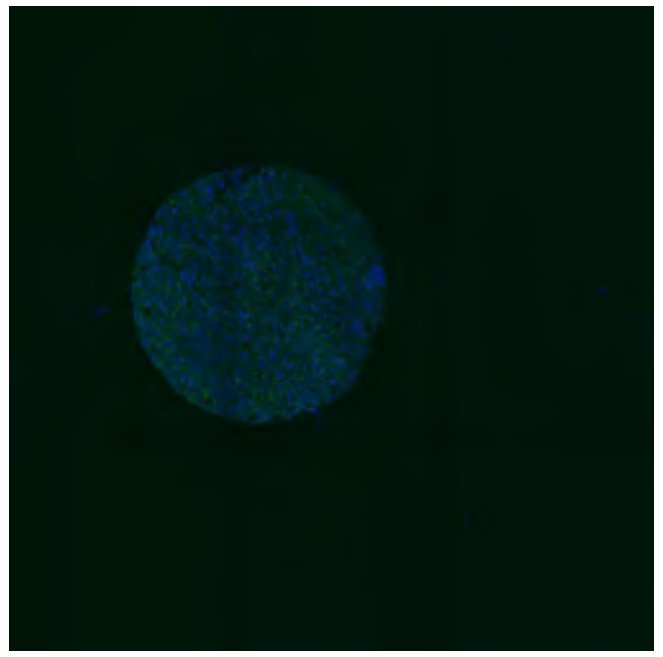

10

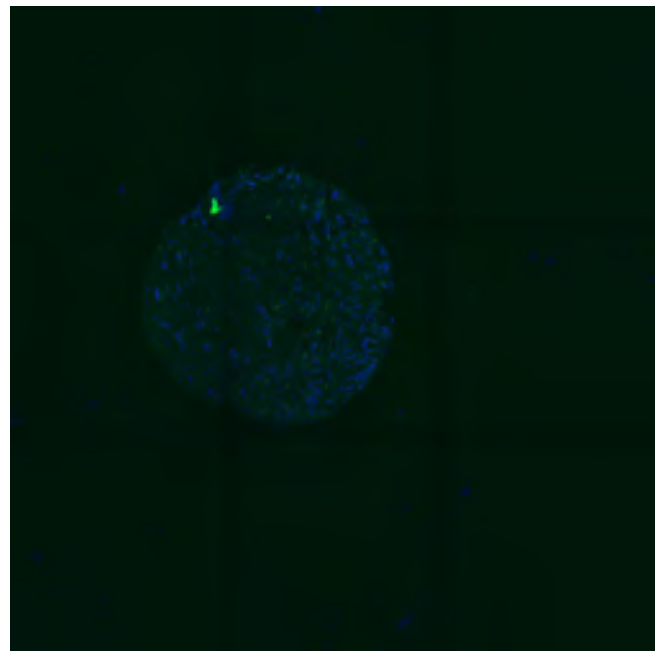

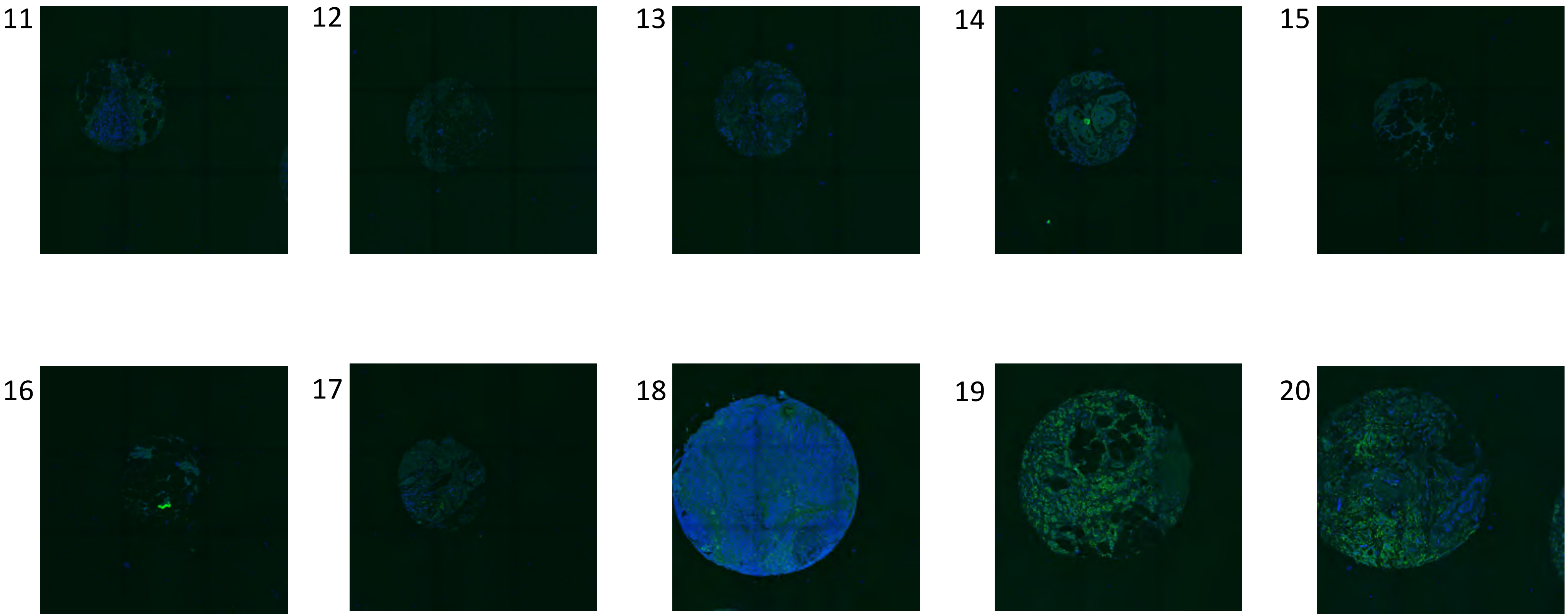
11
12
13
14
15
16
17
18
19
20

21

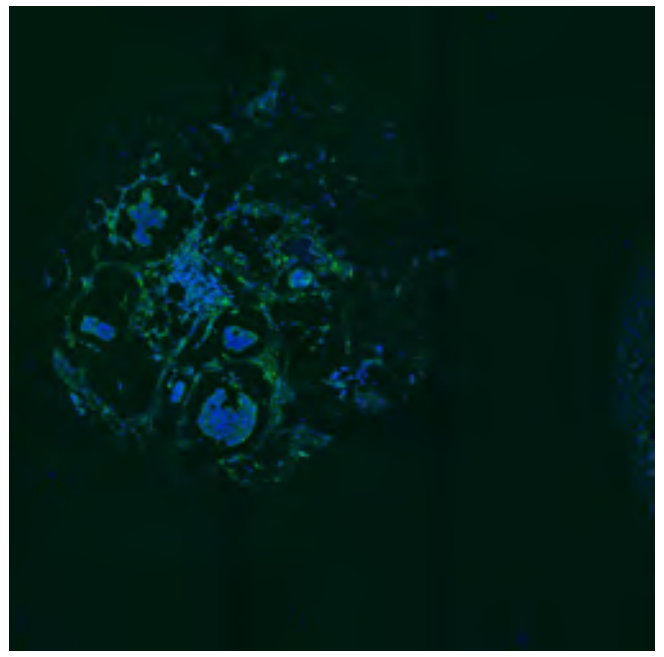

22

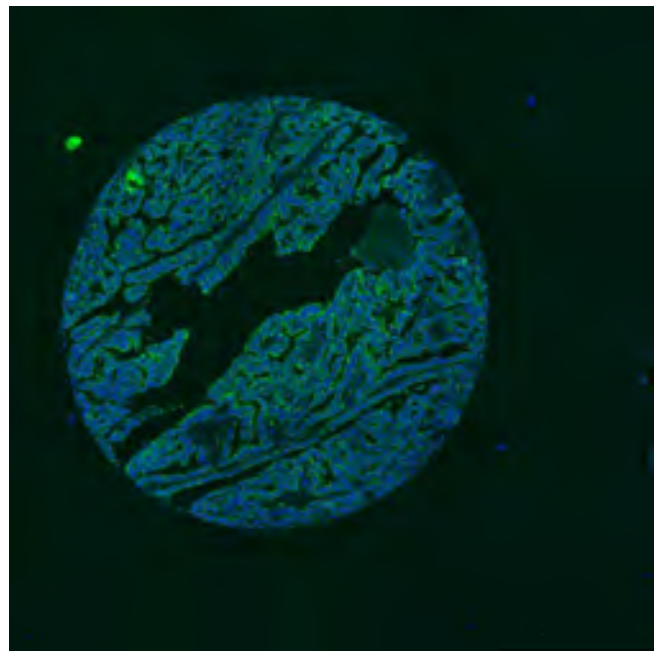

23

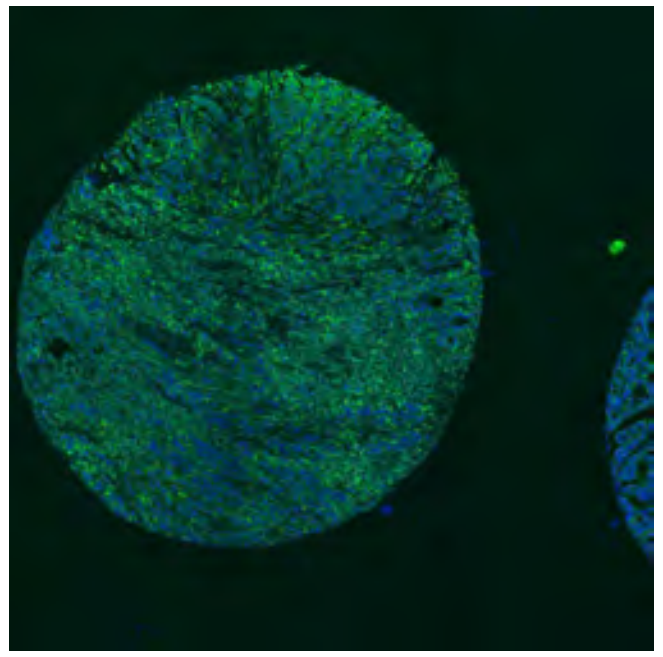

24

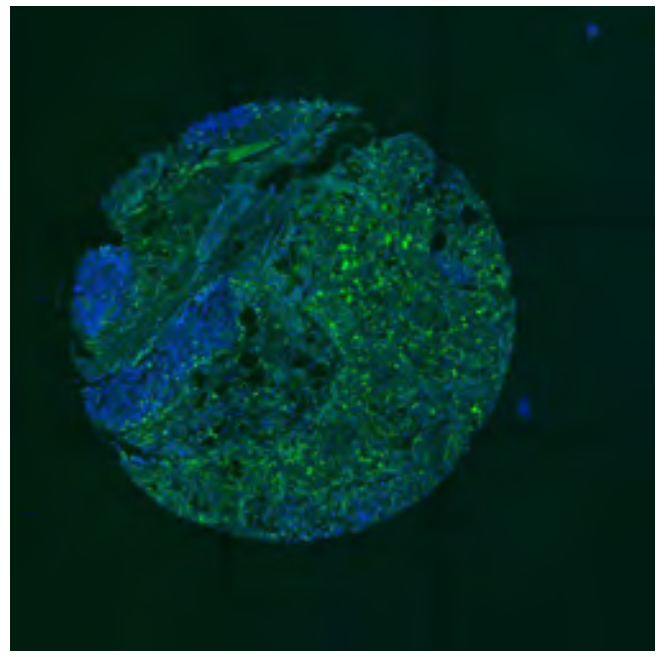

25

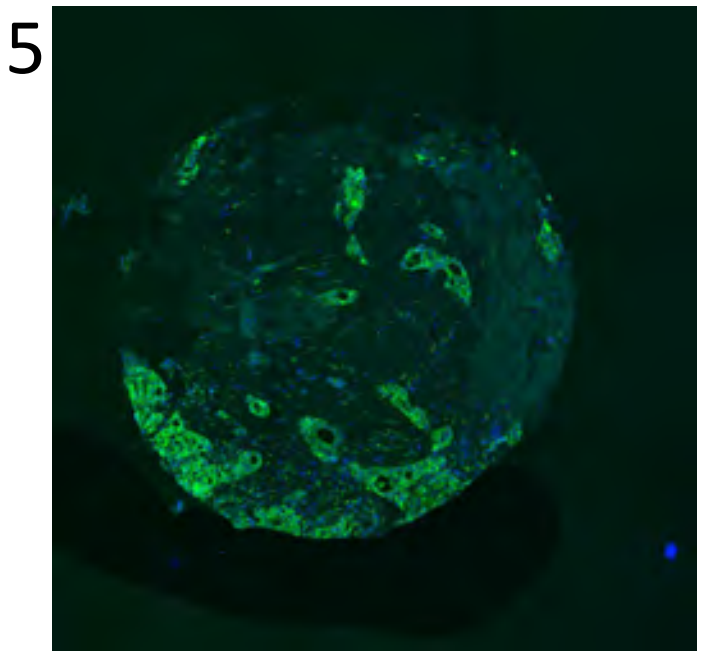

26

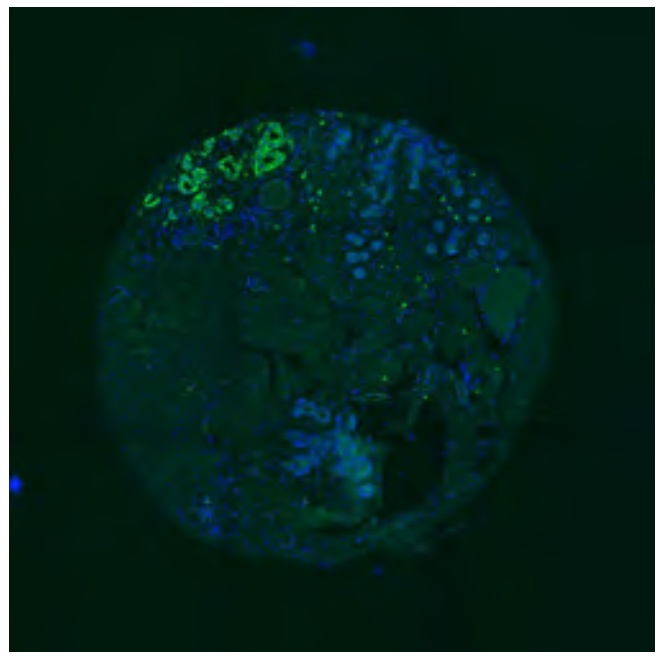

27

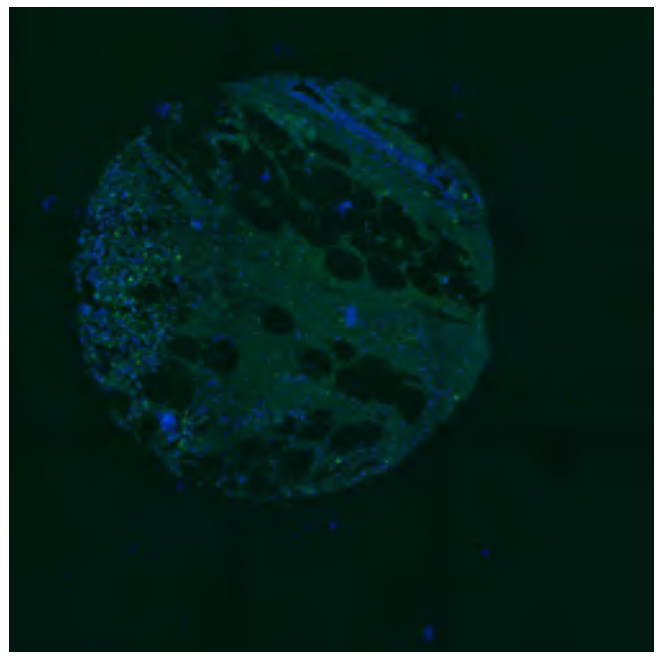

28

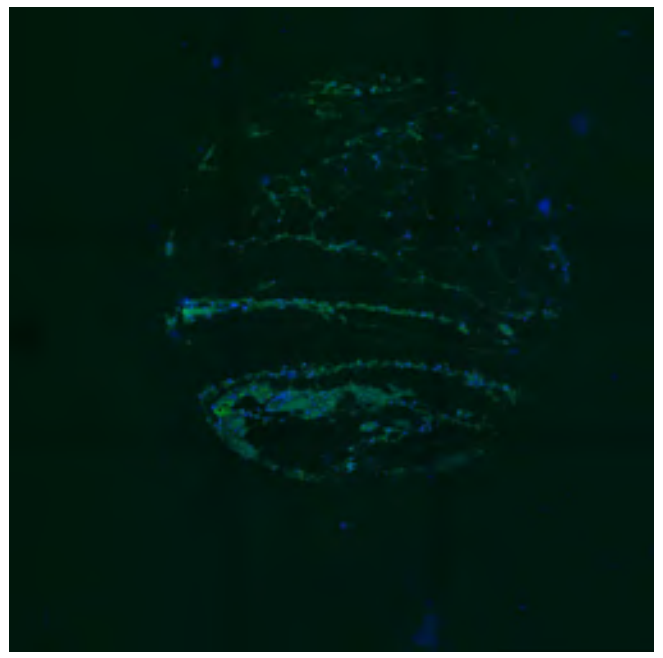

29

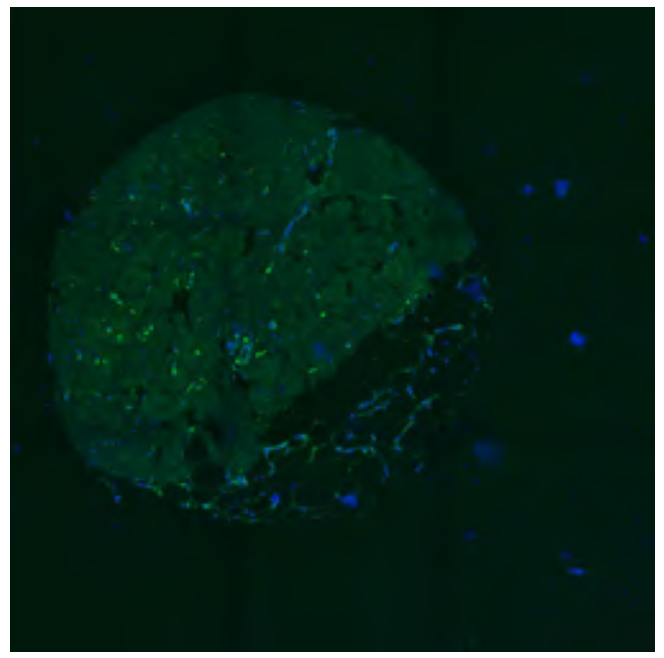

30

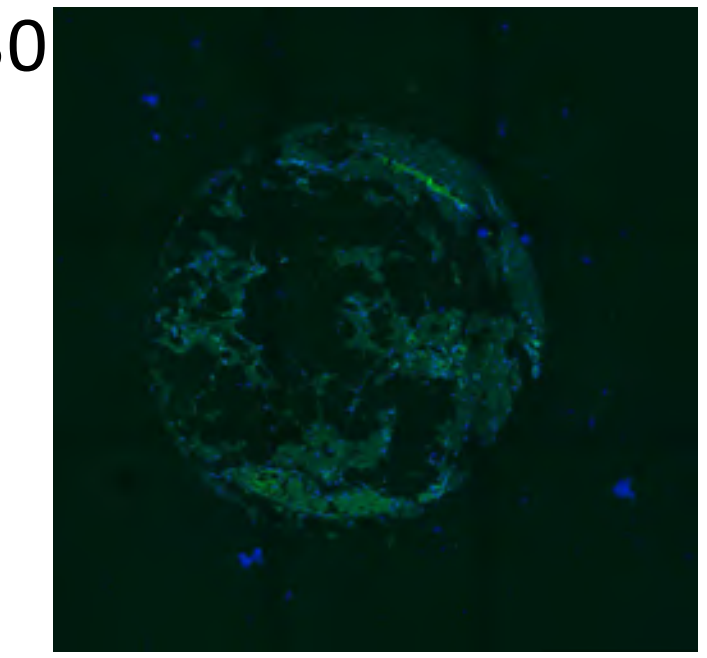

31
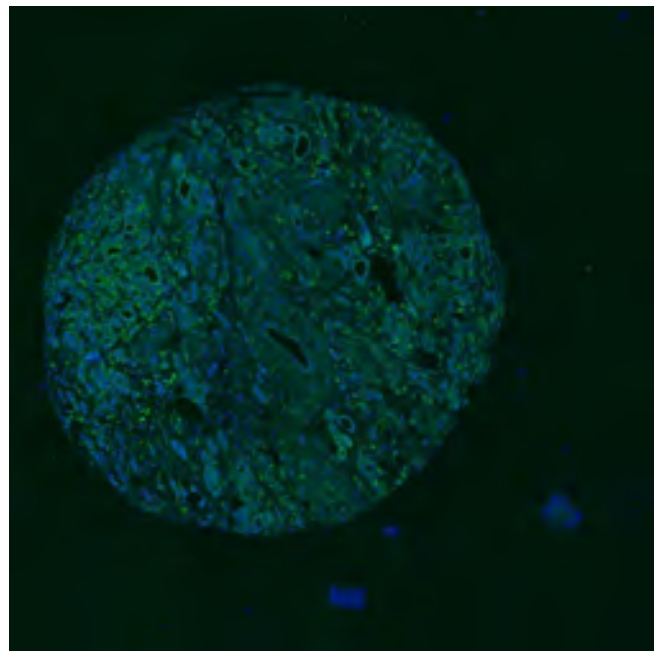
32
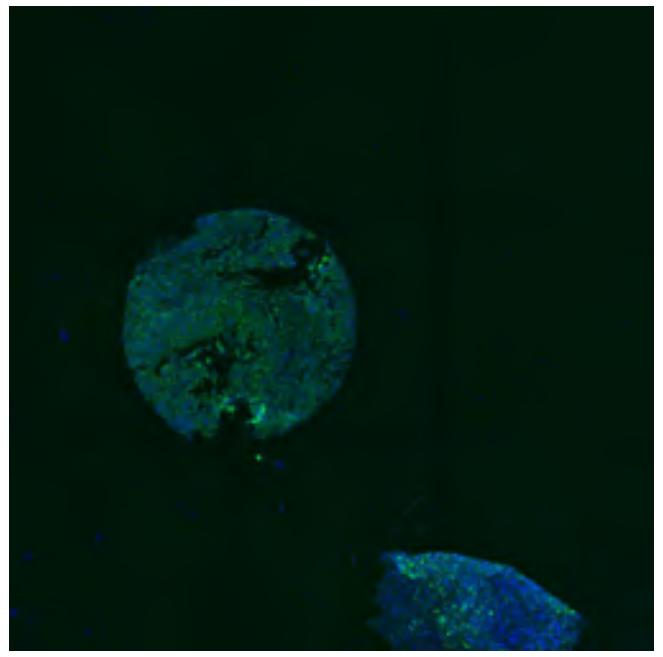
33
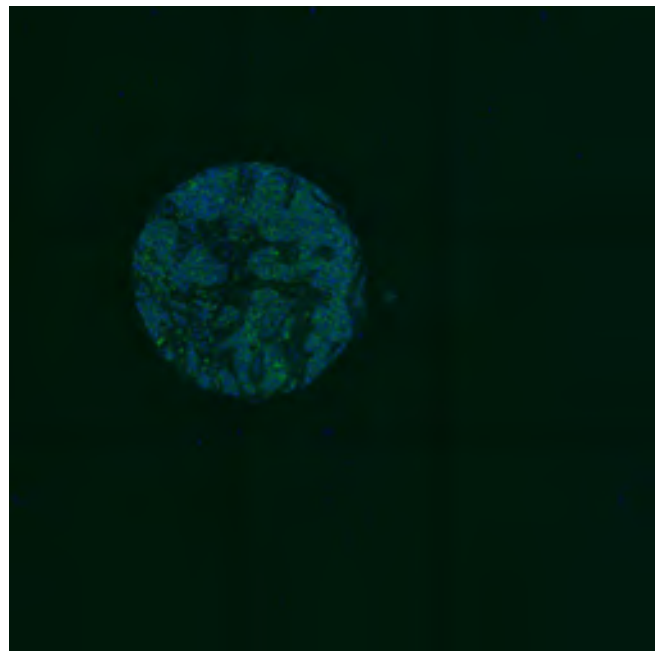
34
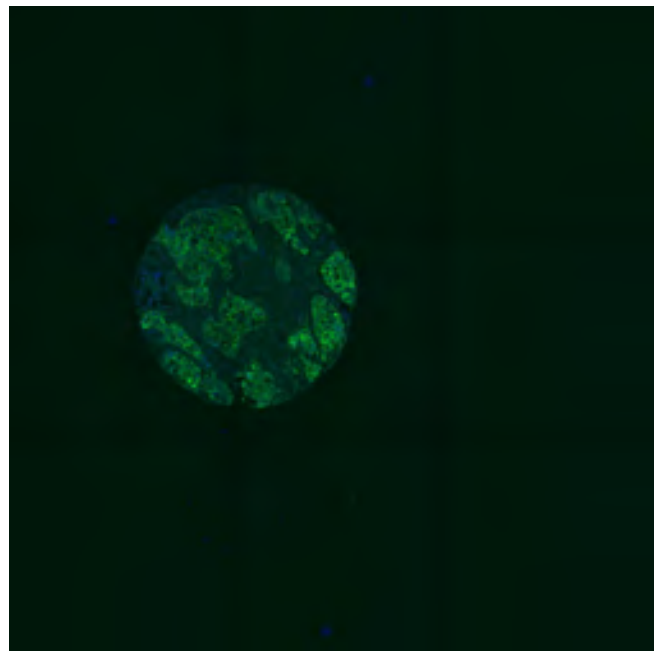
35
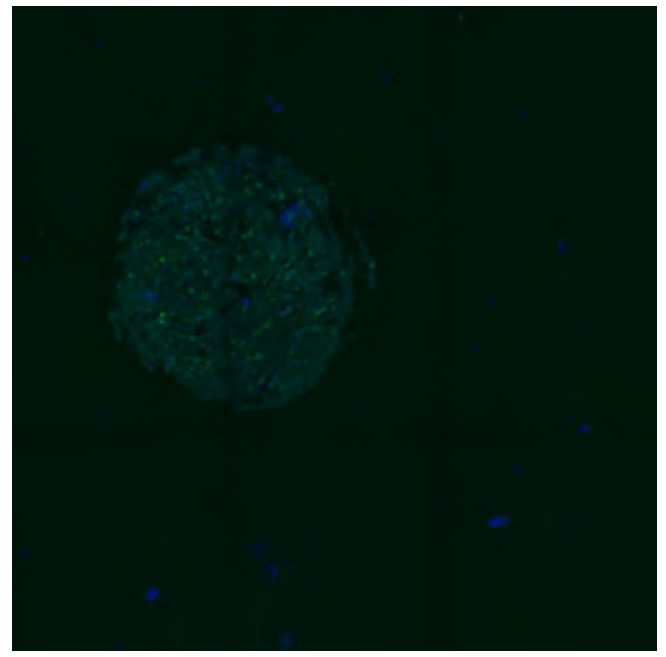
36
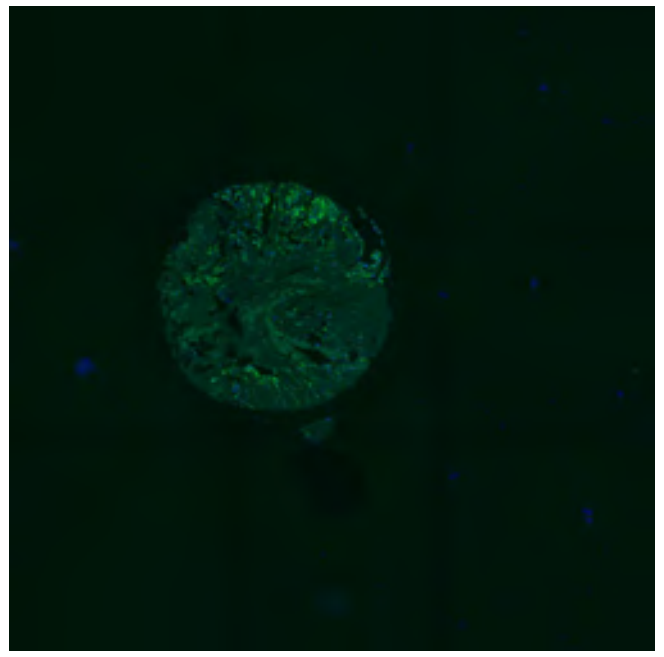
37
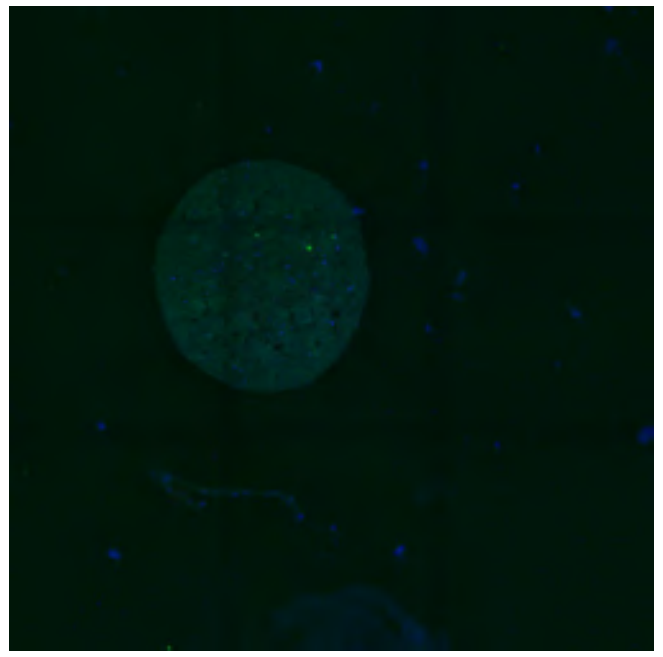
38
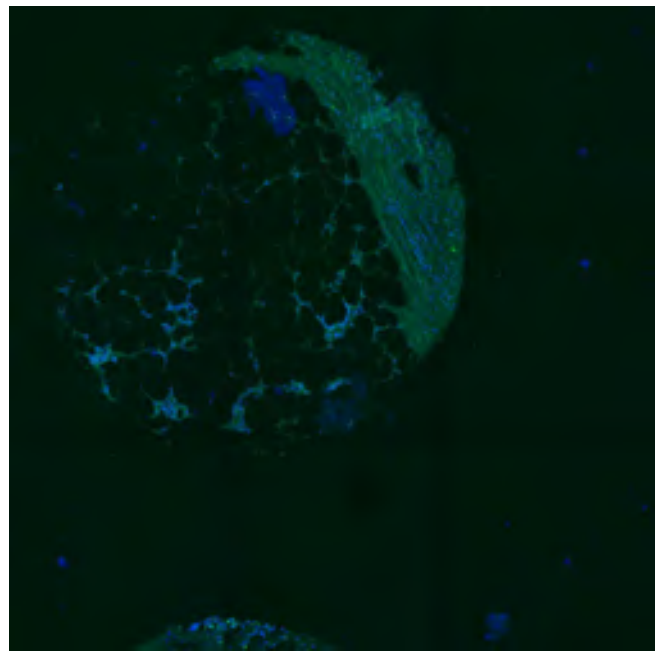
39
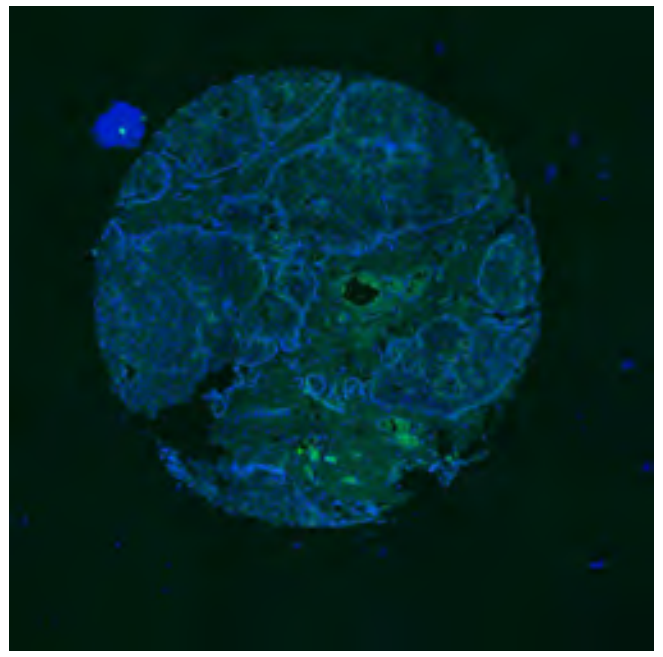
40
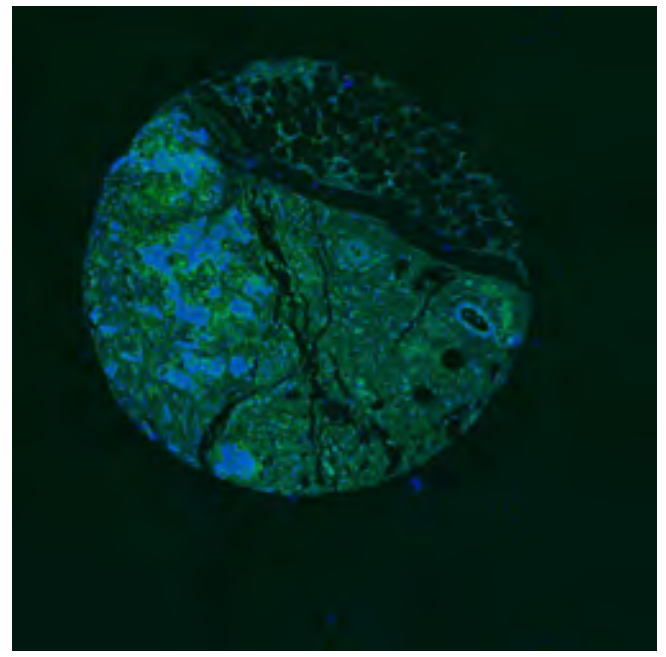

41

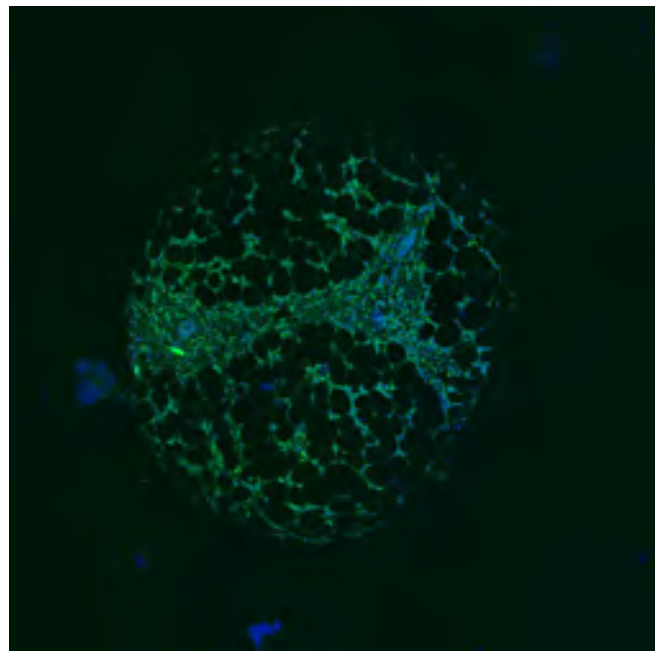

42

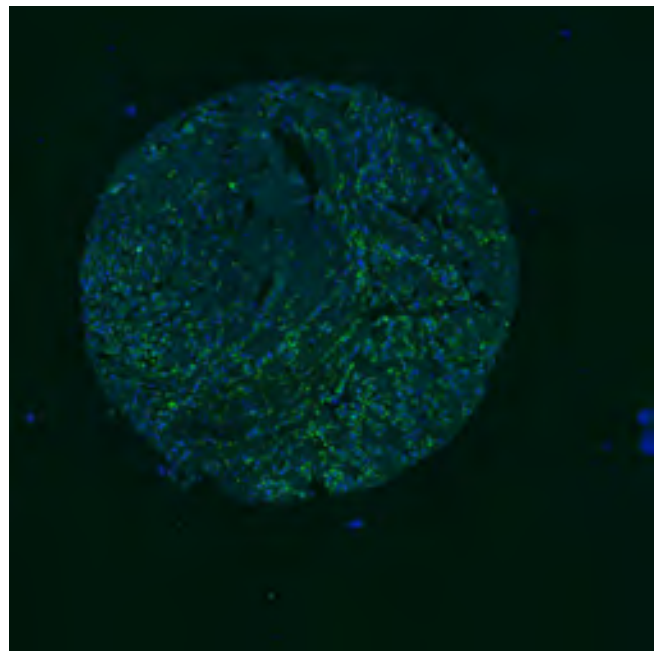

43

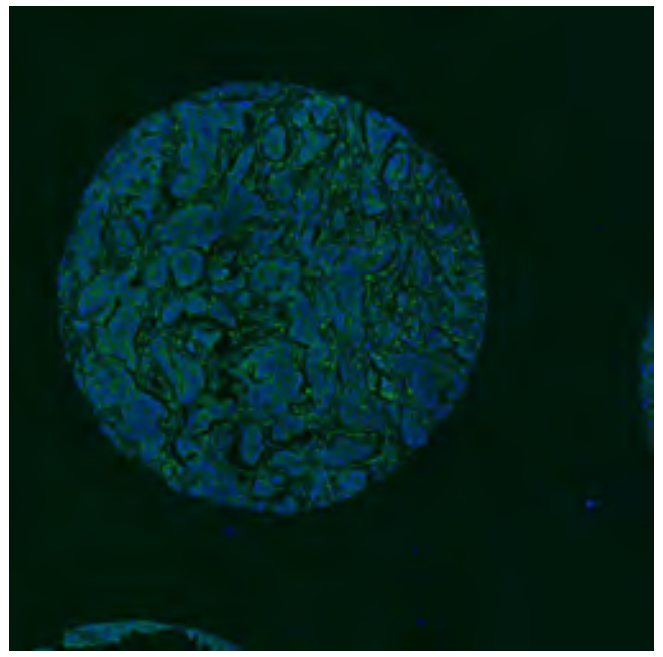

44

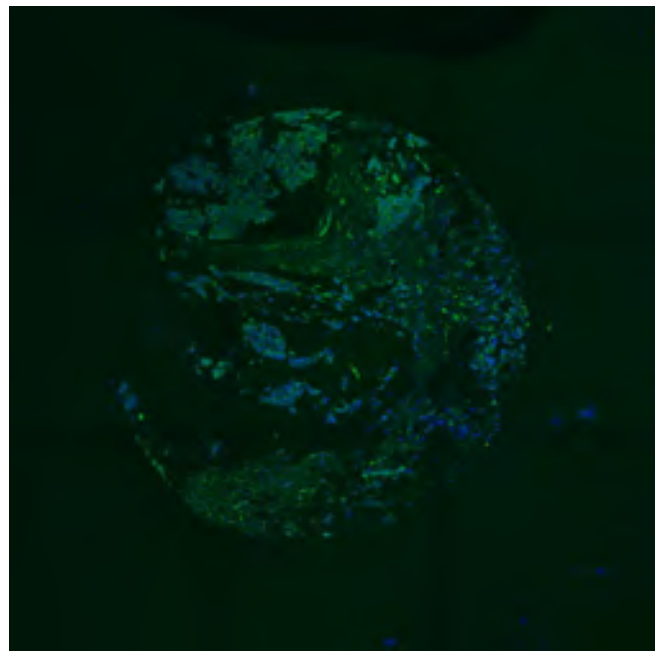

45

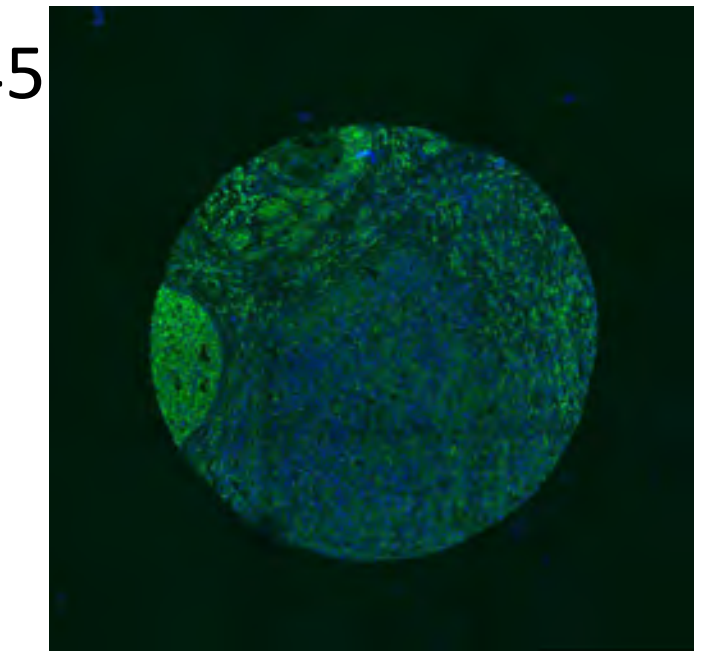

46

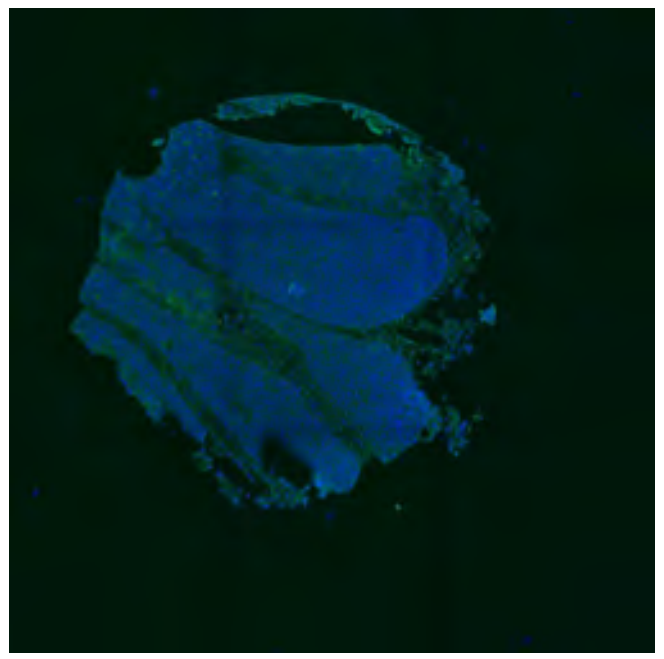

47

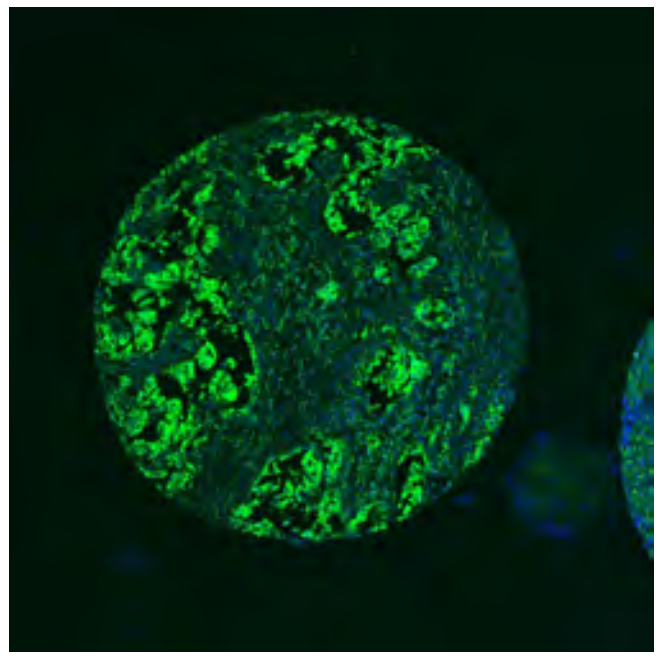

48

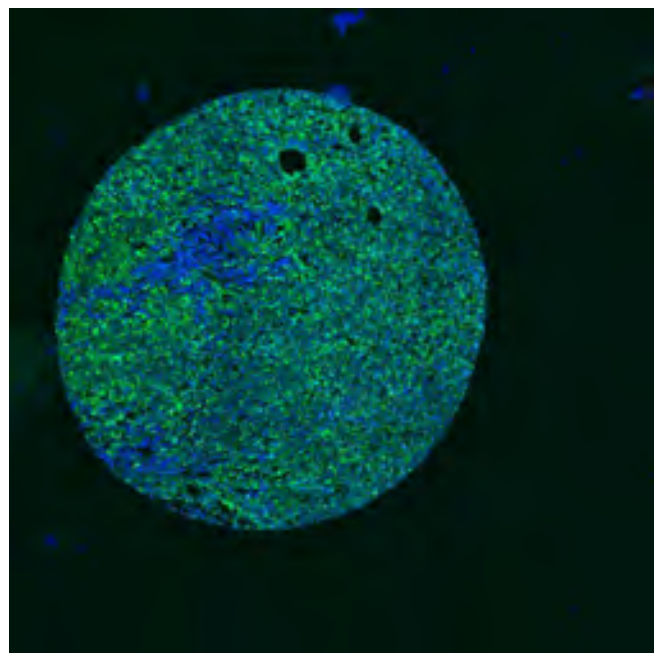

49

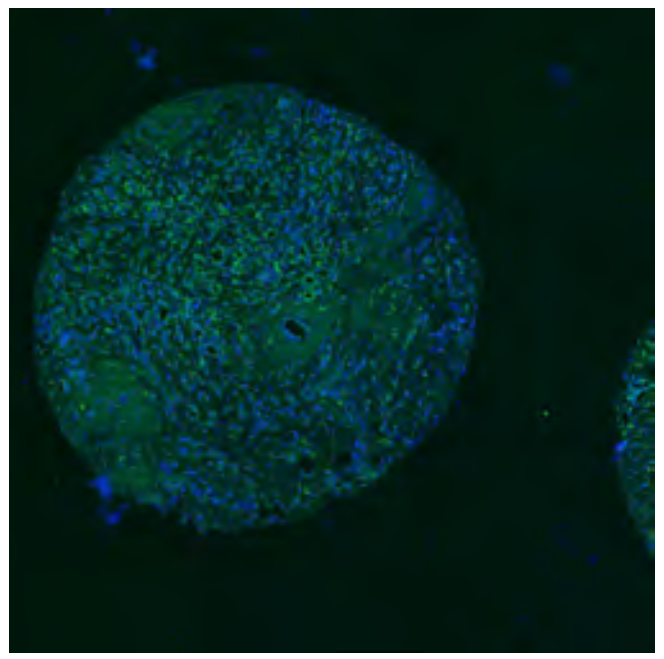

50

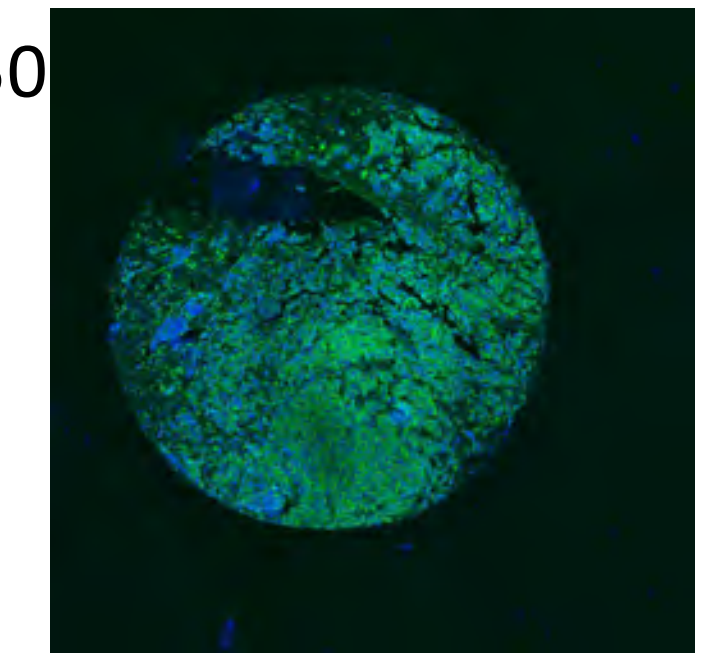

51

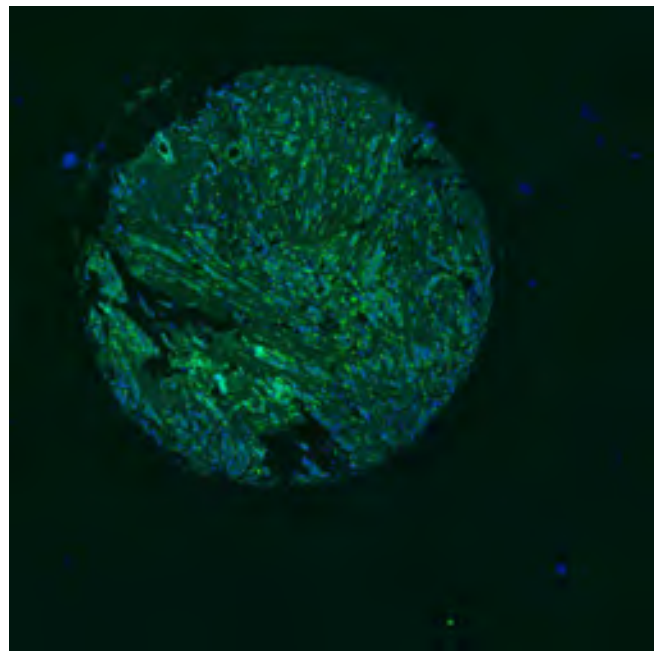

52

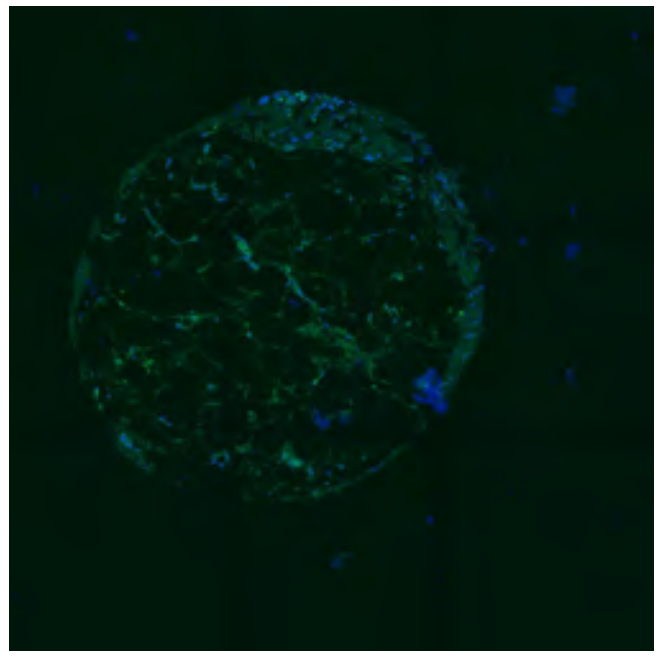

53

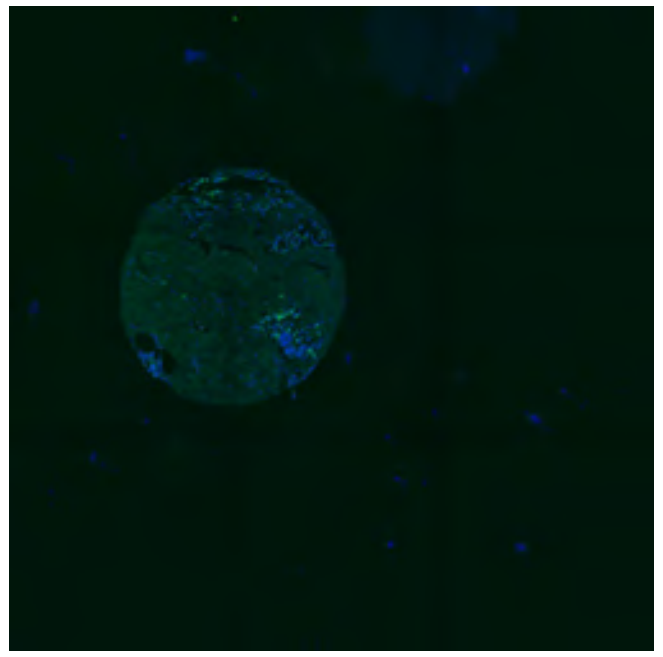

54

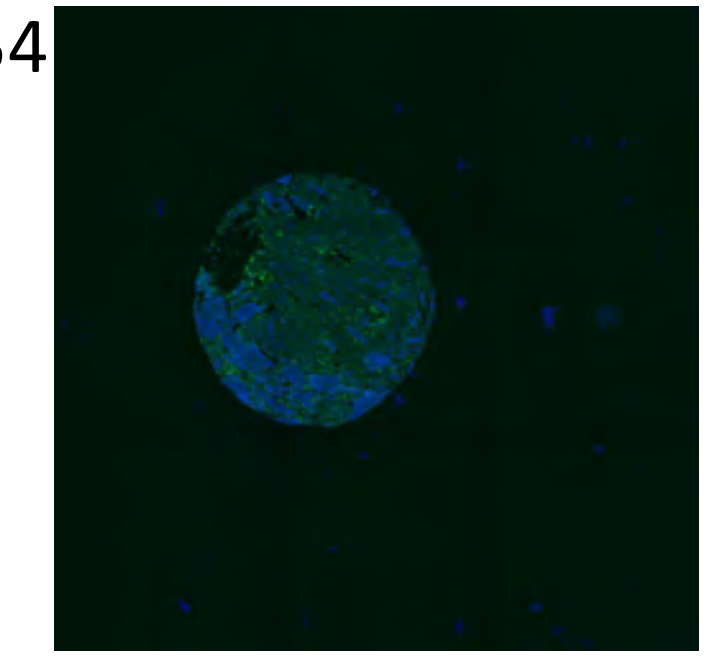

55

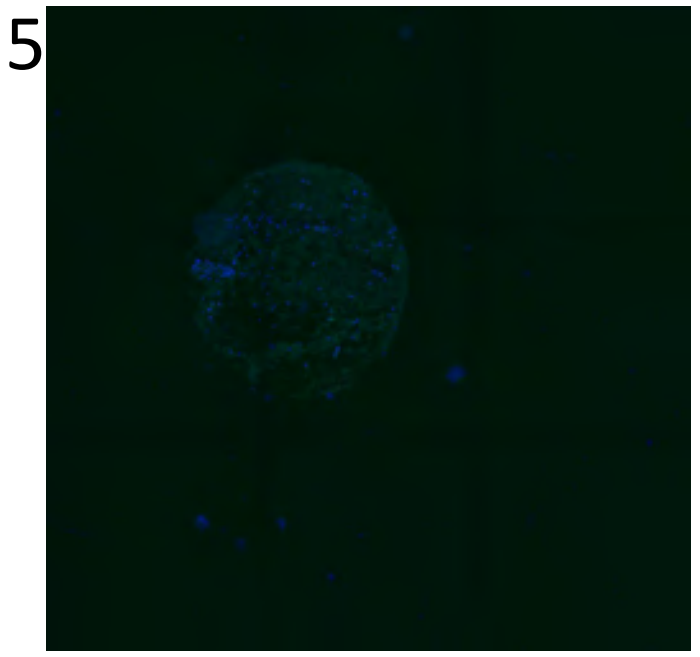

56

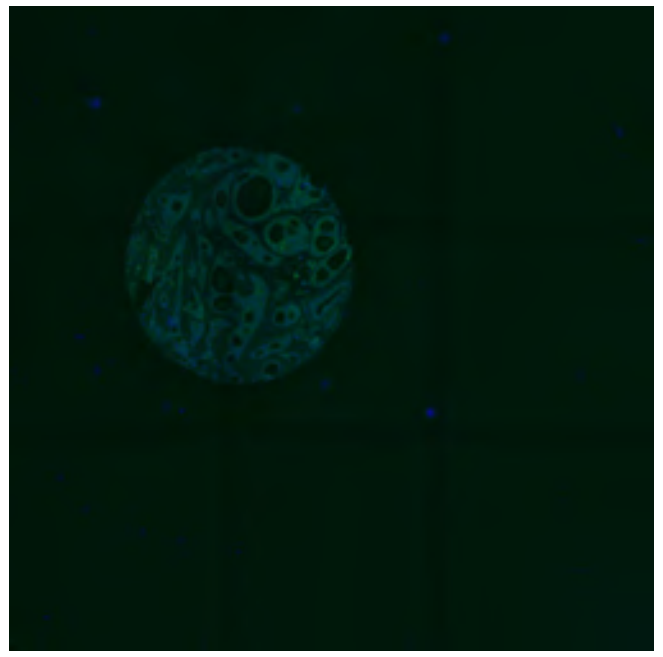

57

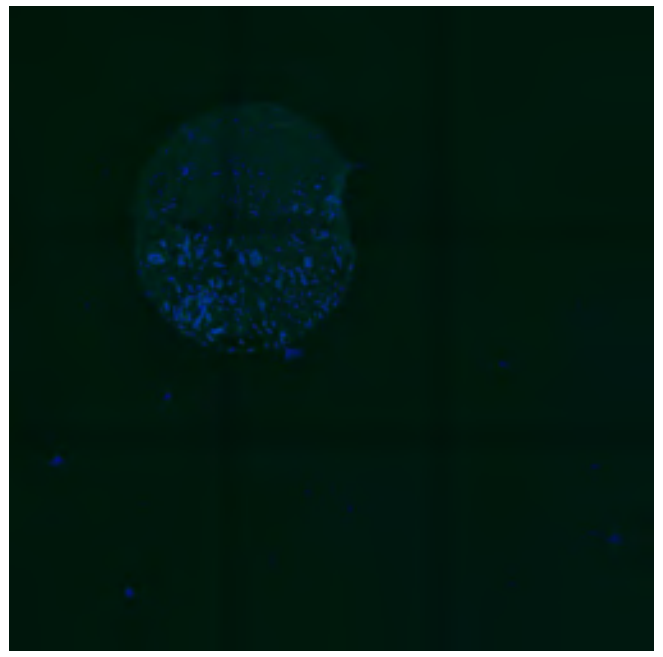

58

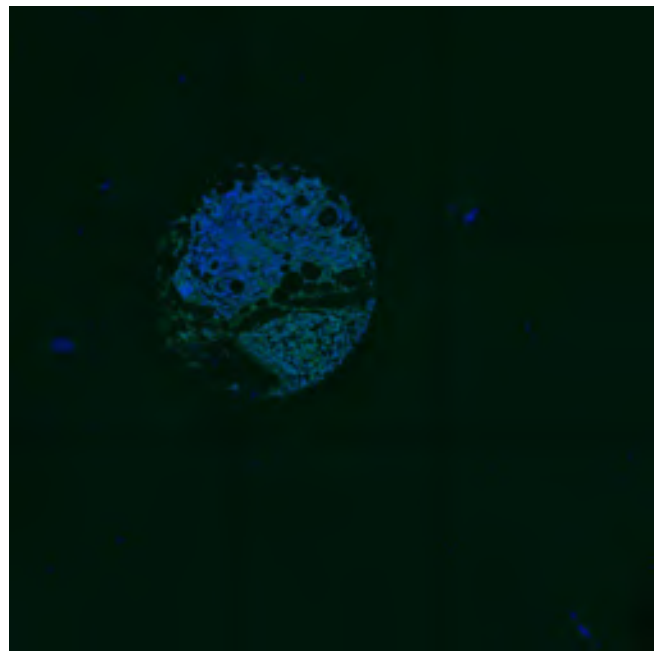

59

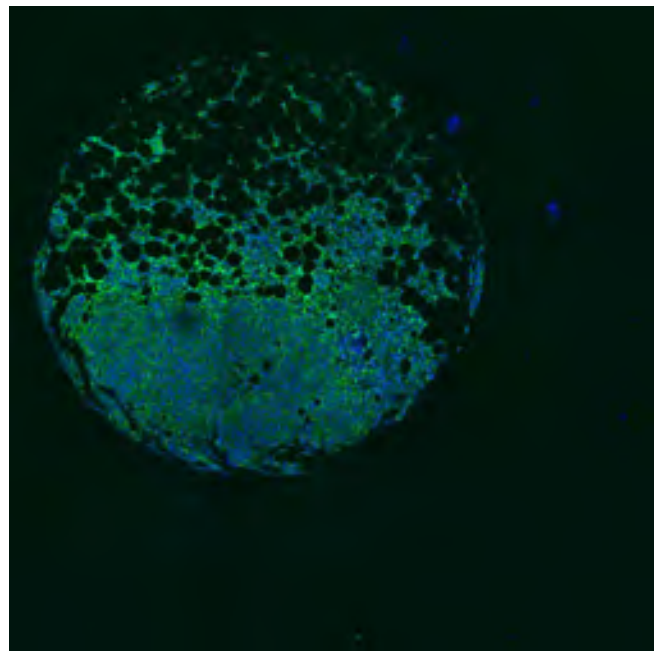

60

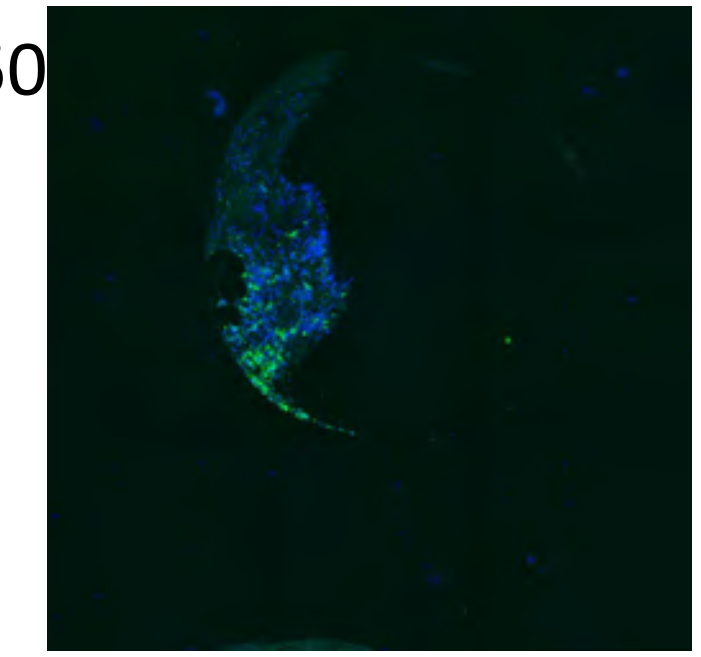

61
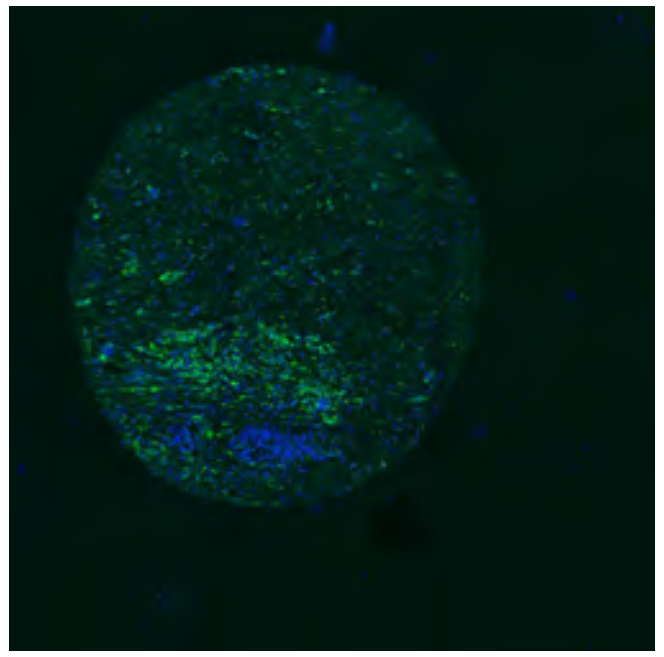

62
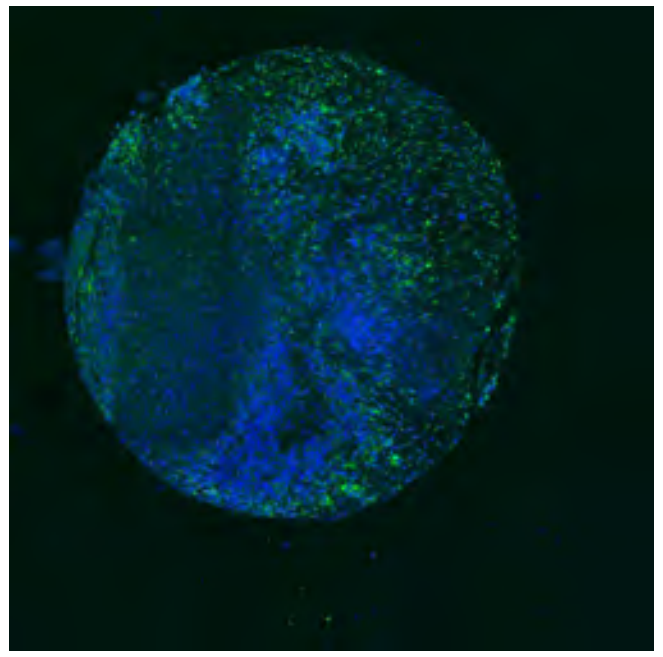

63
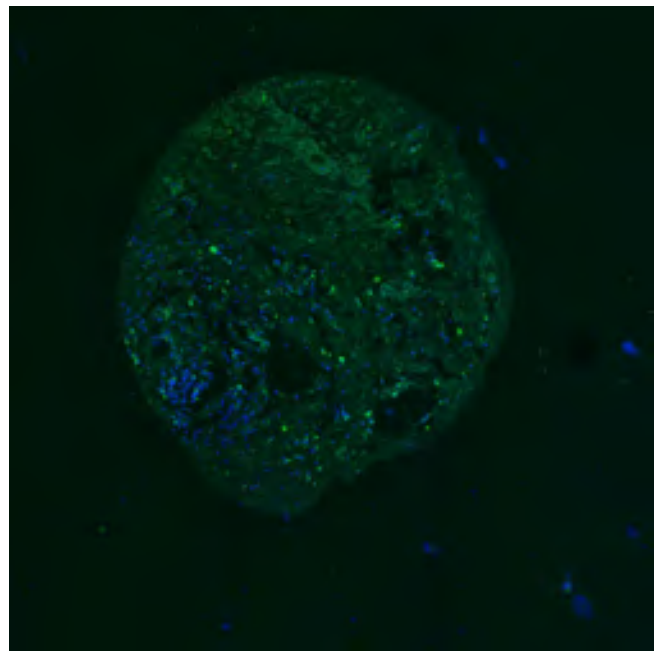

64
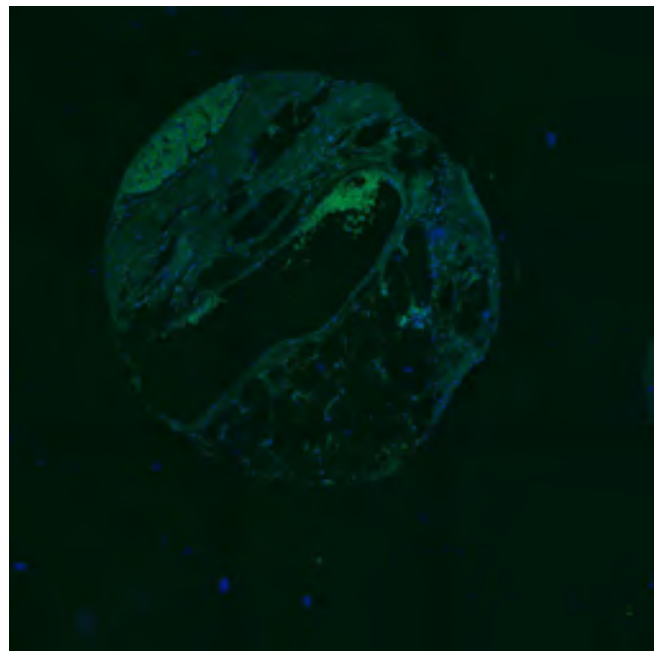

65
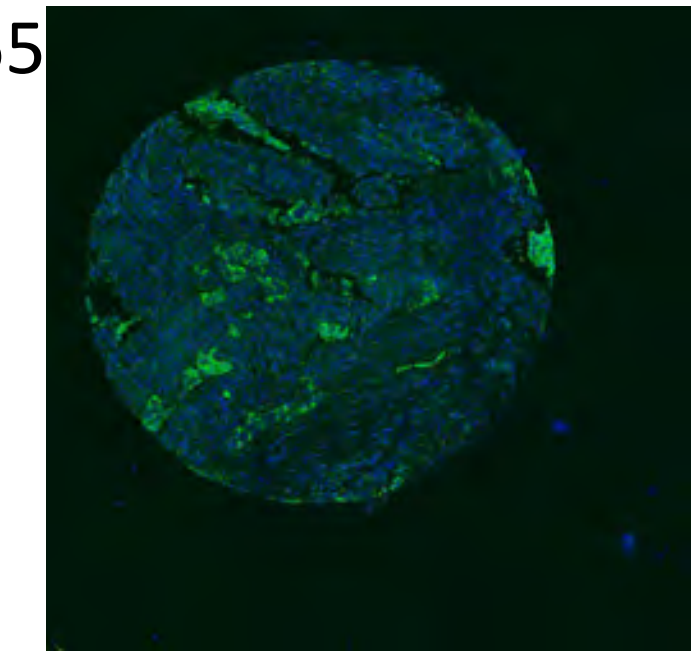

66
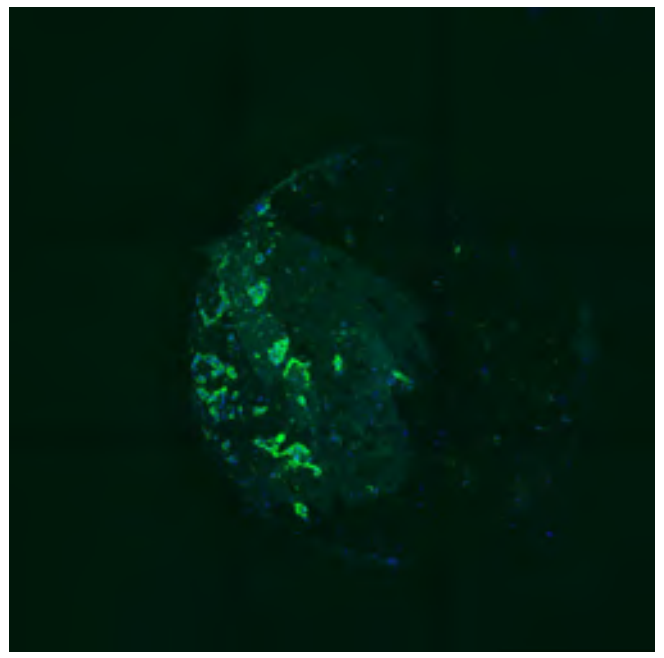

67
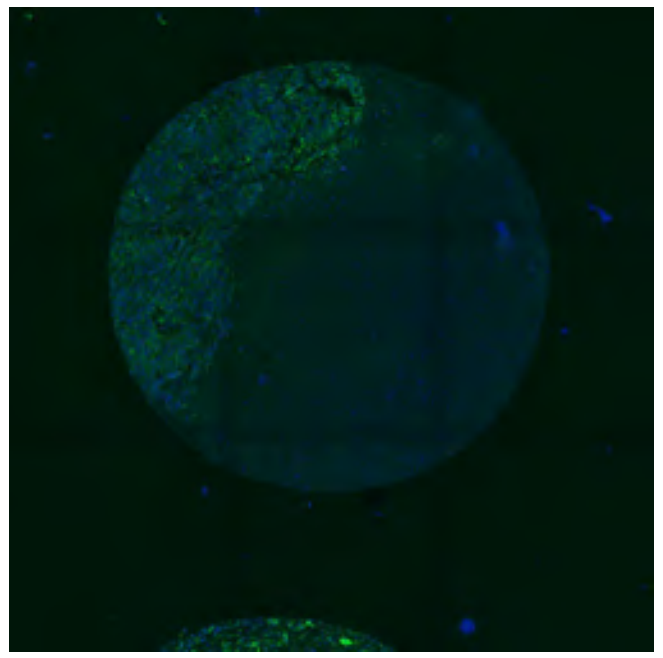

68
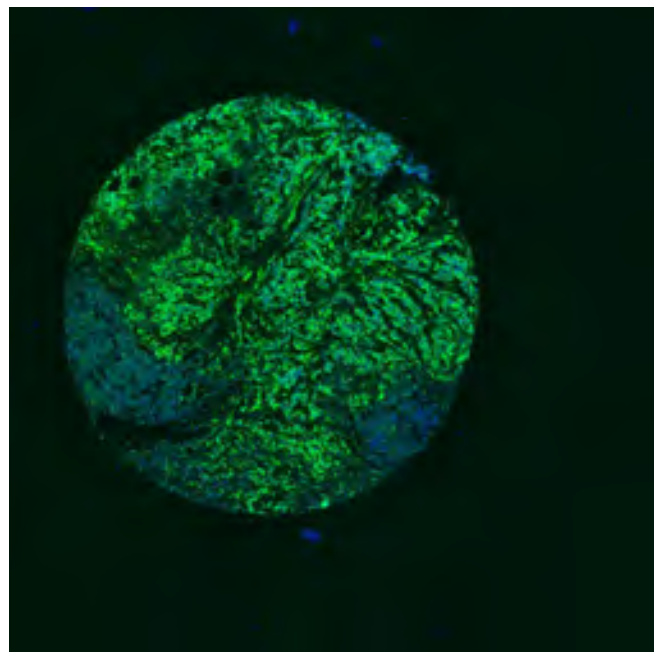

69
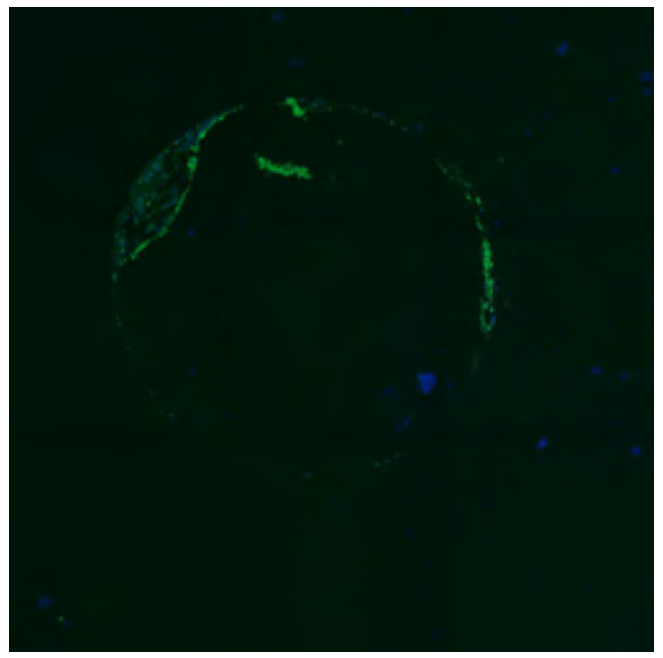

70
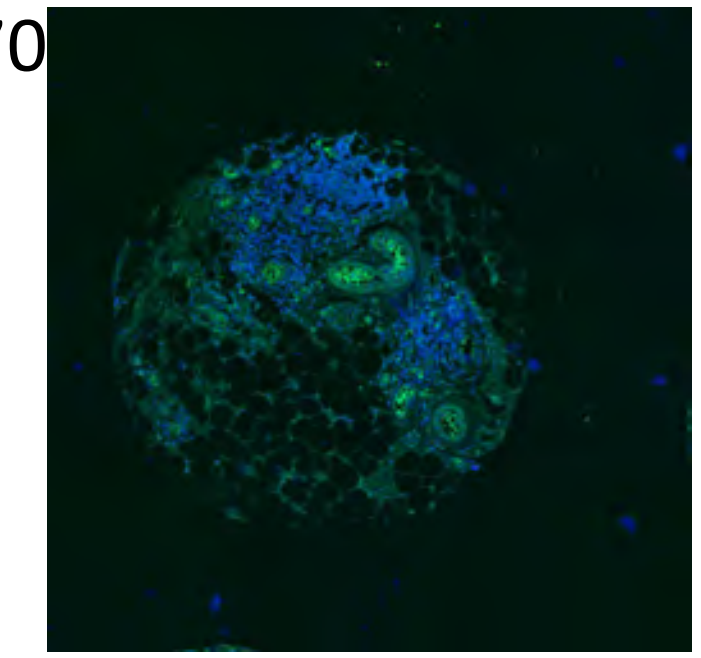

71

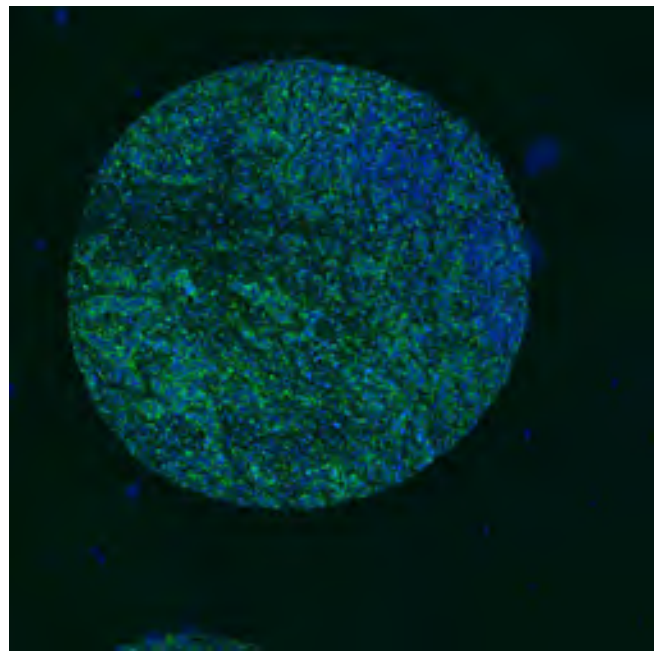

72

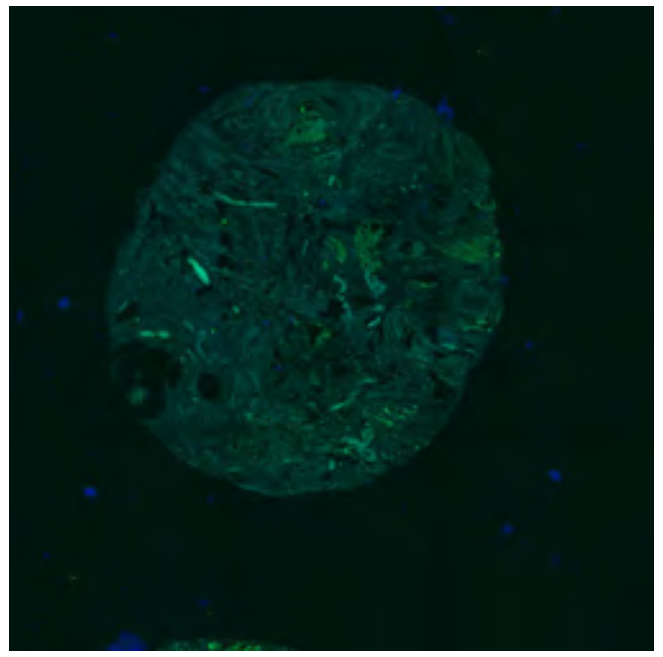

73

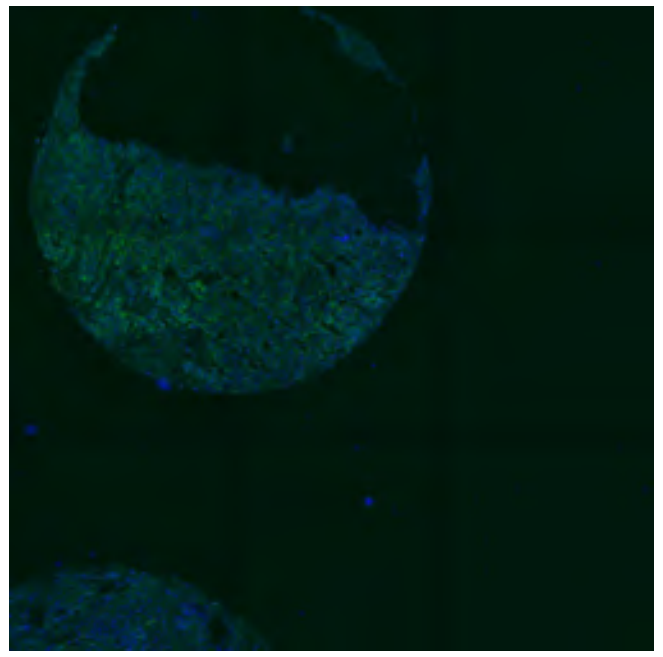

74

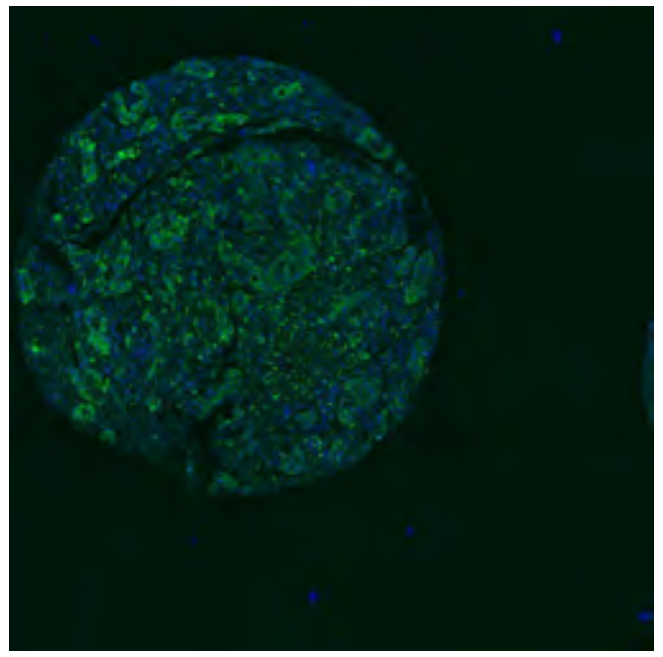

75

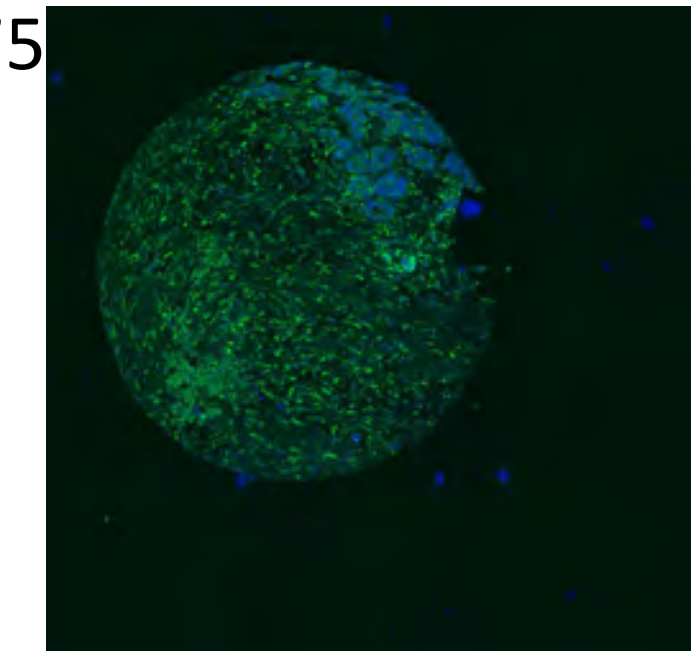

76

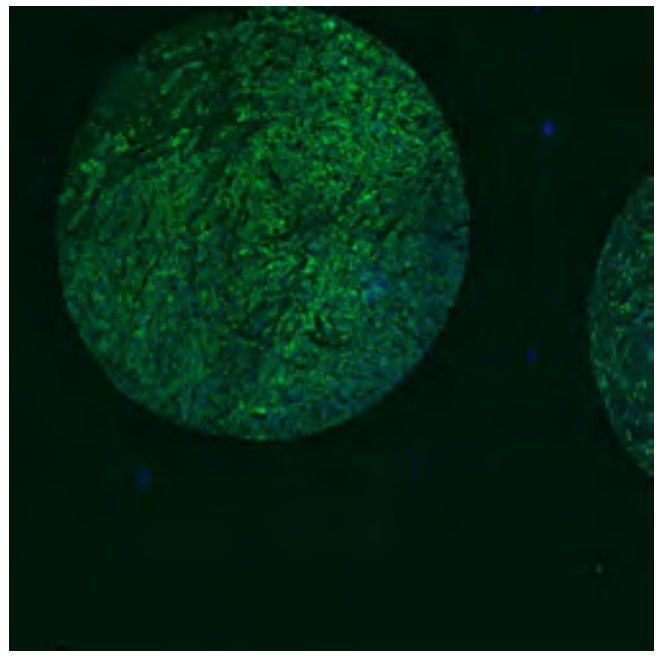

77

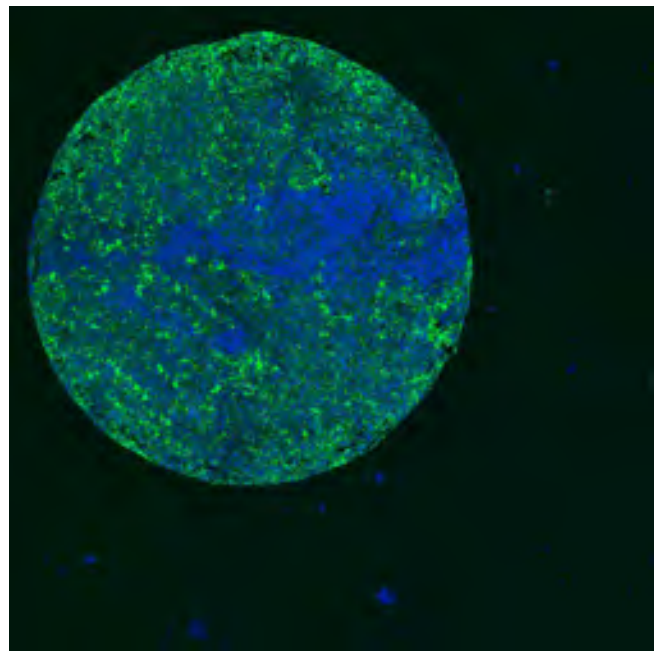

78

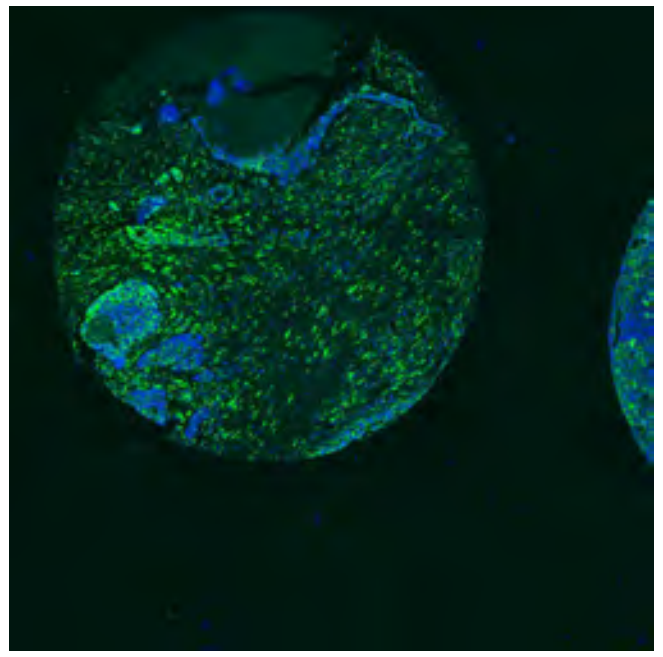

79

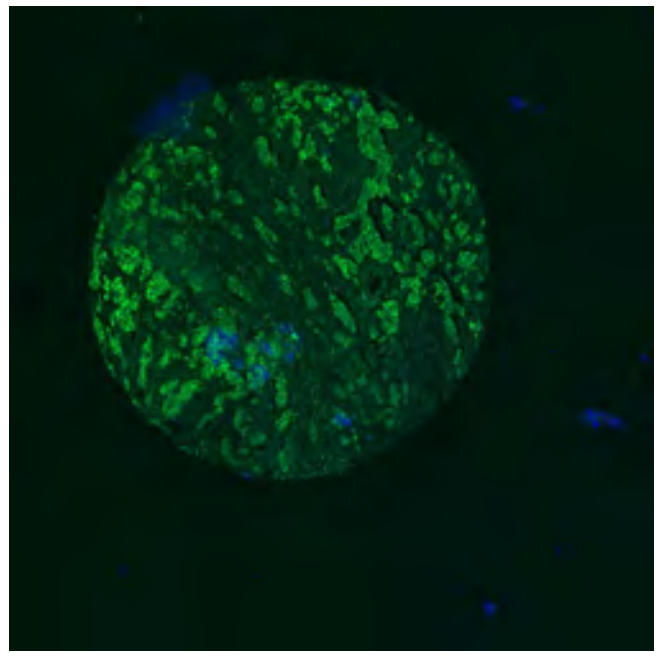

80

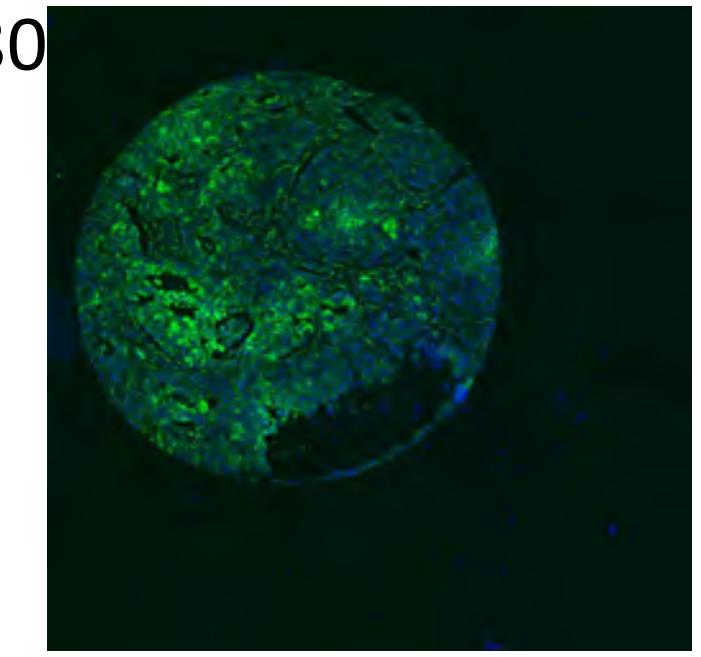

81

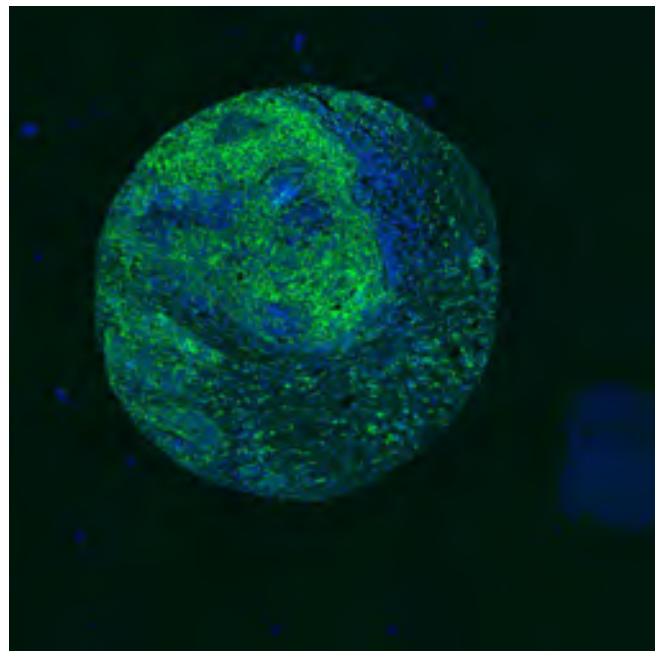

82

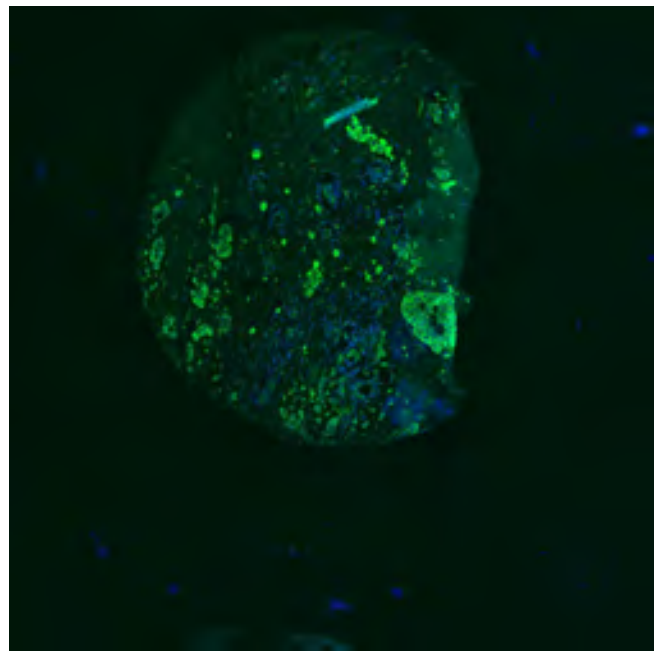

83

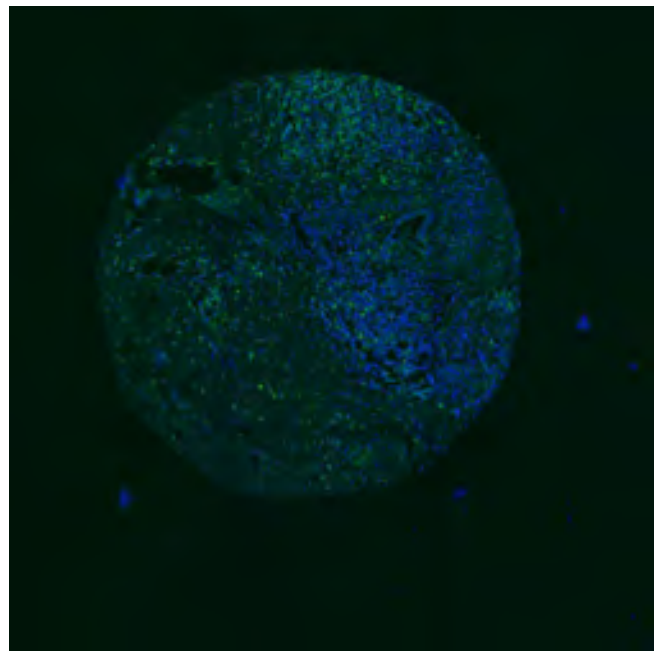

84

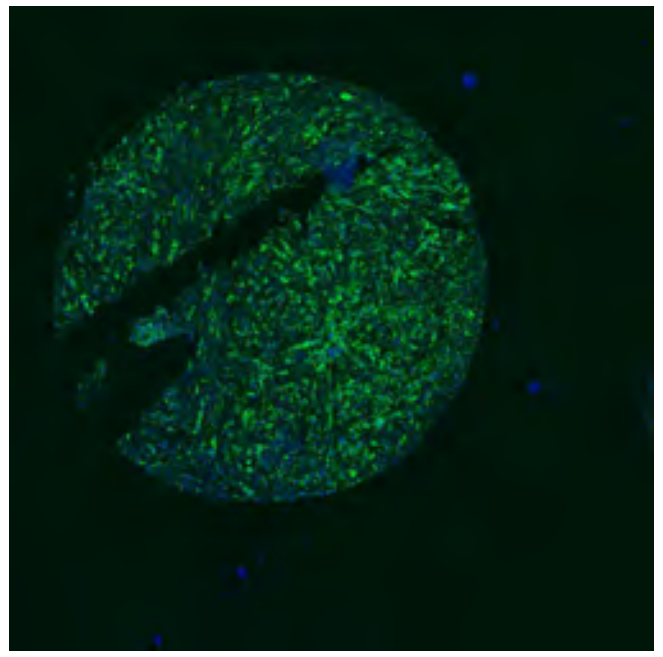

85

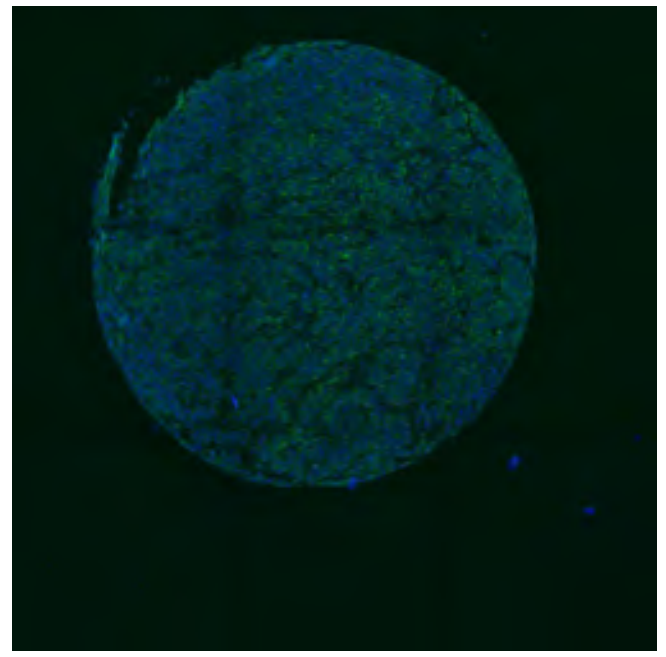

86

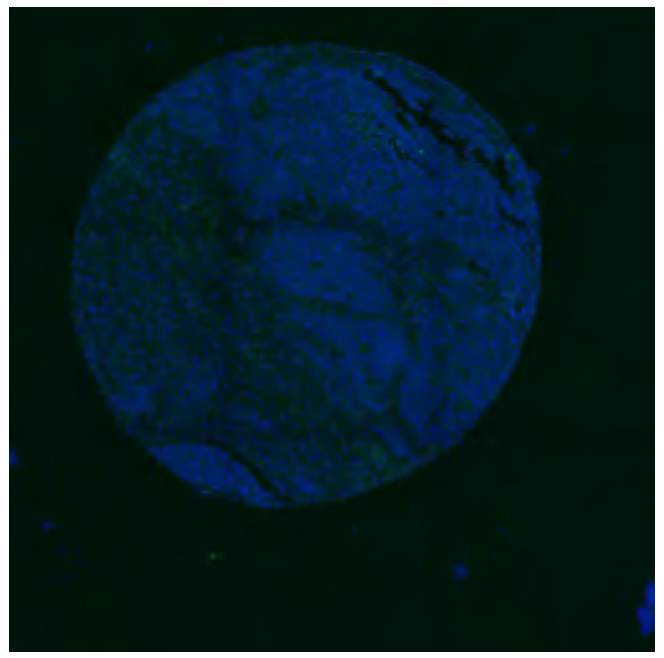

87

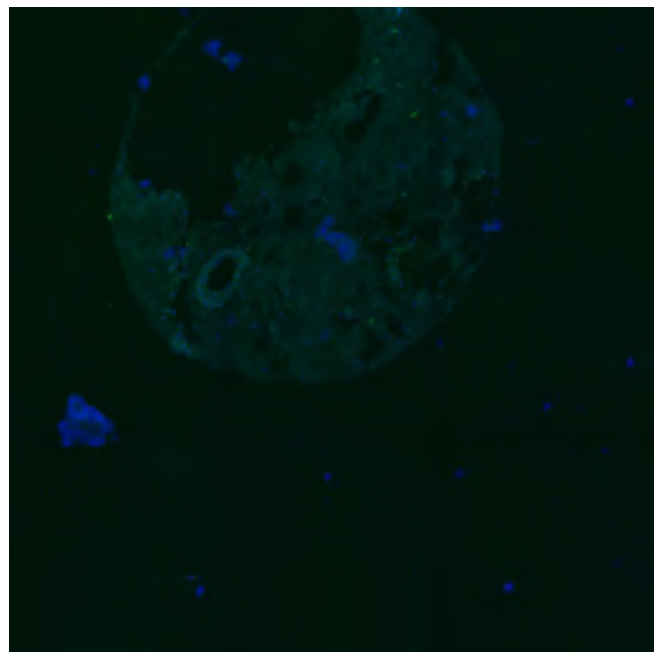

88

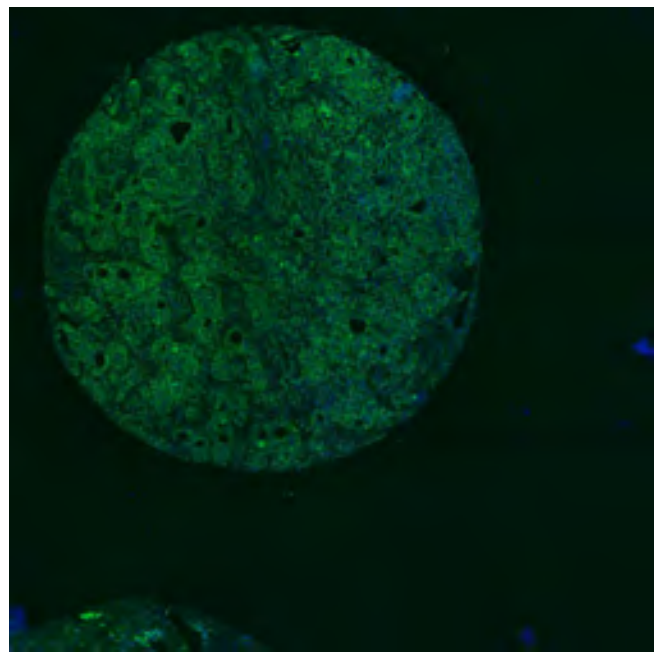

89

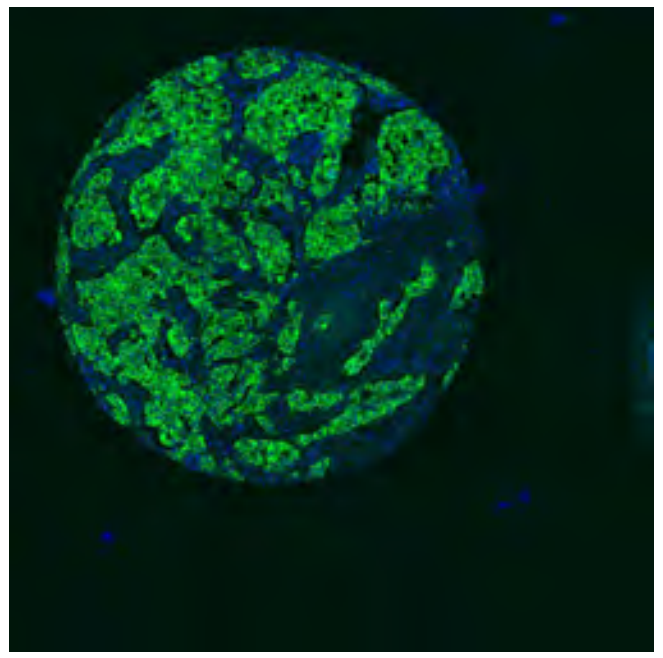

90

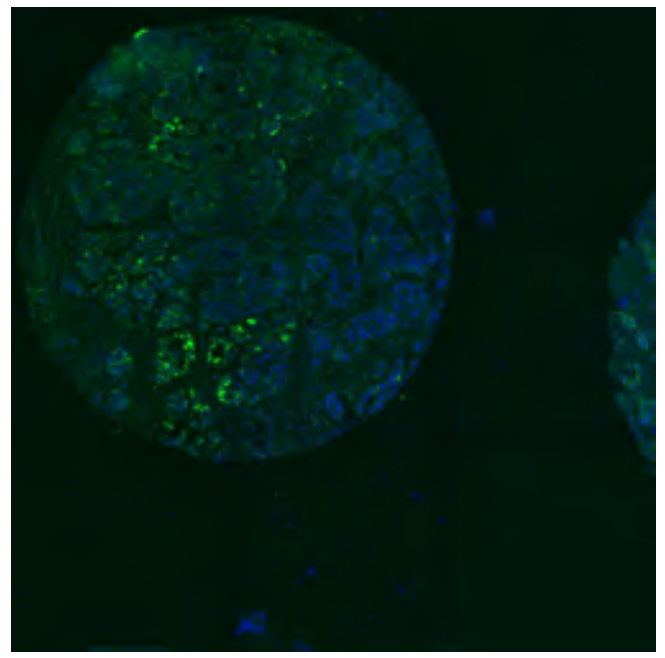

91
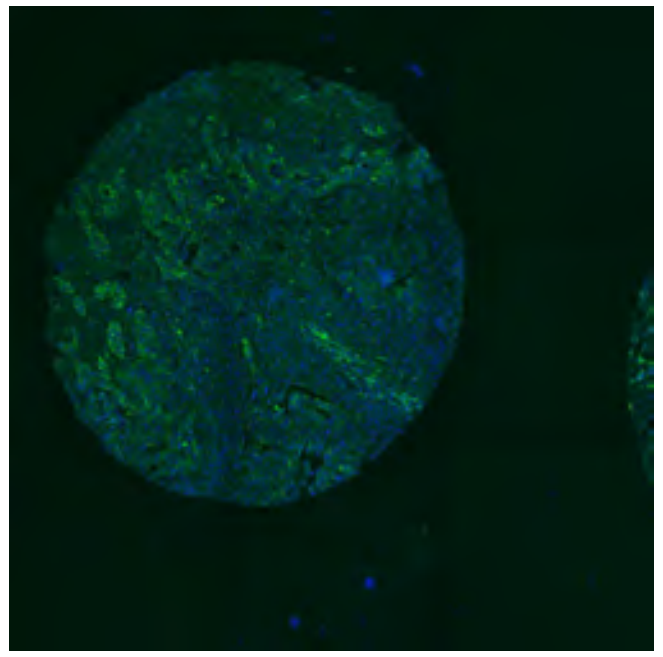

92
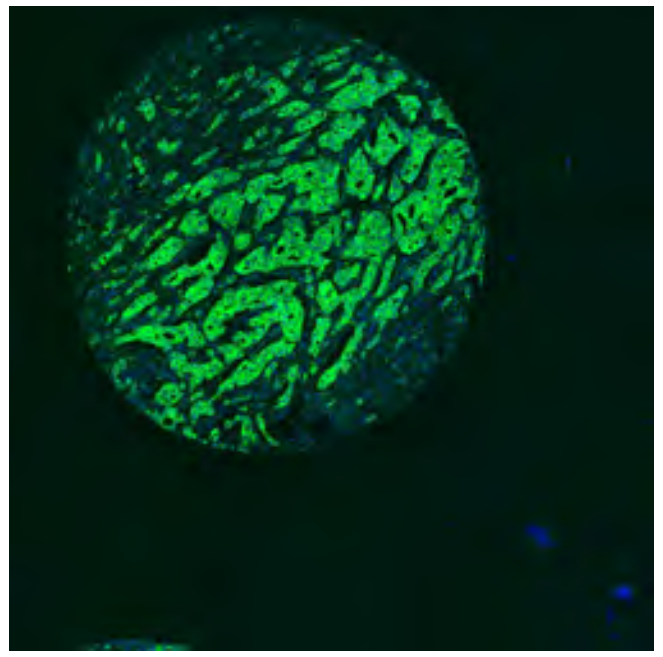

93
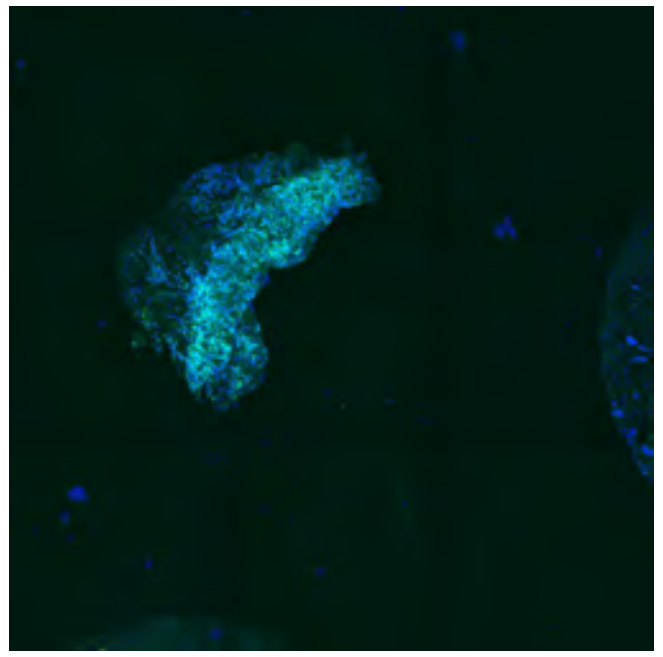

94
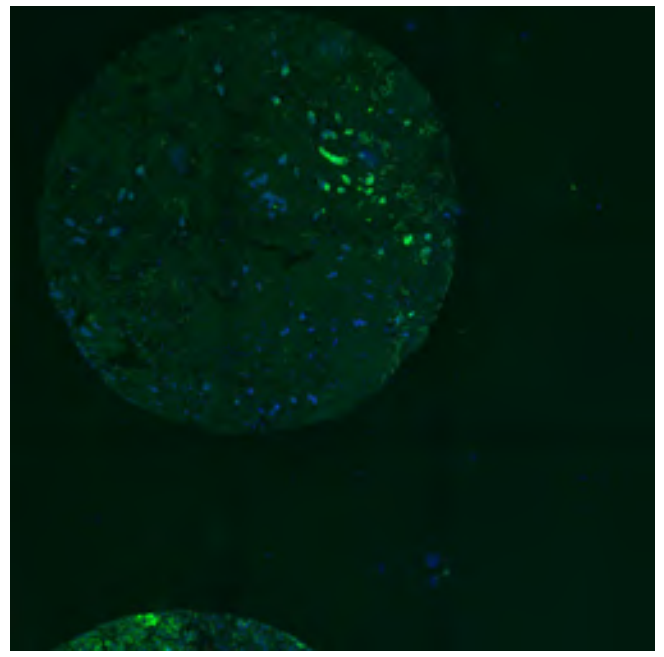

95
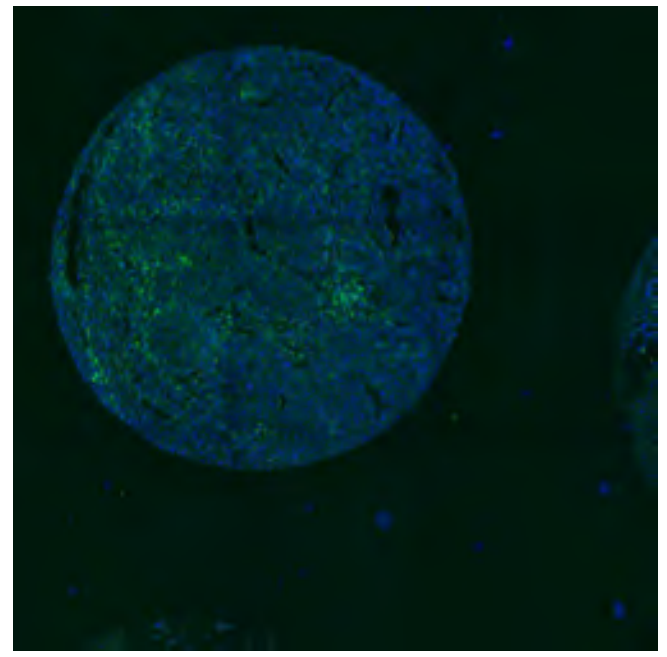

96
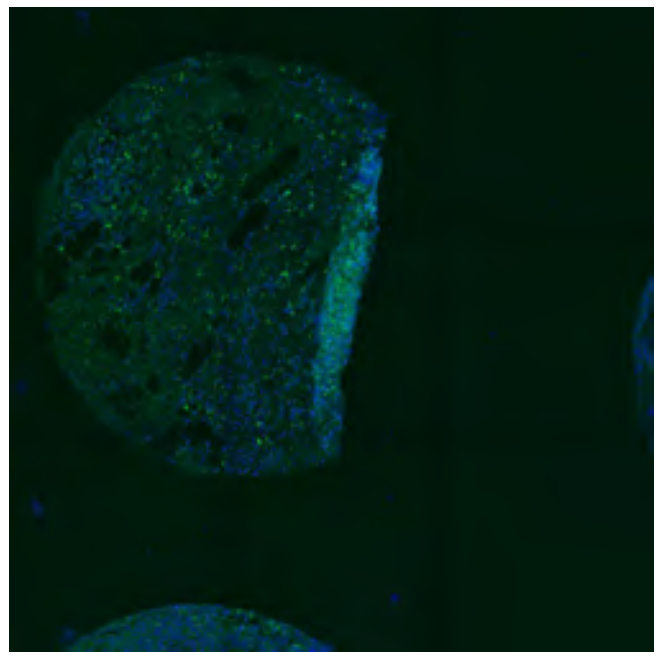

97
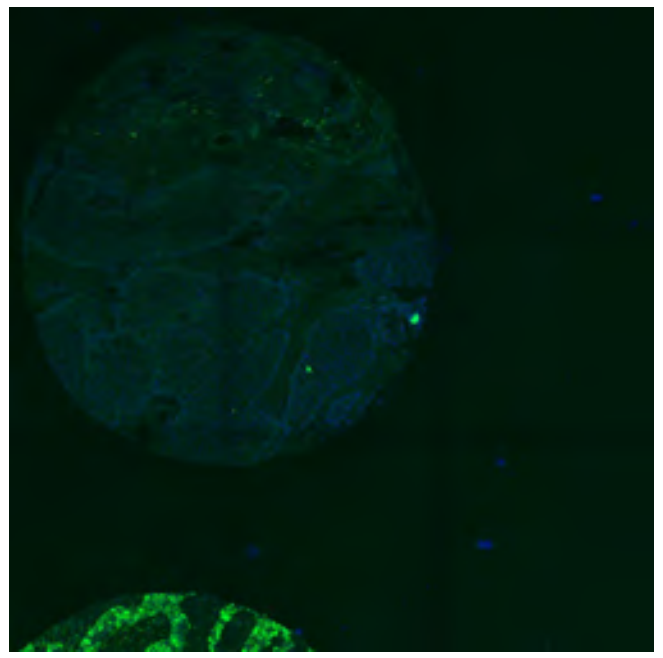

98
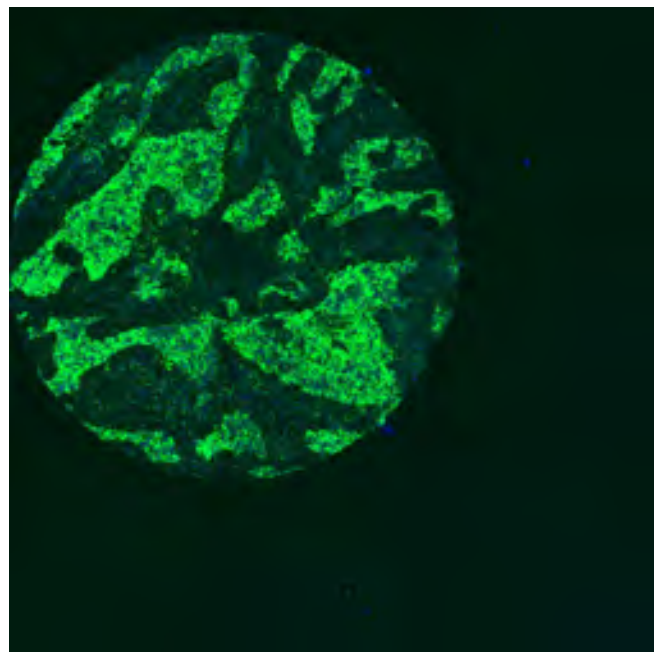

99
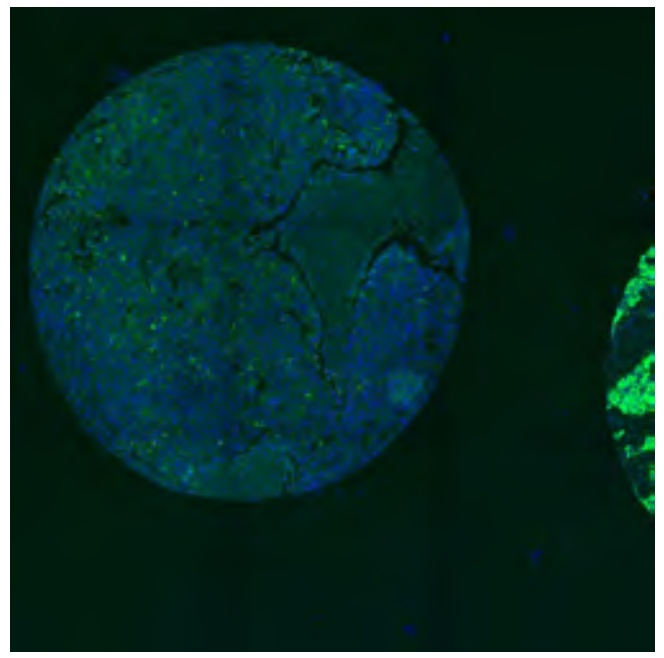

100
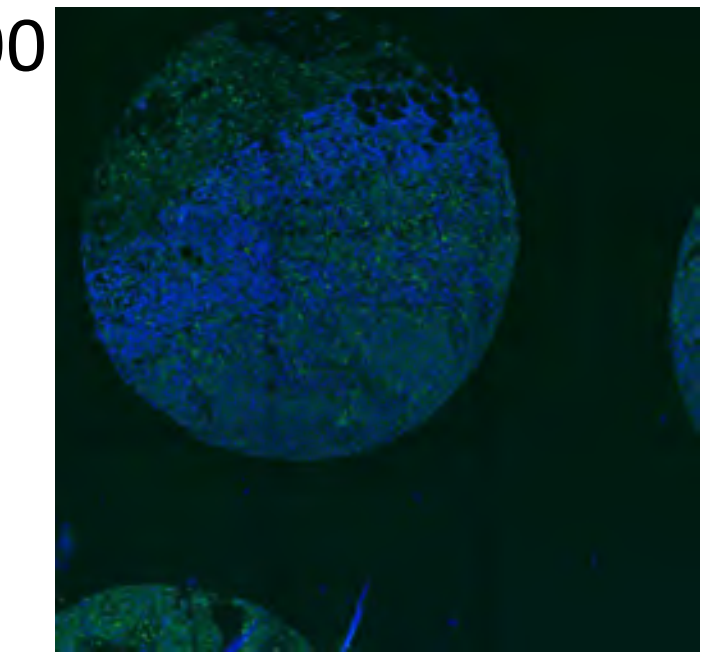

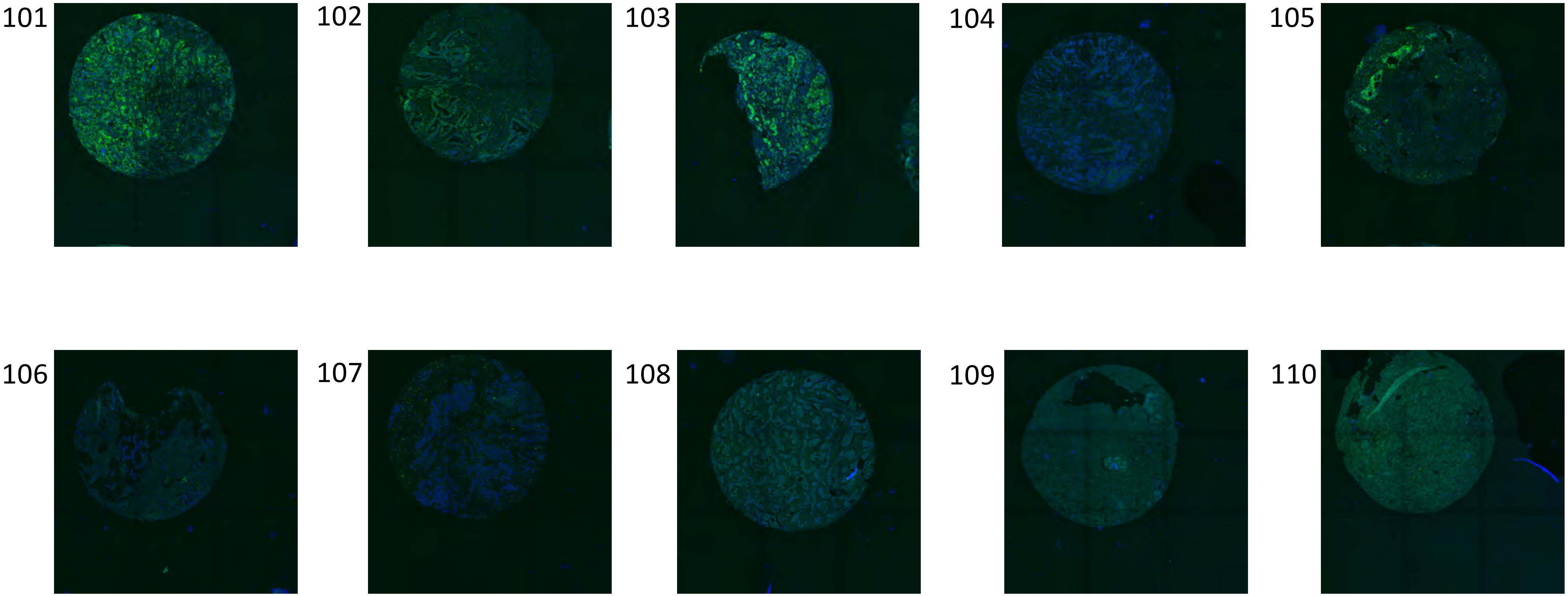
101
102
103
104
105
106
107
108
109
110

111

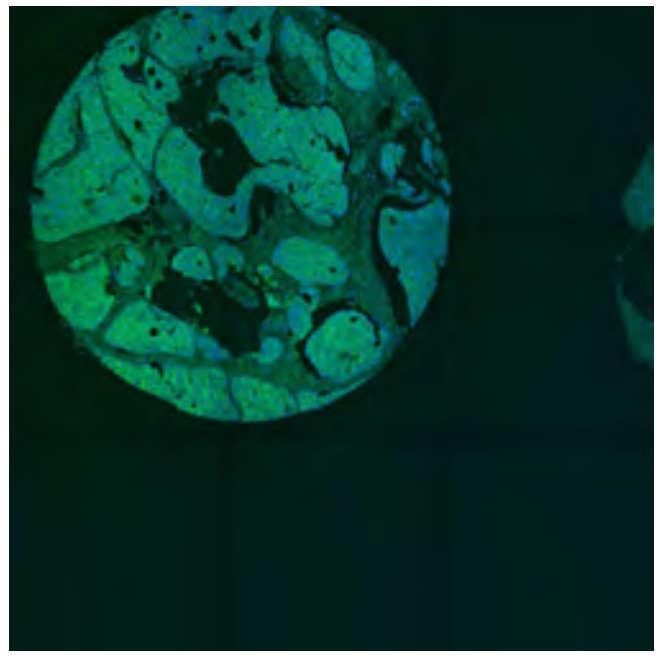

112

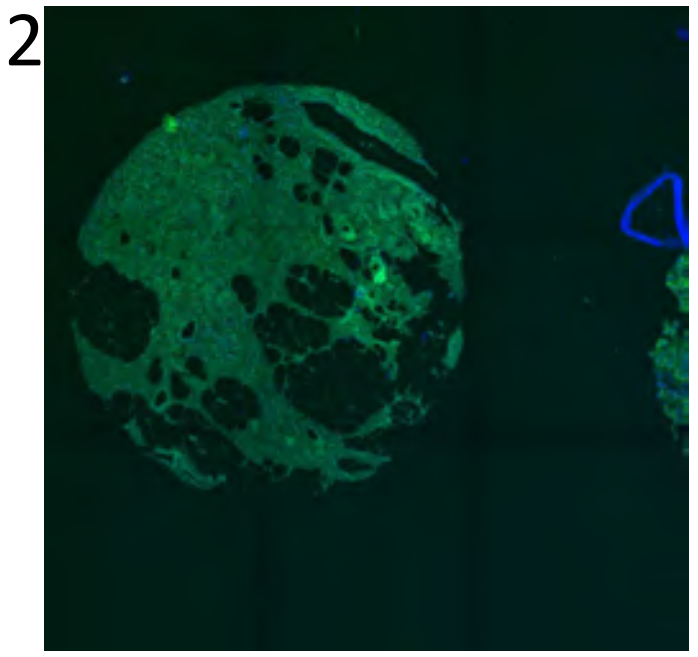

113

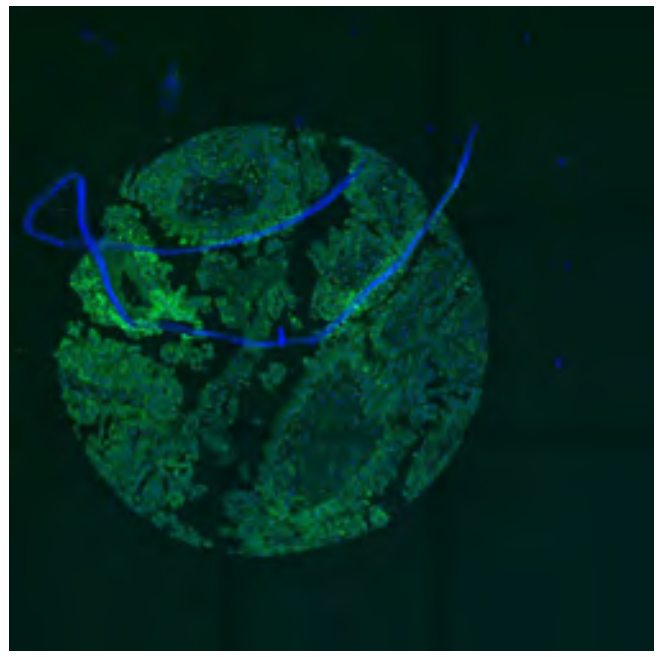

114

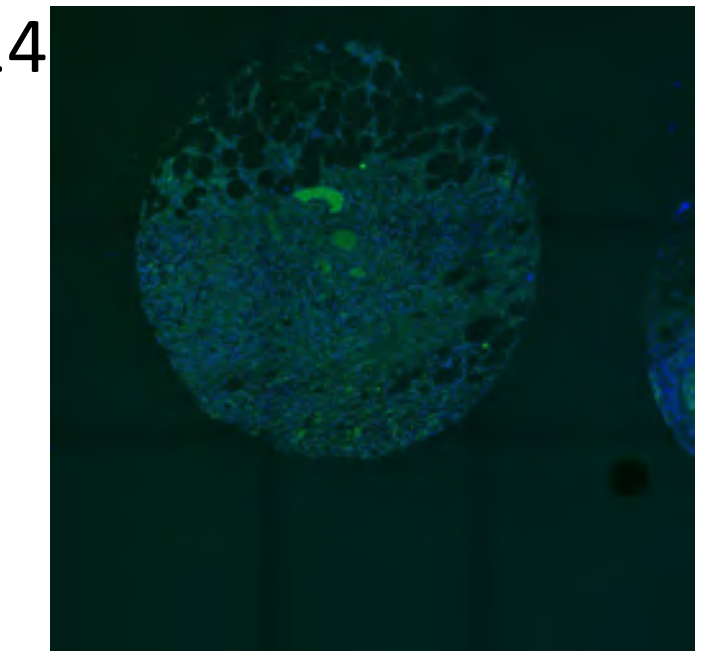

115

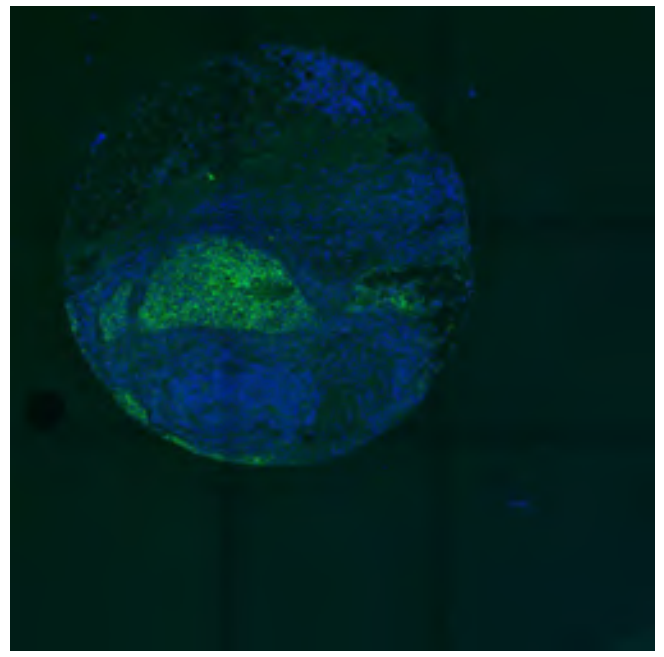

Figure Supplementary 2 linked to Figure 7

Uptake and MALDI-TOF/TOF analysis of mb7 encapsulated in liposomes.

(A) Titration curve of mb7 encapsulated in liposomes obtained after MALDI-TOF/TOF mass analysis using the Applied Biosystems 4700 Proteomics Analyzer. The spectrum shows a dominant peak at approximately 7 kDa corresponding to intact mb7, together with a smaller peak at approximately 3 kDa attributed to proteolytic cleavage products.

(B) MALDI-TOF/TOF mass spectrum of cellular extracts collected after 3 h incubation with mb7-loaded liposomes. The spectrum displays a predominant signal at approximately 3 kDa, corresponding to the cleaved form of the minibinder, representing approximately 40% of the initial mb7 signal detected prior to cellular uptake (please note the different scale between spectra).

(C) MALDI-TOF/TOF mass spectrum of cells treated with empty liposomes, showing no detectable mb7-related peaks.

Based on back-calculation analysis, approximately 50% of the initial mb7 was encapsulated into liposomes. Considering that the cleaved mb7 peak detected after uptake corresponds to less than 40% of the initial encapsulated signal, the intracellular uptake of mb7 from an initial theoretically 40 µM encapsulated concentration is estimated to be less than 8 µM.

A

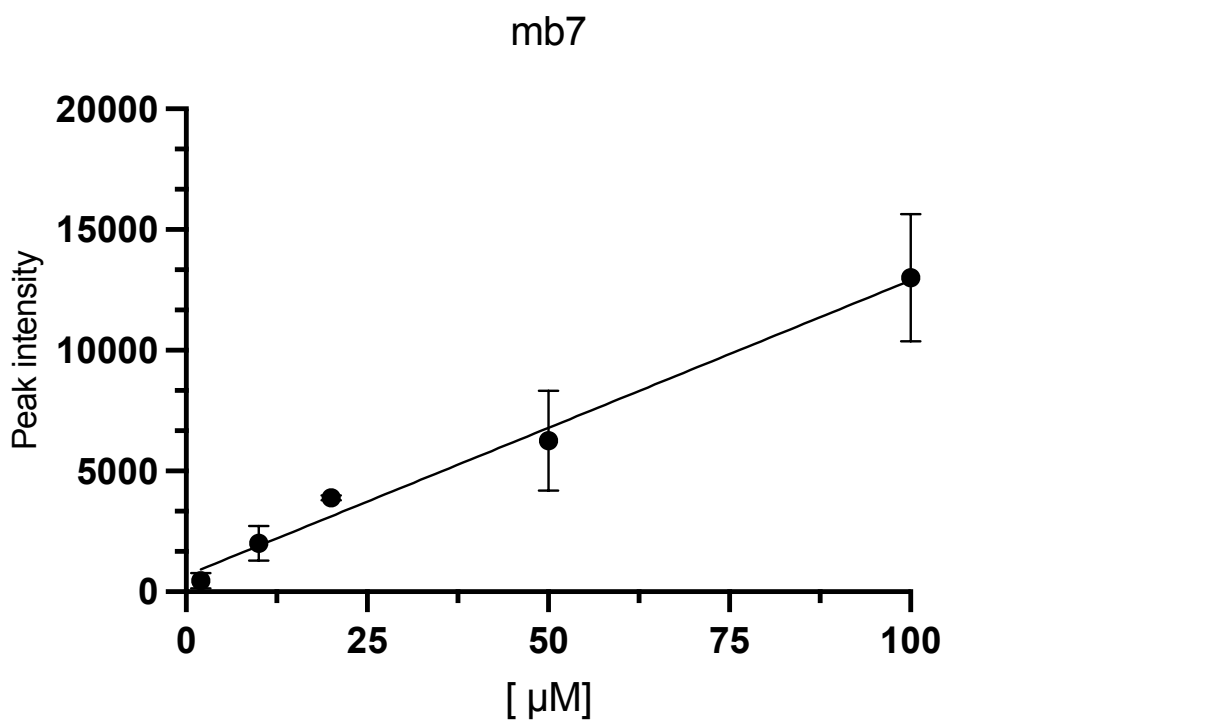

mb7 encapsulated in liposomes

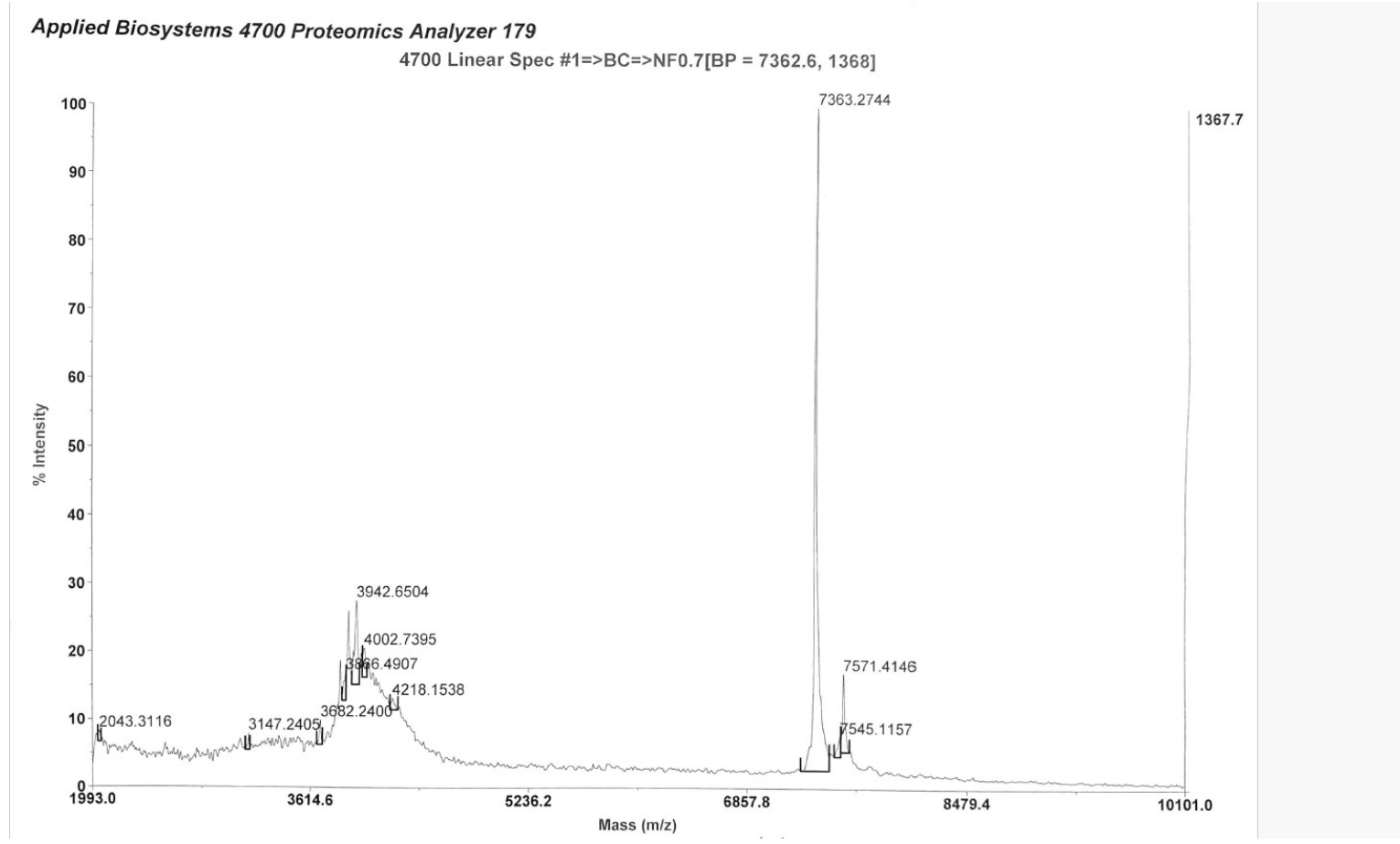

B

Cell sample treated with mb7 encapsulated liposomes

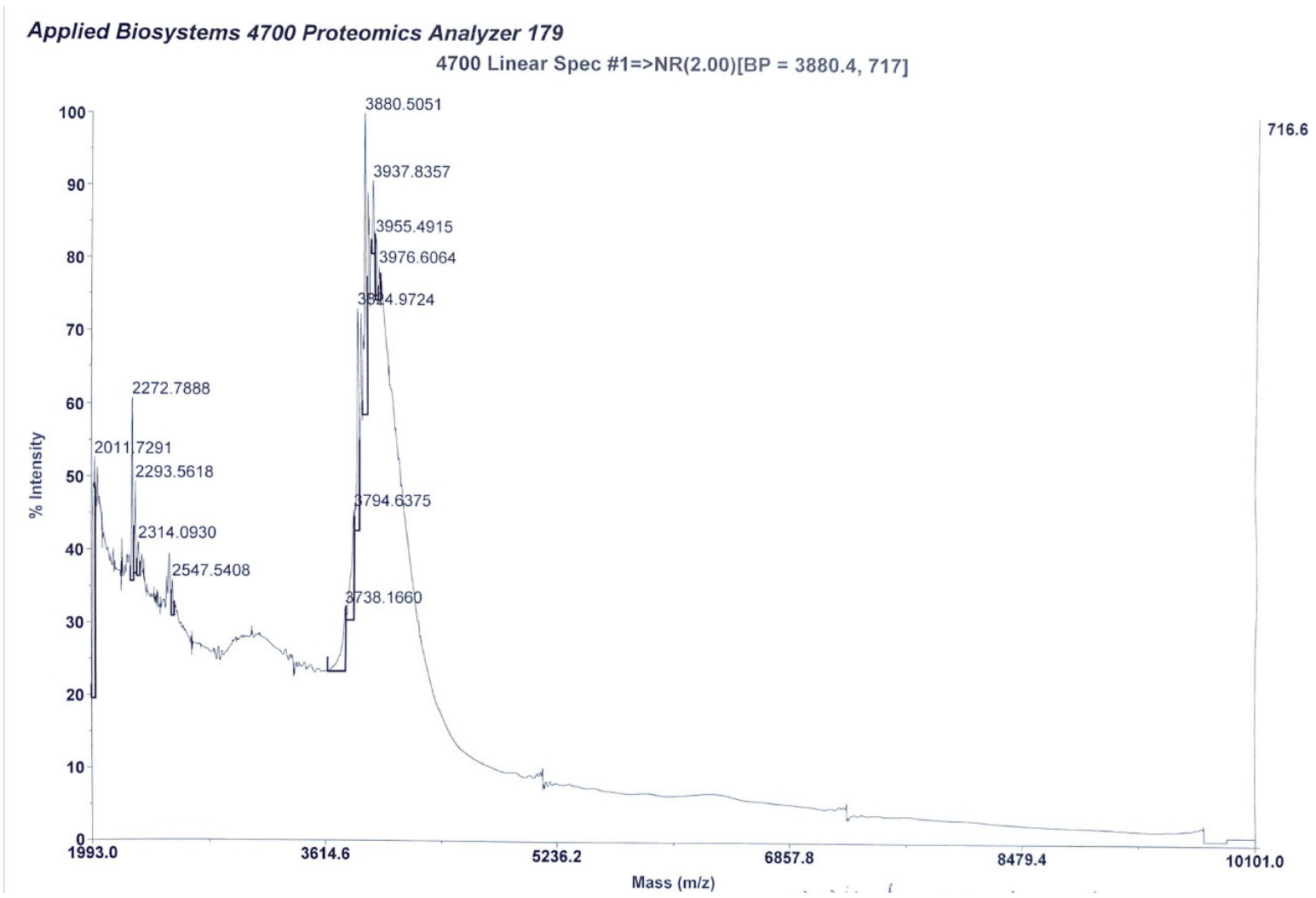

C

Cell sample treated with empty liposomes

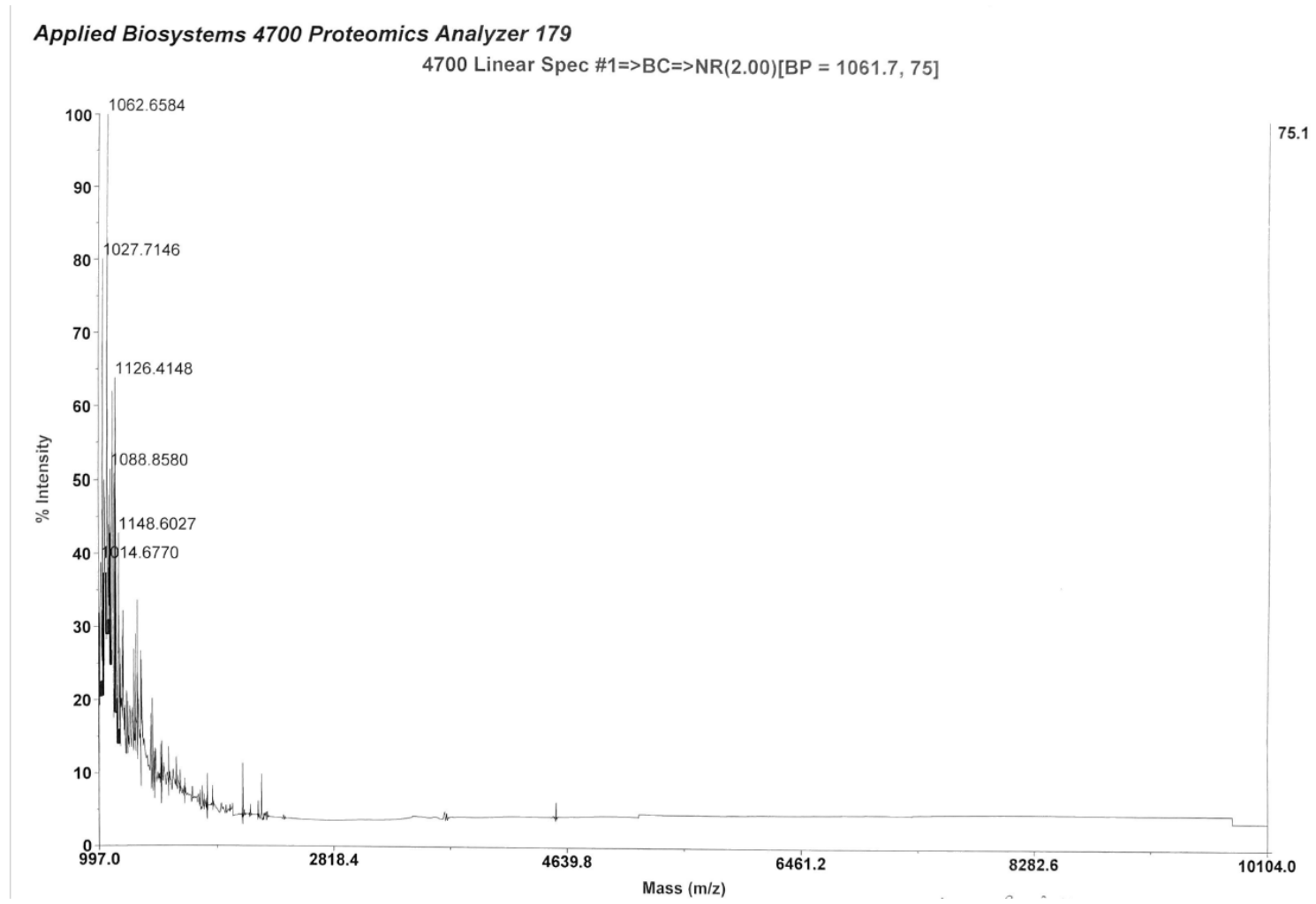

Supplement: Supplementary file 1 [file 41420_2026_3301_MOESM1_ESM.pdf]
